# Swedish intrauterine growth reference ranges of biometric measurements of fetal head, abdomen and femur

**DOI:** 10.1038/s41598-020-79797-8

**Published:** 2020-12-31

**Authors:** Linda Lindström, Mårten Ageheim, Ove Axelsson, Laith Hussain-Alkhateeb, Alkistis Skalkidou, Eva Bergman

**Affiliations:** 1 Department of Women’s and Children’s Health, Uppsala University, Uppsala, Sweden; 2 Centre for Clinical Research Sörmland, Uppsala University, Eskilstuna, Sweden; 3 Global Health, School of Public Health and Community Medicine, Institute of Medicine, Sahlgrenska Academy, University of Gothenburg, Gothenburg, Sweden

**Keywords:** Intrauterine growth, Health care

## Abstract

Ultrasonic assessment of fetal growth is an important part of obstetric care to prevent adverse pregnancy outcome. However, lack of reliable reference ranges is a major barrier for accurate interpretation of the examinations. The aim of this study was to create updated Swedish national reference ranges for intrauterine size and growth of the fetal head, abdomen and femur from gestational week 12 to 42. This prospective longitudinal multicentre study included 583 healthy pregnant women with low risk of aberrant fetal growth. Each woman was examined up to five times with ultrasound from gestational week 12 + 3 to 41 + 6. The assessed intrauterine fetal biometric measurements were biparietal diameter (outer–inner), head circumference, mean abdominal diameter, abdominal circumference and femur length. A two-level hierarchical regression model was employed to account for the individual measurements of the fetus and the number of repeated visits for measurements while accounting for the random effect of the identified parameterization of gestational age. The expected median and variance, expressed in both standard deviations and percentiles, for each individual biometric measurement was calculated. The presented national reference ranges can be used for assessment of intrauterine size and growth of the fetal head, abdomen and femur in the second and third trimester of pregnancy.

## Introduction

Fetal growth is an intricate process, depending on multiple factors, genetic as well as environmental. Fetal growth restriction is closely related to mortality and morbidity, with increased risks of short and long term complications^1^. A correct assessment of fetal size and growth is important to identify fetuses at risk of unfavourable outcomes^2^. In order to correctly assess fetal size, a reliable standard is of paramount importance.

An appropriate study design is vital when new standards for fetal size and growth are constructed, as fetal growth should not be assessed using charts that originate from cross-sectional data. Standards for fetal growth are instead ideally created from longitudinal data with repeated measurements over time in pregnancies with accurate pregnancy dating^3,4^. During the last decade, several international growth standards have been created^5–8^. However, doubts have been raised regarding the applicability of these international standards when evaluated in different populations^7,9–11^. It has therefore been advised that the international standards should be evaluated in each separate population before taken into general practice^5^.

The current Scandinavian growth charts, created in 1996 by Maršál et al., lack data on gestational ages below 25 weeks and are based on data from a rather small number of women, only 86, of whom 24% were smokers^12^. Since then, demographics of the Swedish pregnant population have changed, including e.g. higher maternal BMI, fewer smokers and higher birthweights^13^. Further, obstetric interventions are nowadays performed even in the second trimester of pregnancy, and children born preterm can be saved as early as gestational week 22. Hence, there is a pressing need for updated Swedish charts for fetal size and growth.

The aim of this study was to create updated national reference ranges for fetal size and growth from gestational week 12 to 42, by applying modern statistical methods to longitudinally collected data on ultrasonically derived intrauterine biometric measurements in a large Swedish cohort of low-risk pregnancies.

## Methods

### Study design and population

In this prospective longitudinal multicenter study, 684 women were recruited in early pregnancy between September 2015 and September 2018 in five sites in central Sweden; Uppsala, Falun, Katrineholm, Västerås and Örebro. At first antenatal visit, all women who received antenatal care at 18 representative primary care units were invited to participate. The participants were recruited from urban as well as rural areas. Healthy, non-smoking women were eligible if they had regular menstrual periods (28 ± 4 days) and no previous pregnancy complications, such as preterm birth, pre-eclampsia, eclampsia, gestational diabetes, hypertension and stillbirth. Women with chronic hypertension, systemic lupus erythematosus, kidney disease, diabetes, previous gastric bypass surgery or inflammatory bowel disease were not eligible, as these conditions are known to affect fetal growth.

At first study visit, between pregnancy week 12 + 3 and 13 + 6 according to the last menstrual period, gestational age (GA) was assessed with ultrasound. Only women with spontaneously conceived singleton pregnancies were recruited, and the discrepancy between GA according to the biparietal diameter (BPD) and GA according to last menstrual period was not allowed to exceed seven days. Seven experienced sonographers performed the study scans. Before first inclusion, all sonographers were given detailed instructions regarding how the biometric measurements should be performed.

At inclusion, the women were randomized to nine study protocols, according to the timing of the follow-up visits, in which each subject was assigned four follow-up ultrasound scans between gestational week 14 and 41. The study protocols were kept in closed envelopes and were randomly assigned to each study subject. The purpose was to receive evenly distributed measurements among the GAs. The vast majority of Swedish pregnant women undergo a routine second trimester ultrasound scan, usually taking place between week 17 and 20. In cases where the study subject was not randomized to a scan in week 17 to 20 (five protocols), biometric measurements were recorded in the study database if the scan was performed by a study sonographer. In order to compensate for the expected decline in the number of women with ongoing pregnancies, a larger number of study subjects were assigned to each group for the scans in week 37–41.

Women were excluded from the study if the pregnancy was complicated by gestational hypertension, pre-eclampsia or eclampsia, gestational diabetes, placenta previa, fetal malformations or chromosomal aberrations, stillbirth, fetal growth restriction with abnormal fetal Doppler or birth before 37 + 0 gestational weeks (259 gestational days).

### Procedures

The ultrasound machines used were GE Voluson E10, GE Voluson E8 and GE Voluson E6 with abdominal transducers 2–6 MHz RM6C, 2–8 MHz C4-8-D, RAB 4-8-D and 2–9 MHz C2-9-D. BPD was used to calculate the GA, using the modified Selbing and Kjessler formula, 58.65 + 1.07*BPD + 0.0138*BPD^2^, as recommended by the Swedish Society of Obstetrics and Gynecology^14^. Only fetuses with BPD at least 21 mm at first study visit were included. At each ultrasound scan, five biometric measurements were each measured three times; BPD, head circumference (HC), mean abdominal diameter (MAD), abdominal circumference (AC) and femur length (FL). All data was manually registered in a web-based study database.

BPD and HC were measured in an axial section, at the level of the thalami, with the midline echo in a central position broken anteriorly by cavum septum pellucidum. Orbitae and cerebellum were non-visible. The callipers for BPD were placed on the outer margin of the proximal parietal bone, and the inner margin of the distal parietal bone. HC was measured by placing the callipers on the outer borders of the frontal and occipital edges of the bone, and the ellipse facility was used to follow the contour of the skull. MAD and AC were measured in cross-section (circular view of the abdomen), with the stomach visible, the umbilical vein in the anterior third of the abdomen and the aorta and inferior vena cava anteriorly of the spine. Further, the greater part of a rib should be seen but not the heart or kidneys. The callipers for MAD were placed on the outer skin borders both anterioposteriorly and perpendicular transversely. AC was measured using the ellipse facility to follow the outer contours of the skin. Lastly, FL was measured in a longitudinal section of the femur in 45° to 90° angle of insonation, with the callipers placed on the outer borders of the femoral diaphysis. All measurements followed the national recommendations for biometric assessment and the practice guidelines from The International Society of Ultrasound in Obstetrics and Gynecology^14,15^.

### Data management

Each biometric measurement was estimated three times and registered in the study database for all GAs, totalling to 38,601 repeated measurements. Data was first examined graphically using scatter-plots of each biometric parameter for GA in order to identify deviant records and inspect some data assumptions. Outliers were identified and each outlier was inspected regarding GA and the value of the individual biometric parameters. GA was evaluated and corrected against wrong data entry in the database. Incorrect GA records were adjusted during the examination process according to estimated date of delivery and date of examination. Next, extreme or unreasonable measurements (such as HC equal to or smaller than BPD) were deleted or otherwise corrected, if original measurements were available in the woman’s medical records (often available for women examined in Uppsala, unlike other study sites). Where original measurements were considered unreasonable or contradictory, the corresponding data was deleted.

In 22 out of total 33 measurement records with incorrect GA, there was no information on GA. In the remaining eleven cases, GA was incorrectly calculated. A total of 267 measurements (0.68%) were outliers, with suspected incorrect entry of measurement values. Of the incorrect values, 166 (62%) were deleted. The remaining 101 incorrect values were corrected based on original measurement data. BPD was the measurement with lowest rate of incorrect values, 0.35%, followed by MAD with 0.41% incorrect values. AC was the measurement with the highest rate of incorrect values, 1.19%, followed by HC with a rate of 0.84%. FL was incorrectly entered in 0.64% of the measurements.

### Repeatability and reproducibility

A repeatability and reproducibility study was performed, where five study subjects were examined with repeated ultrasound scans during the same day. Six out of seven sonographers participated. Each study subject was examined two or three times by different sonographers, who assessed all five biometric measurements three times during each scan.

A linear mixed effect model was applied to estimate inter-observer variation. The chosen model accounts for the repeated measures and the differences in biometric measurements due to differences in GA. The model included fixed and random effects for each biometric measurement (BPD, HC, MAD, AC and FL), with a statistical marginal error of 5%.

In addition, the intraclass correlation coefficient (ICC) was assessed by applying a two-way mixed effects model to estimate intra-observer variation. We estimated absolute agreement, which includes systematic and random residual errors, for average measures.

### Statistical analysis

Descriptive statistics were used for maternal characteristics at baseline as well as for delivery and neonatal characteristics. An independent samples t-test was performed to evaluate if dating discrepancy was different for girls and boys. The t-test was used after confirming that the data does not violate the test assumptions. A one-way ANOVA was employed to evaluate if dating discrepancy varied between the study sites. An independent samples Mann–Whitney *U* test was performed to compare median birthweights in subgroups of the cohort.

The biometric measurements were used to create reference ranges for the individual variables (BPD, HC, MAD, HC and FL). The log transformed fetal growth measurements were modelled using a multilevel approach, with fixed and random effects. First, a fractional polynomial regression was performed on the log transformed fetal measurements to identify the best fitting combination of fractional polynomials for the GA. For instance for the fetal BPD, a combination of 0.5 and 3 as the best fitting fractional polynomial powers was identified. The derived parameters were then included in the regression model as fixed effects in a multilevel model to account for repeated measurements for each fetus. We followed the approach used by Ohuma and Altman^4^ and Johnsen et al.^16,17^—a two-level hierarchical model was used, considering the measurements (level 1) for each fetus (level 2) at each visit with a random effect for the effect of the identified fractional polynomial of GA and the intercept, similar to the study by Johnsen et al.^17^. We used the models mentioned above to estimate the expected fetal measurements at each GA in weeks. Thereafter, similar equations as in^17^ were used to compute the standard deviation (SD) and the percentiles while adjusting for maternal body mass index (BMI), height, parity, county of birth (Nordic or non-Nordic) and fetal sex.

In a sensitivity analysis, where women with abnormal BMI were excluded, we applied the same adjusted statistical models to a subset of the study cohort with BMI 18.5 to 29.9 kg/m^2^ to estimate the expected fetal biometric measurements at each GA in weeks, and to compute the SD and percentiles. The reference ranges before and after exclusion of women with abnormal BMI were compared using an independent samples t-test for each biometric measurement, for all subjects as well as stratified according to offspring sex.

Statistical analyses were performed using IBM SPSS Statistics version 2.5 and STATA 15.0.

## Results

Out of the 684 recruited women, 650 were eligible for the study. During pregnancy, 14 women (2.2%) developed hypertension or pre-eclampsia, and 11 (1.7%) developed diabetes and were hence excluded. Fetoplacental complications, such as placenta previa, placental abruption, single umbilical artery and preterm fetal growth restriction led to exclusion in six cases. One woman had a late miscarriage, one child was stillborn and 26 children were born preterm. Eight women were excluded due to fetal malformation or chromosomal aberration. Thus, the final cohort consisted of 583 women; 275 from Uppsala, 66 from Falun, 98 from Katrineholm, 50 from Västerås and 94 from Örebro.

**Figure 1:**
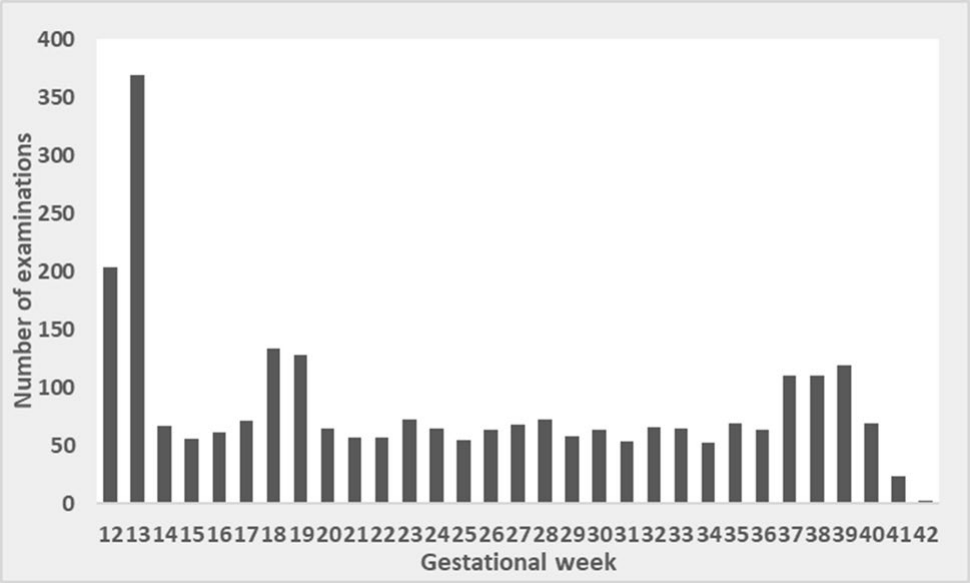
Distribution of ultrasound examinations by gestational age.

In total 2590 ultrasound scans were performed during the study. The majority, 526 of 583 included women, were scanned at least four times. In 187 women, all five planned ultrasound scans were performed. The ultrasound examinations following the dating scan were fairly equally distributed, see Fig. 1. There was a peak at week 18–19, corresponding to the routine ultrasound scan, and week 37–39. The dating discrepancy, i.e. the difference between estimated date of delivery (EDD) according to BPD at first study visit and EDD according to last menstrual period, was within ± 7 days, and thereby fulfilled the inclusion criteria. The mean discrepancy was − 0.1 days (SD 2.8 days) and the median discrepancy was 0 days. The dating discrepancy was slightly, but not statistically significantly, larger for girls than boys (p = 0.174); mean − 0.5 days for girls (SD 2.7 days) and 0.2 days for boys (SD 2.7 days), respectively. Further, there was a difference in dating discrepancy between the study sites (p < 0.001), with the lowest discrepancy in Katrineholm (mean 0.1 days, SD 2.4 days) and the largest in Västerås (mean − 1.2 days, SD 2.8 days).

The median age of the participating women was 29 years. BMI covered a range of 16.7–44.8 kg/m^2^, with a median BMI of 23.5 kg/m^2^. The majority of the study population, 92%, were born in a Nordic country (Sweden, Norway, Denmark, Finland or Iceland), and 5.5% were of non-European origin. Nearly 43% of the women were nulliparous. The median pregnancy duration was 281 days. Data on neonatal characteristics, including sex, was available for 574 children (98.5%). The median birthweight was 3625 g and median birth length 51 cm. For children with a mother born in a Nordic country, the median birthweight was 3628 g, compared with 3600 g for children with a mother born in a non-Nordic country; a difference that was not statistically significant (p = 0.258)*.* Likewise, the median birthweight was comparable in children with younger and older mothers; 3660 g for maternal age less than 35 years and 3624.5 g for maternal age 35 years or older, p = 0.908. Nulliparous women gave birth to children with lower median birthweight compared with parous women; 3540 g for nulliparous and 3714 g for parous women, p = 0.008. Maternal and neonatal characteristics are summarized in Table 1.

**Table 1:** Maternal and neonatal characteristics.

Parameter	Median (IQR)	Range	n (%)
Maternal age (years)	29 (26, 33)	19, 44	
Maternal height (cm)	167 (163, 171)	148, 187	
Weight first visit (kg)	66 (60, 75)	43, 146	
Body mass index first visit (kg/m^2^)	23.5 (21,6, 26,5)	16.7, 44.8	
Nordic country of birth			537 (92.1%)
Non-European country of birth			32 (5.5%)
Smoking first visit			0
Nulliparous			250 (42.9%)
Gestational age at inclusion (days)	92 (90, 94)	87, 101	
Pregnancy duration (days)	281 (276, 288)	259, 299	
Pregnancy duration (US)^a^ in women with spontaneous onset of labour (days)	282 (276, 288)	259, 298	
Pregnancy duration (LMP)^b^ in women with spontaneous onset of labour (days)	281 (276, 288)	257, 301	
Spontaneous vaginal delivery			458 (78.6%)
Induction of labour			40 (6.9%)
Caesarean section			77 (13.2%)
Newborn sex male			308 (52.8%)
APGAR < 7 at 5 min			6 (1.0%)
NICU admission > 1 day			56 (9.6%)
Neonatal death			0
Birthweight (g)	3625 (3344, 3925)	2366, 5100	
Birth length (cm)	51 (50, 52)	43, 57	

^a^Pregnancy duration according to ultrasound dating in gestational week 12 + 3 to 13 + 6.

^b^Pregnancy duration according to last menstrual period.

The median and variance of the different biometric measurements for each gestational week are shown in Tables 2, 3, 4, 5, 6, 7, 8, 9, 10, and 11. The variance is expressed in standard deviations (+ 3 SD, + 2 SD, + 1 SD, median, − 1 SD, − 2 SD and − 3 SD) and in percentiles (2.5th, 5th, 10th, 25th, median, 75th, 90th, 95th and 97.5th).

**Figure 2:**
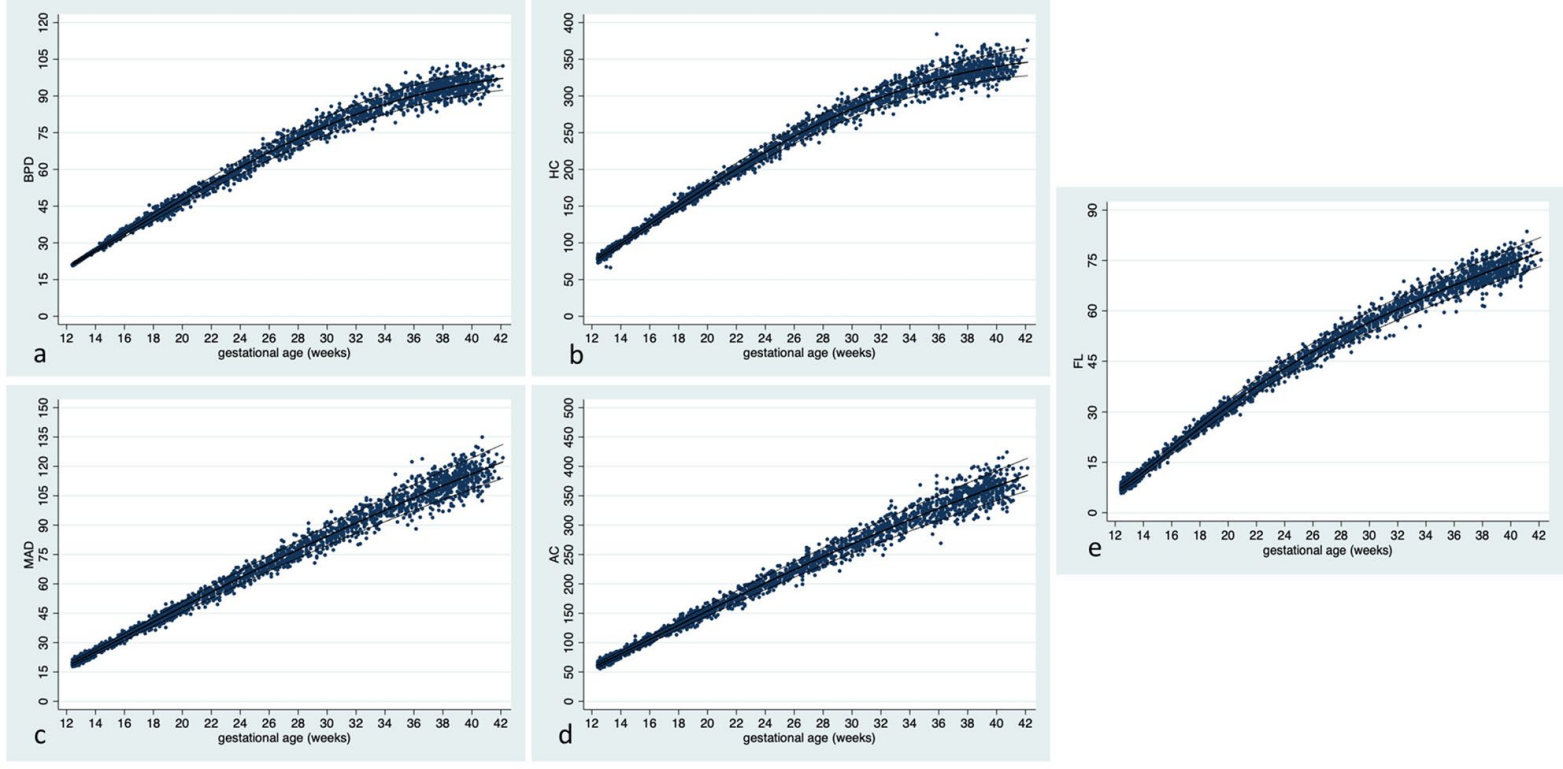
Raw data with fitted percentiles (10, 50, 90) for each estimated biometrical measurement in mm by gestational age (GA). (**a**) Shows biparietal diameter (BPD) by GA (n = 2586), (**b**) shows head circumference (HC) by GA (n = 2571), (**c**) shows mean abdominal diameter (MAD) by GA (n = 2585), (**d**) shows abdominal circumference (AC) by GA (n = 2561) and (**e**) shows femur length (FL) by GA (n = 2584).

Table 2 shows the median and variance for estimated BPD by GA in SD, and Table 3 BPD by GA in percentiles. Figure 2a shows the raw data with fitted percentiles for estimated BPD by GA.

**Table 2:** Estimated biparietal diameter (BPD) in mm by gestational age (GA) for males and females, standard deviations (SD).

GA (weeks^a^)	− 3 SD	− 2 SD	− 1 SD	Median	+ 1 SD	+ 2 SD	+ 3 SD
12	19	19	20	20	21	21	22
13	22	22	23	23	24	24	25
14	25	25	26	27	27	28	29
15	28	29	29	30	31	32	32
16	31	32	33	34	34	35	36
17	34	35	36	37	38	39	40
18	37	38	39	41	42	43	44
19	40	41	43	44	45	47	48
20	43	44	46	48	49	51	53
21	46	48	49	51	53	55	56
22	49	51	52	54	56	58	60
23	52	54	56	58	60	62	64
24	55	57	59	61	63	65	68
25	57	59	62	64	66	69	71
26	60	62	65	67	70	72	75
27	63	65	67	70	73	75	78
28	65	67	70	73	75	78	81
29	67	70	73	75	78	81	84
30	70	72	75	78	81	84	87
31	72	75	77	80	83	86	90
32	74	77	79	82	86	89	92
33	76	79	82	85	88	91	94
34	77	80	83	87	90	93	97
35	79	82	85	88	92	95	99
36	81	84	87	90	93	97	101
37	82	85	88	92	95	99	102
38	83	86	90	93	96	100	104
39	84	87	91	94	98	102	105
40	85	88	92	95	99	103	107
41	86	89	93	96	100	104	108
42	86	90	93	97	101	105	109

^a^GA expressed as completed gestational weeks, e.g. 12 weeks corresponds to 12 + 0 weeks or 84 gestational days.

**Table 3:** Estimated biparietal diameter (BPD) in mm by gestational age (GA) for males and females, percentiles.

GA (weeks^a^)	2.5th	5th	10th	25th	Median	75th	90th	95th	97.5th
12	19	19	20	20	20	21	21	21	21
13	22	23	23	23	23	24	24	24	24
14	25	26	26	26	27	27	28	28	28
15	29	29	29	30	30	31	31	31	32
16	32	32	32	33	34	34	35	35	35
17	35	35	36	36	37	38	38	39	39
18	38	39	39	40	41	41	42	43	43
19	41	42	42	43	44	45	46	46	47
20	45	45	46	46	48	49	50	50	51
21	48	48	49	50	51	52	53	54	55
22	51	51	52	53	54	56	57	58	58
23	54	54	55	56	58	59	60	61	62
24	57	57	58	59	61	62	64	65	65
25	60	60	61	62	64	66	67	68	69
26	62	63	64	65	67	69	70	71	72
27	65	66	67	68	70	72	73	74	75
28	68	68	69	71	73	75	76	77	78
29	70	71	72	73	75	77	79	80	81
30	72	73	74	76	78	80	82	83	84
31	75	75	77	78	80	82	84	85	86
32	77	78	79	80	82	85	86	88	89
33	79	80	81	83	85	87	89	90	91
34	81	81	83	84	87	89	91	92	93
35	82	83	84	86	88	91	93	94	95
36	84	85	86	88	90	92	94	96	97
37	85	86	87	89	92	94	96	97	98
38	86	87	89	91	93	95	97	99	100
39	87	89	90	92	94	97	99	100	101
40	88	89	91	93	95	98	100	101	103
41	89	90	92	94	96	99	101	103	104
42	90	91	92	95	97	100	102	104	105

^a^GA expressed as completed gestational weeks, e.g. 12 weeks corresponds to 12 + 0 weeks or 84 gestational days.

Mean and variance equation for BPD in males and females:

$$E\left( {Z_{i} } \right) = \, - {2}.{53 } + \, \left[ {{2}.{\text{47 log}}\left( {{\text{GA}}_{{\text{i}}} } \right)} \right] \, + \, \left[ { - 0.0{\text{5 GA}}_{{\text{i}}}^{{1}} } \right]$$

$$Var\left( {Zi} \right) = \, 0.0{8 } + \, \left[ {0.0{\text{2 log}}\left( {{\text{GA}}_{{\text{i}}} } \right)^{{2}} } \right] \, + \, \left[ { - 0.0{\text{8 log}}\left( {{\text{GA}}_{{\text{i}}} } \right)} \right] \, + \, \left[ {0.00{\text{3 GA}}_{{\text{i}}}^{{1}} } \right] \, + \, \left[ {-0.000{\text{2 log}}\left( {{\text{GA}}_{{\text{i}}} } \right){\text{GA}}_{{\text{i}}}^{{1}} } \right] \, + \, \left[ {0.0000{\text{4 GA}}_{{\text{i}}}^{{2}} } \right]$$

Table 4 shows the median and variance for estimated HC by GA in SD, and Table 5 HC by GA in percentiles. Figure 2b shows the raw data with fitted percentiles for estimated HC by GA.

**Table 4:** Estimated head circumference (HC) in mm by gestational age (GA) for males and females, standard deviations (SD).

GA (weeks^a^)	− 3 SD	− 2 SD	− 1 SD	Median	+ 1 SD	+ 2 SD	+ 3 SD
12	66	69	71	74	76	79	82
13	78	81	83	86	89	92	95
14	90	93	96	99	102	105	108
15	102	105	108	112	115	118	122
16	114	118	121	125	128	132	136
17	126	130	134	138	142	146	150
18	138	142	146	151	155	160	164
19	150	154	159	164	168	173	179
20	161	166	171	176	181	187	192
21	172	178	183	188	194	200	206
22	183	189	195	201	207	213	220
23	194	200	206	212	219	226	233
24	204	210	217	224	231	238	245
25	213	220	227	234	242	250	258
26	223	230	237	245	253	261	270
27	231	239	247	255	263	272	281
28	240	248	256	265	273	282	292
29	248	256	265	274	283	292	302
30	255	264	273	282	292	302	312
31	262	271	281	290	300	311	321
32	269	278	288	298	308	319	330
33	275	285	295	305	316	327	338
34	280	290	301	312	323	334	346
35	285	296	306	318	329	341	353
36	290	301	312	323	335	347	360
37	294	305	316	328	340	353	367
38	297	308	320	333	345	359	373
39	300	311	324	337	350	364	378
40	302	314	327	340	354	368	383
41	303	316	329	343	357	372	388
42	304	318	331	346	361	376	392

^a^GA expressed as completed gestational weeks, e.g. 12 weeks corresponds to 12 + 0 weeks or 84 gestational days.

**Table 5:** Estimated head circumference (HC) in mm by gestational age (GA) for males and females, percentiles.

GA (weeks^a^)	2.5th	5th	10th	25th	Median	75th	90th	95th	97.5th
12	69	69	70	72	74	75	77	78	79
13	81	82	83	84	86	88	90	91	92
14	93	94	95	97	99	101	103	104	105
15	105	106	108	110	112	114	116	117	118
16	118	119	120	122	125	127	129	131	132
17	130	131	133	135	138	140	143	144	146
18	142	144	145	148	151	154	156	158	160
19	154	156	158	160	164	167	170	172	173
20	166	168	170	173	176	180	183	185	187
21	178	179	181	185	188	192	196	198	200
22	189	191	193	196	201	205	208	211	213
23	200	202	204	208	212	217	221	223	225
24	210	212	215	219	224	228	233	235	238
25	220	223	225	230	234	239	244	247	249
26	230	232	235	240	245	250	255	258	261
27	239	242	245	250	255	261	266	269	272
28	248	251	254	259	265	270	276	279	282
29	257	259	262	268	274	280	285	289	292
30	264	267	270	276	282	289	295	298	301
31	272	275	278	284	290	297	303	307	310
32	279	282	285	291	298	305	311	315	318
33	285	288	292	298	305	312	319	323	326
34	291	294	298	304	312	319	326	330	334
35	296	300	303	310	318	325	332	337	341
36	301	304	308	315	323	331	338	343	347
37	305	309	313	320	328	336	344	349	353
38	309	313	317	324	333	341	349	354	358
39	312	316	320	328	337	346	354	359	363
40	315	319	323	331	340	349	358	363	368
41	317	321	325	334	343	353	362	367	372
42	318	322	327	336	346	356	365	371	376

^a^GA expressed as completed gestational weeks, e.g. 12 weeks corresponds to 12 + 0 weeks or 84 gestational days.

Mean and variance equation for HC in males and females:

$$E\left( {Zi} \right) = { 8}.{48 } + \, \left[ { - {14}.{\text{36 GA}}_{{\text{i}}}^{{ - 0.{5}}} } \right] \, + \, \left[ { - 0.000{\text{2 GA}}_{{\text{i}}}^{{2}} } \right]$$

$$Var\left( {Zi} \right) = \, 0.0{2 } + \, \left[ {0.{\text{25 GA}}_{{\text{i}}}^{{ - {1}}} } \right] \, + \, \left[ { - 0.{\text{13 GA}}_{{\text{i}}}^{{ - 0.{5}}} } \right] \, + \, \left[ { - {8}.{\text{24e}} - 0{\text{6 GA}}_{{\text{i}}}^{{2}} } \right] \, + \, \left[ {0.0000{\text{3 GA}}_{{\text{i}}}^{{ - 0.{5}}} {\text{GA}}_{{\text{i}}}^{{2}} } \right] \, + \, \left[ {{1}.{\text{43e}} - 0{\text{9 GA}}_{{\text{i}}}^{{4}} } \right]$$

Table 6 shows the median and variance for estimated MAD by GA in SD, and Table 7 MAD by GA in percentiles. Figure 2c shows the raw data with fitted percentiles for estimated MAD by GA.

**Table 6:** Estimated mean abdominal diameter (MAD) in mm by gestational age (GA) for males and females, standard deviations (SD).

GA (weeks^a^)	− 3 SD	− 2 SD	− 1 SD	Median	+ 1 SD	+ 2 SD	+ 3 SD
12	15	16	17	18	19	20	21
13	19	20	21	22	23	24	25
14	22	23	24	25	26	28	29
15	26	27	28	29	30	32	33
16	29	30	32	33	34	36	37
17	32	34	35	37	38	40	42
18	36	37	39	41	42	44	46
19	39	41	43	44	46	48	50
20	42	44	46	48	50	52	55
21	46	48	50	52	54	57	59
22	49	51	53	56	58	61	63
23	52	55	57	59	62	65	68
24	56	58	61	63	66	69	72
25	59	61	64	67	70	73	76
26	62	65	67	70	73	77	80
27	65	68	71	74	77	81	84
28	68	71	74	77	81	85	88
29	71	74	77	81	85	88	92
30	74	77	81	84	88	92	97
31	77	80	84	88	92	96	101
32	79	83	87	91	95	100	105
33	82	86	90	94	99	104	109
34	85	89	93	98	102	107	113
35	87	91	96	101	106	111	117
36	90	94	99	104	109	115	121
37	92	97	102	107	113	118	124
38	94	99	105	110	116	122	128
39	97	102	107	113	119	126	132
40	99	104	110	116	122	129	136
41	101	107	113	119	126	133	140
42	103	109	115	122	129	136	144

^a^GA expressed as completed gestational weeks, e.g. 12 weeks corresponds to 12 + 0 weeks or 84 gestational days.

**Table 7:** Estimated mean abdominal diameter (MAD) in mm by gestational age (GA) for males and females, percentiles.

GA (weeks^a^)	2.5th	5th	10th	25th	Median	75th	90th	95th	97.5th
12	16	16	17	17	18	19	19	20	20
13	20	20	20	21	22	22	23	23	24
14	23	23	24	25	25	26	27	27	28
15	27	27	27	28	29	30	31	31	32
16	30	31	31	32	33	34	35	35	36
17	34	34	35	36	37	38	39	39	40
18	37	38	38	39	41	42	43	43	44
19	41	41	42	43	44	46	47	48	48
20	44	45	46	47	48	50	51	52	52
21	48	48	49	51	52	53	55	56	56
22	51	52	53	54	56	57	59	60	61
23	55	55	56	58	59	61	63	64	65
24	58	59	60	61	63	65	67	68	69
25	61	62	63	65	67	69	71	72	73
26	65	66	67	68	70	72	74	76	77
27	68	69	70	72	74	76	78	79	81
28	71	72	73	75	77	80	82	83	84
29	74	75	76	79	81	83	86	87	88
30	77	78	80	82	84	87	89	91	92
31	80	81	83	85	88	90	93	95	96
32	83	84	86	88	91	94	97	98	100
33	86	87	89	91	94	97	100	102	103
34	89	90	92	94	98	101	104	106	107
35	92	93	95	98	101	104	107	109	111
36	94	96	98	101	104	107	111	113	114
37	97	98	100	103	107	111	114	116	118
38	100	101	103	106	110	114	118	120	122
39	102	104	106	109	113	117	121	123	125
40	105	106	108	112	116	120	124	127	129
41	107	109	111	115	119	123	128	130	132
42	109	111	114	117	122	126	131	133	136

^a^GA expressed as completed gestational weeks, e.g. 12 weeks corresponds to 12 + 0 weeks or 84 gestational days.

Mean and variance equation for MAD in males and females:

$$E\left( {Zi} \right) = { 6}.{71 } + \, \left[ { - {43}.{\text{56GA}}_{{\text{i}}}^{{ - {2}}} } \right] + \left[ { - {12}.{\text{17GA}}_{{\text{i}}}^{{ - 0.{5}}} } \right]$$

$$Var\left( {Zi} \right) = \, 0.0{4 } + \, \left[ {{627}.{\text{53GA}}_{{\text{i}}}^{{ - {4}}} } \right]{ + }\left[ {{9}.0{\text{6GA}}_{{\text{i}}}^{{ - {2}}} } \right] + \left[ { - 0.{5}0{\text{GA}}_{{\text{i}}}^{{ - 0.{5}}} } \right] + \left[ { - {56}.{\text{88GA}}_{{\text{i}}}^{{ - {2}}} {\text{GA}}_{{\text{i}}}^{{ - 0.{5}}} } \right]{ + }\left[ {{1}.{\text{48GA}}_{{\text{i}}}^{{ - {1}}} } \right]$$

Table 8 shows the median and variance for estimated AC by GA in SD, and Table 9 AC by GA in percentiles. Figure 2d shows the raw data with fitted percentiles for estimated AC by GA.

**Table 8:** Estimated abdominal circumference (AC) in mm by gestational age (GA) for males and females, standard deviations (SD).

GA (weeks^a^)	− 3 SD	− 2 SD	− 1 SD	Median	+ 1 SD	+ 2 SD	+ 3 SD
12	48	51	54	57	60	64	67
13	59	62	65	69	72	76	80
14	70	74	77	81	84	88	93
15	81	85	89	93	97	101	106
16	92	96	100	105	110	115	120
17	103	107	112	117	122	128	134
18	114	119	124	129	135	141	147
19	124	130	136	142	148	155	161
20	135	141	147	154	161	168	175
21	146	152	159	166	173	181	189
22	156	163	170	178	186	194	203
23	166	174	181	190	198	207	216
24	176	184	192	201	210	220	230
25	186	195	203	213	222	232	243
26	196	205	214	224	234	245	256
27	205	215	225	235	246	257	269
28	215	225	235	246	258	270	282
29	224	234	245	257	269	282	295
30	233	244	255	268	280	294	308
31	241	253	265	278	292	306	321
32	250	262	275	289	303	318	333
33	258	271	285	299	314	329	346
34	266	280	294	309	324	341	358
35	274	288	303	319	335	352	370
36	282	297	312	328	346	364	383
37	290	305	321	338	356	375	395
38	297	313	330	348	366	386	407
39	304	321	338	357	376	397	419
40	311	329	347	366	386	408	431
41	318	336	355	375	396	419	442
42	325	343	363	384	406	429	454

^a^GA expressed as completed gestational weeks, e.g. 12 weeks corresponds to 12 + 0 weeks or 84 gestational days.

**Table 9:** Estimated abdominal circumference (AC) in mm by gestational age (GA) for males and females, percentiles.

GA (weeks^a^)	2.5th	5th	10th	25th	Median	75th	90th	95th	97.5th
12	51	52	53	55	57	59	61	63	64
13	62	63	65	67	69	71	73	75	76
14	74	75	76	78	81	83	86	87	88
15	85	86	88	90	93	96	98	100	101
16	96	98	99	102	105	108	111	113	114
17	108	109	111	114	117	121	124	126	128
18	119	121	122	126	129	133	137	139	141
19	130	132	134	138	142	146	150	152	154
20	141	143	145	149	154	158	163	165	167
21	152	154	157	161	166	171	175	178	181
22	163	165	168	173	178	183	188	191	194
23	174	176	179	184	190	195	200	204	207
24	184	187	190	195	201	207	213	216	219
25	195	198	201	206	213	219	225	229	232
26	205	208	211	217	224	231	237	241	245
27	215	218	222	228	235	242	249	253	257
28	225	228	232	239	246	254	261	265	269
29	235	238	242	249	257	265	273	277	281
30	244	248	252	259	268	276	284	289	293
31	254	257	262	269	278	287	296	301	305
32	263	267	271	279	289	298	307	312	317
33	272	276	281	289	299	309	318	324	329
34	280	285	290	299	309	319	329	335	340
35	289	293	299	308	319	330	340	346	352
36	297	302	308	317	328	340	351	357	363
37	306	311	316	327	338	350	361	368	374
38	314	319	325	335	348	360	372	379	385
39	321	327	333	344	357	370	382	390	396
40	329	335	342	353	366	380	392	400	407
41	337	343	350	361	375	389	402	411	418
42	344	350	358	370	384	399	412	421	428

^a^GA expressed as completed gestational weeks, e.g. 12 weeks corresponds to 12 + 0 weeks or 84 gestational days.

Mean and variance equation for AC in males and females:

$$E(Z_{{\text{i}}} ) \, = { 7}.{8}0 \, + \, \left[ { - {5}0.0{\text{5 GA}}_{{\text{i}}}^{{ - {2}}} } \right] \, + \, \left[ { - {11}.{8}0{\text{ GA}}_{{\text{i}}}^{{ - 0.{5}}} } \right]$$

$$Var(Z_{{\text{i}}} ) \, = \, 0.0{4 } + \, \left[ {{47}0.{\text{55 GA}}_{{\text{i}}}^{{ - {4}}} } \right] \, + \, \left[ {{6}.{\text{86 GA}}_{{\text{i}}}^{{ - {2}}} } \right] \, + \, \left[ { - 0.{\text{39 GA}}_{{\text{i}}}^{{ - 0.{5}}} } \right] \, + \, \left[ { - {42}.{8}0{\text{ GA}}_{{\text{i}}}^{{ - {2}}} {\text{GA}}_{{\text{i}}}^{{ - 0.{5}}} } \right] \, + \, [{1}.{\text{15 GA}}_{{\text{i}}}^{{ - {1}}}]$$

Table 10 shows the median and variance for estimated FL by GA in SD, and Table 11 FL by GA in percentiles. Figure 2e shows the raw data with fitted percentiles for estimated FL by GA.

**Table 10:** Estimated femur length (FL) in mm by gestational age (GA) for males and females, standard deviations (SD).

GA (weeks^a^)	− 3 SD	− 2 SD	− 1 SD	Median	+ 1 SD	+ 2 SD	+ 3 SD
12	5	5	6	6	7	8	9
13	7	8	8	9	10	11	12
14	10	10	11	12	13	14	15
15	13	14	14	15	16	17	19
16	16	17	18	19	20	21	22
17	19	20	21	22	23	24	26
18	22	23	24	25	27	28	29
19	25	26	27	28	30	31	33
20	28	29	30	32	33	34	36
21	30	32	33	35	36	38	39
22	33	34	36	37	39	41	43
23	35	37	39	40	42	44	46
24	38	39	41	43	45	47	49
25	40	42	44	45	47	49	51
26	42	44	46	48	50	52	54
27	44	46	48	50	52	54	57
28	46	48	50	52	55	57	59
29	48	50	52	54	57	59	62
30	50	52	54	57	59	61	64
31	52	54	56	59	61	64	66
32	53	56	58	60	63	66	69
33	55	57	60	62	65	68	71
34	57	59	62	64	67	70	73
35	58	61	63	66	69	72	75
36	60	62	65	68	71	74	77
37	61	64	66	69	72	75	79
38	63	65	68	71	74	77	81
39	64	67	70	73	76	79	82
40	65	68	71	74	77	81	84
41	67	69	73	76	79	83	86
42	68	71	74	77	81	84	88

^a^GA expressed as completed gestational weeks, e.g. 12 weeks corresponds to 12 + 0 weeks or 84 gestational days.

**Table 11:** Estimated femur length (FL) in mm by gestational age (GA) for males and females, percentiles.

GA (weeks^a^)	2.5th	5th	10th	25th	Median	75th	90th	95th	97.5th
12	5	5	5	6	6	7	7	8	8
13	8	8	8	9	9	10	10	10	11
14	10	11	11	12	12	13	13	14	14
15	14	14	14	15	15	16	17	17	17
16	17	17	17	18	19	19	20	20	21
17	20	20	21	21	22	23	23	24	24
18	23	23	24	24	25	26	27	27	28
19	26	26	27	28	28	29	30	31	31
20	29	29	30	31	32	33	33	34	34
21	32	32	33	34	35	36	37	37	38
22	34	35	35	36	37	39	40	40	41
23	37	38	38	39	40	41	42	43	44
24	39	40	41	42	43	44	45	46	47
25	42	42	43	44	45	47	48	49	49
26	44	45	45	46	48	49	50	51	52
27	46	47	47	49	50	52	53	54	54
28	48	49	50	51	52	54	55	56	57
29	50	51	52	53	54	56	57	58	59
30	52	53	54	55	57	58	60	61	61
31	54	55	55	57	59	60	62	63	64
32	56	56	57	59	60	62	64	65	66
33	57	58	59	61	62	64	66	67	68
34	59	60	61	62	64	66	68	69	70
35	61	62	62	64	66	68	70	71	72
36	62	63	64	66	68	70	71	72	73
37	64	65	66	67	69	71	73	74	75
38	65	66	67	69	71	73	75	76	77
39	67	68	69	71	73	75	77	78	79
40	68	69	70	72	74	76	78	80	81
41	70	71	72	74	76	78	80	81	82
42	71	72	73	75	77	80	82	83	84

^a^GA expressed as completed gestational weeks, e.g. 12 weeks corresponds to 12 + 0 weeks or 84 gestational days.

Mean and variance equation for FL in males and females:

$$E(Z_{{\text{i}}} ) \, = { 4}.{11 } + \, \left[ { - {344}.{\text{24 GA}}_{{\text{i}}}^{{ - {2}}} } \right] \, + \, \left[ {0.0{\text{1 GA}}_{{\text{i}}}^{{1}} } \right]$$

$$Var(Z_{{\text{i}}} ) \, = \, 0.0{1 } + \, \left[ {{584}.{\text{71 GA}}_{{\text{i}}}^{{ - {4}}} } \right] \, + \, \left[ { - {4}.{\text{46 GA}}_{{\text{i}}}^{{ - {2}}} } \right] \, + \, \left[ { - 0.000{\text{4 GA}}_{{\text{i}}}^{{1}} } \right] \, + \, \left[ {0.{\text{10 GA}}_{{\text{i}}}^{{ - {2}}} {\text{GA}}_{{\text{i}}}^{{1}} } \right] \, + \, \left[ {{4}.{\text{83e}} - 0{\text{6 GA}}_{{\text{i}}}^{{2}} } \right]$$

Supplementary Tables 1–5 show the median and variance of the different biometric measurements (BPD, HC, MAD, AC and FL) for each gestational week for males and females separately. Supplementary Tables 6–10 show the median and variance of the different biometric measurements for each gestational day. The variance is expressed in standard deviations (+ 3 SD, + 2 SD, + 1 SD, median, − 1 SD, − 2 SD and − 3 SD) and in percentiles (2.5th, 5th, 10th, 25th, median, 75th, 90th, 95th and 97.5th). Moreover, all supplementary tables enclose the full equations of mean and variance for each biometric measurement.

The sensitivity analysis of women with BMI 18.5 to 29.9 kg/m^2^ showed no statistically significant differences between the reference ranges in the complete study population and the subset of women where underweight and obese women were excluded (p = 0.9906 to 0.999). Supplementary Tables 11–15 show the median and variance of the different biometric measurements for each gestational week in the subset of women with BMI 18.5 to 29.9 kg/m^2^. The variance is expressed in standard deviations (+ 3 SD, + 2 SD, + 1 SD, median, − 1 SD, − 2 SD and − 3 SD) and in percentiles (2.5th, 5th, 10th, 25th, median, 75th, 90th, 95th and 97.5th). Moreover, the supplementary tables enclose the full equations of mean and variance for each biometric measurement.

The GA at examination of the five study subjects included in the reproducibility study varied from 13 + 3 to 41 + 1 gestational weeks. The linear mixed effects model showed overall non-significant inter-observer variation for all five biometric measurements, with p = 0.162 for BPD, p = 0.124 for HC, p = 0.213 for FL, p = 0.087 for MAD and p = 0.166 for AC. The two-way mixed effects model of average measures and absolute agreement showed a very high ICC, with highly consistent measurements for all five biometric measurements; ICC for BPD = 1.000, for HC = 1.000, for FL = 1.000, for MAD = 0.999 and for AC = 0.979.

## Discussion

In this cohort of prospectively enrolled, healthy women with low risk of aberrant fetal growth, we have constructed new Swedish reference ranges for normal size and growth of the fetal head, abdomen and femur. We have provided charts for five biometric measurements; BPD, HC, MAD, AC and FL, from gestational week 12 to 42.

Over the years, a large number of studies have presented regional and international charts for fetal size and growth. There is a large variability in study design and statistical modelling methods, as well as in reported percentiles^3,4,18^. The aim of a fetal growth chart is to describe how fetuses should grow under optimal conditions^3^. Hence, in concordance with large international studies of fetal size and growth, the present study has only included women with low risk of aberrant fetal growth^5,6^.

Reliable and population-representative size and growth charts are important in order to correctly evaluate both fetal size and growth, the latter as serial measurements. Altman and Chitty highlight differences in estimating size and growth, and how this affects the choice of appropriate study design^18^. A cross-sectional design is recommended for evaluating size, with a single measurement on each study subject. Longitudinal studies, on the other hand, comprise repeated measurements of each study subject. Compared with cross-sectional studies, longitudinal studies often use smaller study samples with measurements that are not independent of each other. Unless the repeated measurements are properly addressed, the variation may be underestimated using a longitudinal design. Since the publication of the intrauterine growth charts constructed by Maršál et al. in^12^, which are presently used in Sweden, statistical methods have been developed and used that take both repeated measurements and increased variation with GA into account. These methods permit the use of a longitudinal design to produce growth charts of size as well as growth intended for clinical practice^4,18^. A strength of our study is the prospective longitudinal design and the use of modern statistical modelling methods. Hence, the growth charts can be used to evaluate ultrasonically derived fetal biometry, both regarding size and growth. However, these growth charts are not intended for dating of pregnancies, as dating standards require different statistical analyses^19^. We recommend the use of dating charts that are designed solely for that purpose.

Another strength of our study is the relatively large cohort of healthy women with low-risk pregnancies recruited specifically for this study with an even spread of the examinations across the included GAs. In order to increase the reproducibility and decrease the measurement error, a limited number of experienced sonographers conducted the ultrasound scans following the biometric measurement recommendations that are in use in Sweden. The use of triplicate measurements of each biometric assessment at each ultrasound scan further reduces measurement error. The reproducibility study showed a low grade of inter-observer variability. However, the low number of study subjects is a limitation. Accordingly, the reliability of the reproducibility study cannot be assessed as high. Lastly, strict criteria for exclusion due to increased risk of aberrant growth have been applied throughout the study.

A valid estimation of GA is considered crucial for developing reliable growth reference ranges^3^. The used method with regular menstrual cycles where estimated date of delivery (EDD) according to last menstrual period is consistent with first trimester ultrasound dating provides a reliable dating method^3,4,20–22^. The median discrepancy of 0 days in our study indicates concordant dating between EDD according to BPD and EDD according to last menstrual period. The mean dating discrepancy was larger for girls than for boys. The dating discrepancy is in line with the findings of earlier studies that have examined discrepancy in dating using last menstrual period and ultrasound^23,24^. We used BPD in gestational week 12 + 3 – 13 + 6 to date the pregnancies. Swedish as well as international guidelines recommend dating with ultrasound during the first trimester, as this appears to be the most reliable method for pregnancy dating^14,25^. The Swedish guidelines recommend the use of crown rump length (CRL) in early pregnancy, and BPD from 21 mm (corresponding to week 12 + 3). Adherance to the recommendations of using CRL for dating in early pregnancy is however low in Sweden^26^. Since many Swedish sonographers are not experienced in measuring CRL, we chose to date all pregnancies with BPD in order to avoid different dating methods. Further, the equation used for dating with BPD was derived with CRL as reference for “true” GA, and later the equation was validated as well performing with low systematic and random error^27,28^. However, first trimester dating with BPD predicts the GA and duration of pregnancy equally well as CRL, and the choice of future dating method should therefore not affect the applicability of our growth charts^27,29^. Variations in early growth might have an impact on the estimated GA when first trimester ultrasound dating is used rather than last menstrual period. This implies that there is a risk that a systematic bias caused by measurement error is introduced. The potential effect of inaccurate GA assessment due to natural variation in fetal growth should however be small, as the dating discrepancy was very small.

A limitation of the study is the predominance (92.1%) of women born in Sweden or another Nordic country. This figure is high compared with the Swedish pregnant population, where 69.5% of all women giving birth in Sweden in 2018 were born in a Nordic country^13^. Some selection bias was unavoidable, as the written information to potential study subjects that was handed out during the recruitment process was solely available in Swedish, English and Arabic. Efforts were made to recruit women of various ethnicities and social backgrounds, by recruiting women in primary care units with a high rate of immigrants as well as in units with mainly Swedish born women.

In order to achieve a study population representative to the Swedish pregnant population, women of low as well as high BMI were included in the cohort, despite the potential effect of abnormal BMI on intrauterine growth. Since only healthy women were included, the risk of poor intrauterine growth due to malnutrition should be low. Even though the median BMI was normal, the upper interquartile range included women with overweight, indicating that a significant part of the study population were overweight. Women with obesity were not only screened with repeated random plasma glucose, but also with oral glucose tolerance test for gestational diabetes. All women who developed gestational diabetes were excluded, and solely women with normal plasma glucose and glucose tolerance fulfilled the study. It cannot be ruled out that increased fetal growth due to other factors than gestational diabetes in obese women might affect the results towards an overestimation of normal fetal size. In the sensitivity analysis, where women with BMI 18.5 to 29.9 kg/m^2^ were compared with the complete study population, only small differences were observed between the groups for all biometric measurements. These differences should not be of any clinical significance, and neither were there any statistically significant differences between the reference ranges if all women were included or not. Hence, including the subjects with extreme BMI values should not bias the results. Moreover, the aim of the study was to provide reference ranges in a study population of healthy women representative to the Swedish pregnant population. Maternal age and BMI in the study population were similar to the mean age (30.4 years) and BMI (25.2 kg/m^2^) of pregnant women in Sweden 2017^13^. Hence, the results of the complete study population can be regarded as generalizable for estimation of fetal size and growth in the current Swedish pregnant population.

Compared with the growth charts presently used in Sweden, our new reference ranges are derived from an almost seven times larger study population^12^. Moreover, the study population in Maršál’s study comprises 24% smokers. Considering the potential growth retarding effect of maternal smoking, their study population does not represent a low-risk population with expected normal fetal growth^30,31^. Methodological considerations, such as the nowadays outdated cross-sectional analytic methods of a longitudinal study in the Maršál study, and changes in the Swedish pregnant population, motivates a change into updated reference ranges for fetal size and growth. Moreover, the corresponding Norwegian growth charts, which are based on a methodology similar to ours, are not entirely applicable to the Swedish setting, partly due to differences in demographics and birthweights, but most importantly due to differences in recommendations for how to perform the ultrasonic BPD measurements. The Norwegian reference ranges for biometry are calculated using the calipers placed on the outer margins of both the proximal and the distal parietal bone^16^.

During the last few years, large international projects have produced growth standards intended for universal use, with the assumption that differences in fetal growth and birthweights are caused by suboptimal environment rather than inherent differences in the populations^5,6,32^. Others have found evidence supporting that physiological differences rather than pathology explain the differences in size and growth between populations^8,33–35^. Applying international standards would in such a case possibly misclassify a large proportion of fetuses as either SGA, AGA or LGA^10^. There is an ongoing debate concerning the need of national standards for fetal size and growth. It is interesting to note that even though the INTERGROWTH-21st project showed high degree of likeness between study sites, the WHO Multicentre Growth Reference Study reported significant differences in fetal growth in different settings^5,6^. Even though both studies are of high quality with large study populations, recent studies have presented evidence that questions the use of a single international standard that represents ideal growth in all populations^8–10,35^. Similar conclusions were drawn by the authors of the WHO study, who recommend that if international charts are used, their performance should be tested in the local setting to assess if adjustments are needed^5^. Bearing these concerns in mind, we believe that there is a need for updated national reference ranges of fetal size and growth for everyday clinical practice. Moreover, further studies are needed to evaluate proper cut-offs for the updated reference ranges in order to identify fetuses with increased risk of adverse perinatal outcome.

In conclusion, as regional differences in fetal size and growth seem to be of clinical importance, this prospective longitudinal study of normal fetal growth in a healthy Swedish population provides new national reference ranges for fetal size and growth from gestational week 12 to 42.

### Research involving human participants

The study was approved by the Regional Ethical Review Board in Uppsala (no. 2014/209 and 2014/209/2). All procedures involving human subjects were carried out in accordance with the ethical standards of the 1964 Helsinki declaration. All women participated voluntarily and gave their informed consent. Any pregnancy complication recognized during the study was reported to the routine obstetric care at each study site and managed according to clinical practice.

## Supplementary Information

Supplementary Table 1.

Supplementary Table 2.

Supplementary Table 3.

Supplementary Table 4.

Supplementary Table 5.

Supplementary Table 6.

Supplementary Table 7.

Supplementary Table 8.

Supplementary Table 9.

Supplementary Table 10.

Supplementary Table 11.

Supplementary Table 12.

Supplementary Table 13.

Supplementary Table 14.

Supplementary Table 15.

## Data Availability

The datasets generated during and/or analysed during the current study are not publicly available due to the ethical and legal restrictions prohibiting the sharing of personal data, but are available from the corresponding author on reasonable request.
