# Author Correction: Swedish intrauterine growth reference ranges of biometric measurements of fetal head, abdomen and femur

**DOI:** 10.1038/s41598-021-98263-7

**Published:** 2021-09-10

**Authors:** Linda Lindström, Mårten Ageheim, Ove Axelsson, Laith Hussain-Alkhateeb, Alkistis Skalkidou, Eva Bergman

**Affiliations:** 1 Department of Women’s and Children’s Health, Uppsala University, Uppsala, Sweden; 2 Centre for Clinical Research Sörmland, Uppsala University, Eskilstuna, Sweden; 3 Global Health, School of Public Health and Community Medicine, Institute of Medicine, Sahlgrenska Academy, University of Gothenburg, Gothenburg, Sweden

Correction to: *Scientific Reports* https://doi.org/10.1038/s41598-020-79797-8, published online 31 December 2020

The original version of this Article contained errors in the Equations.

In the Results section,

Mean and variance equation for BPD in males and females:

$$ E\left( {Z_{i} } \right) = - {2}.{53 } + \left[ {{2}.{\text{47 log}}\left( {{\text{GA}}_{{\text{i}}} } \right)} \right] + \left[ { - 0.0{\text{5 GA}}_{{\text{i}}}{^{{1}}} } \right] $$

$$ Var\left( {Zi} \right) = 0.0{8 } \, + \left[ {0.0{\text{2 log}}\left( {{\text{GA}}_{{\text{i}}} } \right)^{{2}} } \right] + \left[ { - 0.0{\text{8 log}}\left( {{\text{GA}}_{{\text{i}}} } \right)} \right] + \left[ {0.00{\text{3 GA}}_{{\text{i}}}{^{{1}}} } \right] + \left[ { - 0.000{\text{8 log}}\left( {{\text{GA}}_{{\text{i}}} } \right){\text{GA}}_{{\text{i}}}{^{{1}}} } \right] + \left[ {0.0000{\text{4 GA}}_{{\text{i}}}{^{{2}}} } \right] $$

Mean and variance equation for HC in males and females:

$$ E\left( {Zi} \right) = { 8}.{48 }  \, + \left[ { - {14}.{\text{36 GA}}_{{\text{i}}}{^{{ - 0.{5}}}} } \right] + \left[ { - 0.000{\text{2 GA}}_{{\text{i}}}{^{{2}}} } \right] $$

$$ Var\left( {Zi} \right) = 0.0{2 }  \, + \left[ {0.{\text{25 GA}}_{{\text{i}}}{^{{ - {1}}}} } \right] + \left[ { - 0.{\text{13 GA}}_{{\text{i}}}{^{{ - 0.{5}}}} } \right] + \left[ { - {8}.{\text{24e}}{ -} 0{\text{6 GA}}_{{\text{i}}}{^{{2}}} } \right] + \left[ {0.0000{\text{1 GA}}_{{\text{i}}}{^{{ - 0.{5}}}} {\text{GA}}_{{\text{i}}}{^{{2}}} } \right] + \left[ {{1}.{\text{43e}}{-} 0{\text{9 GA}}_{{\text{i}}}{^{{4}}} } \right] $$

Mean and variance equation for MAD in males and females:

$$ E\left( {Zi} \right) = { 6}.{71 }  \, + [ - {43}.{\text{56GA}}_{{\text{i}}}{^{{ - {2}}}} \left] + \right[ - {12}.{\text{17GA}}_{{\text{i}}}{^{{ - 0.{5}}}} ] $$

$$ \begin{aligned}  Var\left( {Zi} \right) &= 0.0{4}  \, + \left[{627}.{\text{53GA}}_{{\text{i}}}{^{{ - {4}}}} \right] + \left[{9}.0{\text{6GA}}_{{\text{i}}}{^{{ - {2}}}} \right] + \left[ - 0.{5}0{\text{GA}}_{{\text{i}}}{^{{ - 0.{5}}}} \right] \\ &\quad + \left[ - {28}.{\text{44GA}}_{{\text{i}}}{^{{ - {2}}}} {\text{GA}}_{{\text{i}}}{^{{ - 0.{5}}}} \right] + \left[{1}.{\text{48GA}}_{{\text{i}}}{^{{ - {1}}}} \right] \end{aligned}$$

Mean and variance equation for AC in males and females:

$$ E(Z_{{\text{i}}} ) \, = { 7}.{8}0  \, + \left[ { - {5}0.0{\text{5 GA}}_{{\text{i}}}{^{{ - {2}}}} } \right] + \left[ { - {11}.{8}0{\text{ GA}}_{{\text{i}}}{^{{ - 0.{5}}}} } \right] $$

$$ Var(Z_{{\text{i}}} ) \, = 0.0{4 }  \, + \left[ {{47}0.{\text{55 GA}}_{{\text{i}}}{^{{ - {4}}}} } \right] + \left[ {{6}.{\text{86 GA}}_{{\text{i}}}{^{{ - {2}}}} } \right] + \left[ { - 0.{\text{39 GA}}_{{\text{i}}}{^{{ - 0.{5}}}} } \right] + \left[ { - {21}.{4}0{\text{ GA}}_{{\text{i}}}{^{{ - {2}}}} {\text{GA}}_{{\text{i}}}{^{{ - 0.{5}}}} } \right] + \left[ {{1}.{\text{15 GA}}_{{\text{i}}}{^{{ - {1}}}} } \right] $$

Mean and variance equation for FL in males and females

$$ E(Z_{{\text{i}}} ) \, = { 4}.{11 }  \, + \left[ { - {344}.{\text{24 GA}}_{{\text{i}}}{^{{ - {2}}}} } \right] + \left[ {0.0{\text{1 GA}}_{{\text{i}}}{^{{1}}} } \right] $$

$$ Var(Z_{{\text{i}}} ) \, = 0.0{1 }  \, + \left[ {{584}.{\text{71 GA}}_{{\text{i}}}{^{{ - {4}}}} } \right] + \left[ { - {4}.{\text{46 GA}}_{{\text{i}}}{^{{ - {2}}}} } \right] + \left[ { - 0.000{\text{4 GA}}_{{\text{i}}}{^{{1}}} } \right] + \left[ {0.0{\text{5 GA}}_{{\text{i}}}{^{{ - {2}}}} {\text{GA}}_{{\text{i}}}{^{{1}}} } \right] + \left[ {{4}.{\text{83e}}{ - }0{\text{6 GA}}_{{\text{i}}}{^{{2}} }} \right] $$

now reads:

Mean and variance equation for BPD in males and females:

$$ E\left( {Z_{i} } \right) = - {2}.{53 }  \, + \left[ {{2}.{\text{47 log}}\left( {{\text{GA}}_{{\text{i}}} } \right)} \right] + \left[ { - 0.0{\text{5 GA}}_{{\text{i}}}{^{{1}}} } \right] $$

$$ Var\left( {Zi} \right) = 0.0{8 }  \, + \left[ {0.0{\text{2 log}}\left( {{\text{GA}}_{{\text{i}}} } \right){^{{2}}} } \right] + \left[ { - 0.0{\text{8 log}}\left( {{\text{GA}}_{{\text{i}}} } \right)} \right] + \left[ {0.00{\text{3 GA}}_{{\text{i}}}{^{{1}}} } \right] + \left[ { - 0.000{\text{2 log}}\left( {{\text{GA}}_{{\text{i}}} } \right){\text{GA}}_{{\text{i}}}{^{{1}}} } \right] + \left[ {0.0000{\text{4 GA}}_{{\text{i}}}{^{{2}}} } \right] $$

Mean and variance equation for HC in males and females:

$$ E\left( {Zi} \right) = { 8}.{48 }  \, + \left[ { - {14}.{\text{36 GA}}_{{\text{i}}}{^{{ - 0.{5}}}} } \right] + \left[ { - 0.000{\text{2 GA}}_{{\text{i}}}{^{{2}}} } \right] $$

$$ Var\left( {Zi} \right) = 0.0{2 }  \, + \left[ {0.{\text{25 GA}}_{{\text{i}}}{^{{ - {1}}}} } \right] + \left[ { - 0.{\text{13 GA}}_{{\text{i}}}{^{{ - 0.{5}}}} } \right] + \left[ { - {8}.{\text{24e}}{ -} 0{\text{6 GA}}_{{\text{i}}}{^{{2}}} } \right] + \left[ {0.0000{\text{3 GA}}_{{\text{i}}}{^{{ - 0.{5}}}} {\text{GA}}_{{\text{i}}}{^{{2}}} } \right] + \left[ {{1}.{\text{43e}} {-} 0{\text{9 GA}}_{{\text{i}}}{^{{4}}} } \right] $$

Mean and variance equation for MAD in males and females:

$$ E\left( {Zi} \right) = { 6}.{71 }  \, + [ - {43}.{\text{56GA}}_{{\text{i}}}{^{{ - {2}}}} \left] + \right[ - {12}.{\text{17GA}}_{{\text{i}}}{^{{ - 0.{5}}}} ] $$

$$ \begin{aligned}  Var\left( {Zi} \right) & = 0.0{4 } \, + \left[{627}.{\text{53GA}}_{{\text{i}}}{^{{ - {4}}}} \right] + \left[{9}.0{\text{6GA}}_{{\text{i}}}{^{{ - {2}}}} \right] + \left[ - 0.{5}0{\text{GA}}_{{\text{i}}}{^{{ - 0.{5}}}} \right] \\ &\quad + \left[ - {56}.{\text{88GA}}_{{\text{i}}}{^{{ - {2}}}} {\text{GA}}_{{\text{i}}}{^{{ - 0.{5}}}} \right] + \left[{1}.{\text{48GA}}_{{\text{i}}}{^{{ - {1}}}} \right] \end{aligned} $$

Mean and variance equation for AC in males and females:

$$ E(Z_{{\text{i}}} ) \, = { 7}.{8}0  \, + \left[ { - {5}0.0{\text{5 GA}}_{{\text{i}}}{^{{ - {2}}}} } \right] + \left[ { - {11}.{8}0{\text{ GA}}_{{\text{i}}}{^{{ - 0.{5}}}} } \right] $$

$$ Var(Z_{{\text{i}}} ) \, = 0.0{4 } \, + \left[ {{47}0.{\text{55 GA}}_{{\text{i}}}{^{{ - {4}}}} } \right] + \left[ {{6}.{\text{86 GA}}_{{\text{i}}}{^{{ - {2}}}} } \right] + \left[ { - 0.{\text{39 GA}}_{{\text{i}}}{^{{ - 0.{5}}}} } \right] + \left[ { - {42}.{8}0{\text{ GA}}_{{\text{i}}}{^{{ - {2}}}} {\text{GA}}_{{\text{i}}}{^{{ - 0.{5}}}} } \right] + \left[ {{1}.{\text{15 GA}}_{{\text{i}}}{^{{ - {1}}}} } \right] $$

Mean and variance equation for FL in males and females

$$ E(Z_{{\text{i}}} ) = {4}.{11}  \, + \left[ { - {344}.{\text{24 GA}}_{{\text{i}}}{^{{ - {2}}}} } \right] + \left[ {0.0{\text{1 GA}}_{{\text{i}}}{^{{1}}} } \right] $$

$$ Var(Z_{{\text{i}}} ) \, = 0.0{1 }  \, + \left[ {{584}.{\text{71 GA}}_{{\text{i}}}{^{{ - {4}}}} } \right] + \left[ { - {4}.{\text{46 GA}}_{{\text{i}}}{^{{ - {2}}}} } \right] + \left[ { - 0.000{\text{4 GA}}_{{\text{i}}}{^{{1}}} } \right] + \left[ {0.{1}0{\text{ GA}}_{{\text{i}}}{^{{ - {2}}}} {\text{GA}}_{{\text{i}}}{^{{1}}} } \right] + \left[ {{4}.{\text{83e}}{ -} 0{\text{6 GA}}_{{\text{i}}}{^{{2}}} } \right] $$

The original Article has been corrected.

