# Supplementary material for: Swedish intrauterine growth reference ranges of biometric measurements of fetal head, abdomen and femur
Source: Sci Rep. 2020 Dec 31;10:22441. doi: 10.1038/s41598-020-79797-8 (PMC7775468; doi:10.1038/s41598-020-79797-8)
Supplement: Supplementary file 1 — Supplementary Table 1. [file 41598_2020_79797_MOESM1_ESM.docx]

Supplementary Table 1a. Estimated biparietal diameter (BPD) in mm by gestational age (GA) for males, Standard deviations (SD).

| GA (weeks*) | -3 SD | -2 SD | -1 SD | Median | +1 SD | +2 SD | +3 SD |
| --- | --- | --- | --- | --- | --- | --- | --- |
| 12 | 19 | 19 | 20 | 20 | 21 | 21 | 22 |
| 13 | 22 | 22 | 23 | 23 | 24 | 24 | 25 |
| 14 | 25 | 26 | 26 | 27 | 27 | 28 | 29 |
| 15 | 28 | 29 | 29 | 30 | 31 | 32 | 33 |
| 16 | 31 | 32 | 33 | 34 | 35 | 36 | 36 |
| 17 | 34 | 35 | 36 | 37 | 38 | 39 | 41 |
| 18 | 37 | 38 | 40 | 41 | 42 | 43 | 45 |
| 19 | 40 | 42 | 43 | 44 | 46 | 47 | 49 |
| 20 | 43 | 45 | 46 | 48 | 49 | 51 | 53 |
| 21 | 46 | 48 | 50 | 51 | 53 | 55 | 57 |
| 22 | 49 | 51 | 53 | 55 | 57 | 59 | 61 |
| 23 | 52 | 54 | 56 | 58 | 60 | 62 | 65 |
| 24 | 55 | 57 | 59 | 61 | 64 | 66 | 68 |
| 25 | 58 | 60 | 62 | 64 | 67 | 69 | 72 |
| 26 | 60 | 63 | 65 | 67 | 70 | 73 | 75 |
| 27 | 63 | 65 | 68 | 70 | 73 | 76 | 79 |
| 28 | 65 | 68 | 71 | 73 | 76 | 79 | 82 |
| 29 | 68 | 70 | 73 | 76 | 79 | 82 | 85 |
| 30 | 70 | 73 | 76 | 78 | 81 | 84 | 88 |
| 31 | 72 | 75 | 78 | 81 | 84 | 87 | 90 |
| 32 | 74 | 77 | 80 | 83 | 86 | 89 | 93 |
| 33 | 76 | 79 | 82 | 85 | 88 | 92 | 95 |
| 34 | 78 | 81 | 84 | 87 | 90 | 94 | 97 |
| 35 | 80 | 83 | 86 | 89 | 92 | 96 | 99 |
| 36 | 81 | 84 | 87 | 91 | 94 | 98 | 101 |
| 37 | 82 | 85 | 89 | 92 | 96 | 99 | 103 |
| 38 | 83 | 87 | 90 | 93 | 97 | 101 | 105 |
| 39 | 84 | 88 | 91 | 95 | 98 | 102 | 106 |
| 40 | 85 | 88 | 92 | 96 | 100 | 104 | 108 |
| 41 | 86 | 89 | 93 | 97 | 101 | 105 | 109 |
| 42 | 86 | 90 | 93 | 97 | 102 | 106 | 111 |

*GA expressed as completed gestational weeks, e.g. 12 weeks corresponds to 12+0 weeks or 84 gestational days.

Mean and variance equation for BPD in males:

*E(Z*_i_) = -2.591884812384639 + [2.503516057616504 log(GA_i_)] + [-0.052038787466868 GA_i_^1^]

*Var(Z*_i_) = 0.0944936664603718 + [0.0232196248474312 log(GA_i_)^2^] + [-0.0932759691015798 log(GA_i_)] + [0.0038418991017616 GA_i_^1^] + [-0.0009678178212483 log(GA_i_)GA_i_^1^] + [0.0000419704469151 GA_i_^2^]

Supplementary Table 1b. Estimated biparietal diameter (BPD) in mm by gestational age (GA) for males, percentiles.

| GA (weeks*) | 2.5^th^ | 5^th^ | 10^th^ | 25^th^ | Median | 75^th^ | 90^th^ | 95^th^ | 97.5^th^ |
| --- | --- | --- | --- | --- | --- | --- | --- | --- | --- |
| 12 | 19 | 19 | 20 | 20 | 20 | 20 | 21 | 21 | 21 |
| 13 | 22 | 23 | 23 | 23 | 23 | 24 | 24 | 24 | 24 |
| 14 | 26 | 26 | 26 | 26 | 27 | 27 | 28 | 28 | 28 |
| 15 | 29 | 29 | 29 | 30 | 30 | 31 | 31 | 31 | 32 |
| 16 | 32 | 32 | 33 | 33 | 34 | 34 | 35 | 35 | 35 |
| 17 | 35 | 35 | 36 | 36 | 37 | 38 | 39 | 39 | 39 |
| 18 | 38 | 39 | 39 | 40 | 41 | 42 | 42 | 43 | 43 |
| 19 | 42 | 42 | 43 | 43 | 44 | 45 | 46 | 47 | 47 |
| 20 | 45 | 45 | 46 | 47 | 48 | 49 | 50 | 50 | 51 |
| 21 | 48 | 48 | 49 | 50 | 51 | 52 | 54 | 54 | 55 |
| 22 | 51 | 52 | 52 | 53 | 55 | 56 | 57 | 58 | 59 |
| 23 | 54 | 55 | 55 | 57 | 58 | 59 | 61 | 62 | 62 |
| 24 | 57 | 58 | 58 | 60 | 61 | 63 | 64 | 65 | 66 |
| 25 | 60 | 61 | 61 | 63 | 64 | 66 | 68 | 68 | 69 |
| 26 | 63 | 63 | 64 | 66 | 67 | 69 | 71 | 72 | 73 |
| 27 | 65 | 66 | 67 | 69 | 70 | 72 | 74 | 75 | 76 |
| 28 | 68 | 69 | 70 | 71 | 73 | 75 | 77 | 78 | 79 |
| 29 | 71 | 71 | 72 | 74 | 76 | 78 | 80 | 81 | 82 |
| 30 | 73 | 74 | 75 | 76 | 78 | 80 | 82 | 83 | 84 |
| 31 | 75 | 76 | 77 | 79 | 81 | 83 | 85 | 86 | 87 |
| 32 | 77 | 78 | 79 | 81 | 83 | 85 | 87 | 88 | 89 |
| 33 | 79 | 80 | 81 | 83 | 85 | 87 | 89 | 90 | 92 |
| 34 | 81 | 82 | 83 | 85 | 87 | 89 | 91 | 93 | 94 |
| 35 | 83 | 84 | 85 | 87 | 89 | 91 | 93 | 95 | 96 |
| 36 | 84 | 85 | 86 | 88 | 91 | 93 | 95 | 96 | 97 |
| 37 | 85 | 87 | 88 | 90 | 92 | 94 | 97 | 98 | 99 |
| 38 | 87 | 88 | 89 | 91 | 93 | 96 | 98 | 100 | 101 |
| 39 | 88 | 89 | 90 | 92 | 95 | 97 | 100 | 101 | 102 |
| 40 | 89 | 90 | 91 | 93 | 96 | 98 | 101 | 102 | 104 |
| 41 | 89 | 90 | 92 | 94 | 97 | 99 | 102 | 103 | 105 |
| 42 | 90 | 91 | 92 | 95 | 97 | 100 | 103 | 104 | 106 |

*GA expressed as completed gestational weeks, e.g. 12 weeks corresponds to 12+0 weeks or 84 gestational days.

Mean and variance equation for BPD in males:

*E(Z*_i_) = -2.591884812384639 + [2.503516057616504 log(GA_i_)] + [-0.052038787466868 GA_i_^1^]

*Var(Z*_i_) = 0.0944936664603718 + [0.0232196248474312 log(GA_i_)^2^] + [-0.0932759691015798 log(GA_i_)] + [0.0038418991017616 GA_i_^1^] + [-0.0009678178212483 log(GA_i_)GA_i_^1^] + [0.0000419704469151 GA_i_^2^]

Supplementary Table 1c. Estimated biparietal diameter (BPD) in mm by gestational age (GA) for females, Standard deviations (SD).

| GA (weeks*) | -3 SD | -2 SD | -1 SD | Median | +1 SD | +2 SD | +3 SD |
| --- | --- | --- | --- | --- | --- | --- | --- |
| 12 | 19 | 19 | 20 | 20 | 21 | 21 | 22 |
| 13 | 22 | 22 | 23 | 23 | 24 | 25 | 25 |
| 14 | 25 | 25 | 26 | 27 | 27 | 28 | 29 |
| 15 | 28 | 28 | 29 | 30 | 31 | 32 | 32 |
| 16 | 31 | 32 | 33 | 33 | 34 | 35 | 36 |
| 17 | 34 | 35 | 36 | 37 | 38 | 39 | 40 |
| 18 | 37 | 38 | 39 | 40 | 42 | 43 | 44 |
| 19 | 40 | 41 | 42 | 44 | 45 | 47 | 48 |
| 20 | 43 | 44 | 46 | 47 | 49 | 50 | 52 |
| 21 | 46 | 47 | 49 | 51 | 52 | 54 | 56 |
| 22 | 49 | 50 | 52 | 54 | 56 | 58 | 60 |
| 23 | 52 | 53 | 55 | 57 | 59 | 61 | 63 |
| 24 | 54 | 56 | 58 | 60 | 62 | 65 | 67 |
| 25 | 57 | 59 | 61 | 63 | 66 | 68 | 70 |
| 26 | 60 | 62 | 64 | 66 | 69 | 71 | 74 |
| 27 | 62 | 65 | 67 | 69 | 72 | 74 | 77 |
| 28 | 65 | 67 | 70 | 72 | 75 | 77 | 80 |
| 29 | 67 | 70 | 72 | 75 | 77 | 80 | 83 |
| 30 | 70 | 72 | 75 | 77 | 80 | 83 | 86 |
| 31 | 72 | 74 | 77 | 80 | 82 | 85 | 88 |
| 32 | 74 | 76 | 79 | 82 | 85 | 88 | 91 |
| 33 | 76 | 78 | 81 | 84 | 87 | 90 | 93 |
| 34 | 77 | 80 | 83 | 86 | 89 | 92 | 95 |
| 35 | 79 | 82 | 85 | 88 | 91 | 94 | 97 |
| 36 | 81 | 83 | 86 | 89 | 92 | 96 | 99 |
| 37 | 82 | 85 | 88 | 91 | 94 | 97 | 101 |
| 38 | 83 | 86 | 89 | 92 | 96 | 99 | 102 |
| 39 | 84 | 87 | 90 | 94 | 97 | 100 | 104 |
| 40 | 85 | 88 | 91 | 95 | 98 | 102 | 105 |
| 41 | 86 | 89 | 92 | 96 | 99 | 103 | 107 |
| 42 | 87 | 90 | 93 | 97 | 100 | 104 | 108 |

*GA expressed as completed gestational weeks, e.g. 12 weeks corresponds to 12+0 weeks or 84 gestational days.

Mean and variance equation for BPD in females:

*E(Z*_i_) = -2.450814939007116 + [2.435329885389612 log(GA_i_)] + [-0.0495436064895566 GA_i_^1^]

*Var(Z*_i_) = 0.0559616466706161 + [0.0138585955586667 log(GA_i_)^2^] + [-0.0553154925098876 log(GA_i_)] + [0.0022490174928992 GA_i_^1^] + [-0.0005710037621161 log(GA_i_)GA_i_^1^] + [0.0000245750605701 GA_i_^2^]

Supplementary Table 1d. Estimated biparietal diameter (BPD) in mm by gestational age (GA) for females, percentiles.

| GA (weeks*) | 2.5^th^ | 5^th^ | 10^th^ | 25^th^ | Median | 75^th^ | 90^th^ | 95^th^ | 97.5^th^ |
| --- | --- | --- | --- | --- | --- | --- | --- | --- | --- |
| 12 | 19 | 19 | 20 | 20 | 20 | 21 | 21 | 21 | 21 |
| 13 | 22 | 22 | 23 | 23 | 23 | 24 | 24 | 24 | 24 |
| 14 | 25 | 26 | 26 | 26 | 27 | 27 | 28 | 28 | 28 |
| 15 | 29 | 29 | 29 | 29 | 30 | 31 | 31 | 31 | 32 |
| 16 | 32 | 32 | 32 | 33 | 33 | 34 | 35 | 35 | 35 |
| 17 | 35 | 35 | 36 | 36 | 37 | 38 | 38 | 39 | 39 |
| 18 | 38 | 38 | 39 | 40 | 40 | 41 | 42 | 42 | 43 |
| 19 | 41 | 42 | 42 | 43 | 44 | 45 | 46 | 46 | 46 |
| 20 | 44 | 45 | 45 | 46 | 47 | 48 | 49 | 50 | 50 |
| 21 | 47 | 48 | 48 | 49 | 51 | 52 | 53 | 53 | 54 |
| 22 | 50 | 51 | 52 | 53 | 54 | 55 | 56 | 57 | 58 |
| 23 | 53 | 54 | 55 | 56 | 57 | 58 | 60 | 60 | 61 |
| 24 | 56 | 57 | 58 | 59 | 60 | 62 | 63 | 64 | 64 |
| 25 | 59 | 60 | 61 | 62 | 63 | 65 | 66 | 67 | 68 |
| 26 | 62 | 63 | 64 | 65 | 66 | 68 | 69 | 70 | 71 |
| 27 | 65 | 65 | 66 | 68 | 69 | 71 | 72 | 73 | 74 |
| 28 | 67 | 68 | 69 | 70 | 72 | 74 | 75 | 76 | 77 |
| 29 | 70 | 71 | 71 | 73 | 75 | 76 | 78 | 79 | 80 |
| 30 | 72 | 73 | 74 | 75 | 77 | 79 | 81 | 82 | 83 |
| 31 | 74 | 75 | 76 | 78 | 80 | 81 | 83 | 84 | 85 |
| 32 | 76 | 77 | 78 | 80 | 82 | 84 | 85 | 87 | 88 |
| 33 | 78 | 79 | 80 | 82 | 84 | 86 | 88 | 89 | 90 |
| 34 | 80 | 81 | 82 | 84 | 86 | 88 | 90 | 91 | 92 |
| 35 | 82 | 83 | 84 | 86 | 88 | 90 | 92 | 93 | 94 |
| 36 | 84 | 84 | 85 | 87 | 89 | 91 | 93 | 95 | 96 |
| 37 | 85 | 86 | 87 | 89 | 91 | 93 | 95 | 96 | 97 |
| 38 | 86 | 87 | 88 | 90 | 92 | 95 | 97 | 98 | 99 |
| 39 | 87 | 88 | 89 | 91 | 94 | 96 | 98 | 99 | 100 |
| 40 | 88 | 89 | 91 | 92 | 95 | 97 | 99 | 100 | 102 |
| 41 | 89 | 90 | 91 | 93 | 96 | 98 | 100 | 102 | 103 |
| 42 | 90 | 91 | 92 | 94 | 97 | 99 | 101 | 103 | 104 |

*GA expressed as completed gestational weeks, e.g. 12 weeks corresponds to 12+0 weeks or 84 gestational days.

Mean and variance equation for BPD in females:

*E(Z*_i_) = -2.450814939007116 + [2.435329885389612 log(GA_i_)] + [-0.0495436064895566 GA_i_^1^]

*Var(Z*_i_) = 0.0559616466706161 + [0.0138585955586667 log(GA_i_)^2^] + [-0.0553154925098876 log(GA_i_)] + [0.0022490174928992 GA_i_^1^] + [-0.0005710037621161 log(GA_i_)GA_i_^1^] + [0.0000245750605701 GA_i_^2^]
