# Supplementary material for: Swedish intrauterine growth reference ranges of biometric measurements of fetal head, abdomen and femur
Source: Sci Rep. 2020 Dec 31;10:22441. doi: 10.1038/s41598-020-79797-8 (PMC7775468; doi:10.1038/s41598-020-79797-8)
Supplement: Supplementary file 2 — Supplementary Table 2. [file 41598_2020_79797_MOESM2_ESM.docx]

Supplementary Table 2a. Estimated head circumference (HC) in mm by gestational age (GA) for males, Standard deviations (SD).

| GA (weeks*) | -3 SD | -2 SD | -1 SD | Median | +1 SD | +2 SD | +3 SD |
| --- | --- | --- | --- | --- | --- | --- | --- |
| 12 | 65 | 68 | 71 | 73 | 76 | 80 | 83 |
| 13 | 77 | 80 | 83 | 86 | 89 | 93 | 96 |
| 14 | 90 | 93 | 96 | 99 | 102 | 106 | 109 |
| 15 | 102 | 105 | 109 | 112 | 116 | 119 | 123 |
| 16 | 114 | 118 | 122 | 125 | 129 | 133 | 137 |
| 17 | 127 | 131 | 134 | 138 | 143 | 147 | 151 |
| 18 | 139 | 143 | 147 | 152 | 156 | 161 | 165 |
| 19 | 151 | 155 | 160 | 165 | 169 | 174 | 179 |
| 20 | 163 | 167 | 172 | 177 | 182 | 188 | 193 |
| 21 | 174 | 179 | 184 | 190 | 195 | 201 | 207 |
| 22 | 185 | 191 | 196 | 202 | 208 | 214 | 220 |
| 23 | 196 | 202 | 208 | 214 | 220 | 227 | 234 |
| 24 | 206 | 212 | 219 | 225 | 232 | 239 | 246 |
| 25 | 216 | 223 | 229 | 236 | 244 | 251 | 259 |
| 26 | 226 | 232 | 240 | 247 | 254 | 262 | 270 |
| 27 | 235 | 242 | 249 | 257 | 265 | 273 | 282 |
| 28 | 243 | 251 | 259 | 267 | 275 | 284 | 292 |
| 29 | 251 | 259 | 267 | 276 | 285 | 294 | 303 |
| 30 | 259 | 267 | 276 | 285 | 294 | 303 | 313 |
| 31 | 266 | 275 | 284 | 293 | 302 | 312 | 322 |
| 32 | 273 | 282 | 291 | 300 | 310 | 320 | 331 |
| 33 | 279 | 288 | 298 | 307 | 318 | 328 | 339 |
| 34 | 284 | 294 | 304 | 314 | 324 | 335 | 347 |
| 35 | 289 | 299 | 309 | 320 | 331 | 342 | 354 |
| 36 | 293 | 304 | 314 | 325 | 337 | 349 | 361 |
| 37 | 297 | 308 | 319 | 330 | 342 | 355 | 368 |
| 38 | 300 | 311 | 323 | 335 | 347 | 360 | 374 |
| 39 | 302 | 314 | 326 | 339 | 352 | 365 | 380 |
| 40 | 304 | 316 | 329 | 342 | 356 | 370 | 385 |
| 41 | 305 | 318 | 331 | 345 | 359 | 375 | 390 |
| 42 | 305 | 319 | 333 | 347 | 363 | 378 | 395 |

*GA expressed as completed gestational weeks, e.g. 12 weeks corresponds to 12+0 weeks or 84 gestational days.

Mean and variance equation for HC in males:

*E(Z*_i_) = 8.51636027823433 + [-14.49558997497378 GA_i_^-0.5^] + [-0.0002432991293945 GA_i_^2^]

*Var(Z*_i_) = 0.0248425670066804 + [0.3660305465083908 GA_i_^-1^] + [-0.1866960903960692 GA_i_^-0.5^] + [-0.0000123263318793 GA_i_^2^] + [0.000022532442754 GA_i_^-0.5^GA_i_^2^] + [2.10904660584e-09 GA_i_^4^]

Supplementary Table 2b. Estimated head circumference (HC) in mm by gestational age (GA) for males, percentiles.

| GA (weeks*) | 2.5^th^ | 5^th^ | 10^th^ | 25^th^ | Median | 75^th^ | 90^th^ | 95^th^ | 97.5^th^ |
| --- | --- | --- | --- | --- | --- | --- | --- | --- | --- |
| 12 | 68 | 69 | 70 | 72 | 73 | 75 | 77 | 78 | 79 |
| 13 | 80 | 81 | 82 | 84 | 86 | 88 | 90 | 91 | 92 |
| 14 | 93 | 94 | 95 | 97 | 99 | 101 | 103 | 105 | 106 |
| 15 | 105 | 106 | 108 | 110 | 112 | 114 | 117 | 118 | 119 |
| 16 | 118 | 119 | 120 | 123 | 125 | 128 | 130 | 132 | 133 |
| 17 | 131 | 132 | 133 | 136 | 138 | 141 | 144 | 145 | 147 |
| 18 | 143 | 144 | 146 | 149 | 152 | 155 | 157 | 159 | 160 |
| 19 | 155 | 157 | 159 | 161 | 165 | 168 | 171 | 173 | 174 |
| 20 | 168 | 169 | 171 | 174 | 177 | 181 | 184 | 186 | 188 |
| 21 | 179 | 181 | 183 | 186 | 190 | 194 | 197 | 199 | 201 |
| 22 | 191 | 192 | 195 | 198 | 202 | 206 | 210 | 212 | 214 |
| 23 | 202 | 204 | 206 | 210 | 214 | 218 | 222 | 224 | 227 |
| 24 | 213 | 215 | 217 | 221 | 225 | 230 | 234 | 237 | 239 |
| 25 | 223 | 225 | 227 | 232 | 236 | 241 | 246 | 248 | 251 |
| 26 | 233 | 235 | 238 | 242 | 247 | 252 | 257 | 259 | 262 |
| 27 | 242 | 245 | 247 | 252 | 257 | 262 | 267 | 270 | 273 |
| 28 | 251 | 254 | 256 | 261 | 267 | 272 | 277 | 281 | 283 |
| 29 | 260 | 262 | 265 | 270 | 276 | 282 | 287 | 290 | 293 |
| 30 | 268 | 270 | 273 | 279 | 285 | 291 | 296 | 300 | 303 |
| 31 | 275 | 278 | 281 | 287 | 293 | 299 | 305 | 308 | 311 |
| 32 | 282 | 285 | 288 | 294 | 300 | 307 | 313 | 317 | 320 |
| 33 | 288 | 291 | 295 | 301 | 307 | 314 | 320 | 324 | 328 |
| 34 | 294 | 297 | 301 | 307 | 314 | 321 | 328 | 331 | 335 |
| 35 | 299 | 303 | 306 | 313 | 320 | 327 | 334 | 338 | 342 |
| 36 | 304 | 307 | 311 | 318 | 325 | 333 | 340 | 344 | 348 |
| 37 | 308 | 312 | 316 | 323 | 330 | 338 | 346 | 350 | 354 |
| 38 | 312 | 315 | 319 | 327 | 335 | 343 | 351 | 356 | 360 |
| 39 | 314 | 318 | 323 | 330 | 339 | 347 | 356 | 361 | 365 |
| 40 | 317 | 321 | 325 | 333 | 342 | 351 | 360 | 365 | 370 |
| 41 | 318 | 322 | 327 | 336 | 345 | 355 | 364 | 369 | 374 |
| 42 | 319 | 324 | 329 | 338 | 347 | 358 | 367 | 373 | 378 |

*GA expressed as completed gestational weeks, e.g. 12 weeks corresponds to 12+0 weeks or 84 gestational days.

Mean and variance equation for HC in males:

*E(Z*_i_) = 8.51636027823433 + [-14.49558997497378 GA_i_^-0.5^] + [-0.0002432991293945 GA_i_^2^]

*Var(Z*_i_) = 0.0248425670066804 + [0.3660305465083908 GA_i_^-1^] + [-0.1866960903960692 GA_i_^-0.5^] + [-0.0000123263318793 GA_i_^2^] + [0.000022532442754 GA_i_^-.5^GA_i_^2^] + [2.10904660584e-09 GA_i_^4^]

Supplementary Table 2c. Estimated head circumference (HC) in mm by gestational age (GA) for females, Standard deviations (SD).

| GA (weeks*) | -3 SD | -2 SD | -1 SD | Median | +1 SD | +2 SD | +3 SD |
| --- | --- | --- | --- | --- | --- | --- | --- |
| 12 | 68 | 70 | 72 | 74 | 76 | 78 | 80 |
| 13 | 79 | 81 | 84 | 86 | 88 | 91 | 93 |
| 14 | 91 | 93 | 96 | 99 | 101 | 104 | 107 |
| 15 | 103 | 106 | 108 | 111 | 114 | 118 | 121 |
| 16 | 115 | 118 | 121 | 124 | 128 | 131 | 135 |
| 17 | 126 | 130 | 133 | 137 | 141 | 145 | 149 |
| 18 | 138 | 142 | 146 | 150 | 154 | 158 | 163 |
| 19 | 149 | 154 | 158 | 163 | 167 | 172 | 177 |
| 20 | 161 | 165 | 170 | 175 | 180 | 185 | 191 |
| 21 | 172 | 177 | 182 | 187 | 193 | 198 | 204 |
| 22 | 182 | 188 | 193 | 199 | 205 | 211 | 217 |
| 23 | 193 | 198 | 204 | 211 | 217 | 223 | 230 |
| 24 | 203 | 209 | 215 | 222 | 228 | 235 | 243 |
| 25 | 212 | 219 | 225 | 232 | 240 | 247 | 255 |
| 26 | 221 | 228 | 235 | 243 | 250 | 258 | 267 |
| 27 | 230 | 237 | 245 | 253 | 261 | 269 | 278 |
| 28 | 238 | 246 | 254 | 262 | 271 | 280 | 289 |
| 29 | 246 | 254 | 263 | 271 | 280 | 289 | 299 |
| 30 | 253 | 262 | 271 | 280 | 289 | 299 | 309 |
| 31 | 260 | 269 | 278 | 288 | 298 | 308 | 318 |
| 32 | 267 | 276 | 285 | 295 | 306 | 316 | 327 |
| 33 | 273 | 282 | 292 | 302 | 313 | 324 | 335 |
| 34 | 278 | 288 | 298 | 309 | 320 | 331 | 343 |
| 35 | 283 | 293 | 304 | 315 | 326 | 338 | 351 |
| 36 | 287 | 298 | 309 | 320 | 332 | 345 | 357 |
| 37 | 291 | 302 | 314 | 326 | 338 | 351 | 364 |
| 38 | 295 | 306 | 318 | 330 | 343 | 356 | 370 |
| 39 | 298 | 309 | 322 | 334 | 347 | 361 | 375 |
| 40 | 300 | 312 | 325 | 338 | 351 | 365 | 380 |
| 41 | 302 | 314 | 327 | 341 | 355 | 369 | 385 |
| 42 | 304 | 316 | 330 | 343 | 358 | 373 | 389 |

*GA expressed as completed gestational weeks, e.g. 12 weeks corresponds to 12+0 weeks or 84 gestational days.

Mean and variance equation for HC in females:

*E(Z*_i_) = 8.43094715430745 + [-14.20131801584147 GA_i_^-0.5^] + [-0.0002270435072072 GA_i_^2^]

*Var(Z*_i_) = 0.0058741070210406 + [0.0669701657710137 GA_i_^-1^] + [-0.037011092570873 GA_i_^-0.5^] + [-1.45932208746e-06 GA_i_^2^] + [2.11182970655e-06 GA_i_^-0.5^GA_i_^2^] + [4.38262718998e-10 GA_i_^4^]

Supplementary Table 2d. Estimated head circumference (HC) in mm by gestational age (GA) for females, percentiles.

| GA (weeks*) | 2.5^th^ | 5^th^ | 10^th^ | 25^th^ | Median | 75^th^ | 90^th^ | 95^th^ | 97.5^th^ |
| --- | --- | --- | --- | --- | --- | --- | --- | --- | --- |
| 12 | 70 | 70 | 71 | 72 | 74 | 75 | 76 | 77 | 78 |
| 13 | 82 | 82 | 83 | 84 | 86 | 88 | 89 | 90 | 91 |
| 14 | 94 | 94 | 95 | 97 | 99 | 100 | 102 | 103 | 104 |
| 15 | 106 | 107 | 108 | 109 | 111 | 113 | 115 | 116 | 117 |
| 16 | 118 | 119 | 120 | 122 | 124 | 127 | 129 | 130 | 131 |
| 17 | 130 | 131 | 132 | 135 | 137 | 140 | 142 | 143 | 145 |
| 18 | 142 | 143 | 145 | 147 | 150 | 153 | 155 | 157 | 158 |
| 19 | 154 | 155 | 157 | 160 | 163 | 166 | 169 | 170 | 172 |
| 20 | 165 | 167 | 169 | 172 | 175 | 178 | 181 | 183 | 185 |
| 21 | 177 | 178 | 180 | 184 | 187 | 191 | 194 | 196 | 198 |
| 22 | 188 | 190 | 192 | 195 | 199 | 203 | 207 | 209 | 211 |
| 23 | 199 | 200 | 203 | 206 | 211 | 215 | 219 | 221 | 223 |
| 24 | 209 | 211 | 213 | 217 | 222 | 226 | 230 | 233 | 235 |
| 25 | 219 | 221 | 224 | 228 | 232 | 237 | 242 | 244 | 247 |
| 26 | 228 | 231 | 233 | 238 | 243 | 248 | 253 | 256 | 258 |
| 27 | 238 | 240 | 243 | 247 | 253 | 258 | 263 | 266 | 269 |
| 28 | 246 | 249 | 252 | 257 | 262 | 268 | 273 | 276 | 279 |
| 29 | 254 | 257 | 260 | 265 | 271 | 277 | 283 | 286 | 289 |
| 30 | 262 | 265 | 268 | 274 | 280 | 286 | 292 | 295 | 298 |
| 31 | 270 | 272 | 276 | 281 | 288 | 294 | 300 | 304 | 307 |
| 32 | 276 | 279 | 283 | 289 | 295 | 302 | 308 | 312 | 316 |
| 33 | 283 | 286 | 289 | 295 | 302 | 309 | 316 | 320 | 324 |
| 34 | 288 | 292 | 295 | 302 | 309 | 316 | 323 | 327 | 331 |
| 35 | 294 | 297 | 301 | 307 | 315 | 323 | 330 | 334 | 338 |
| 36 | 298 | 302 | 306 | 313 | 320 | 328 | 336 | 340 | 344 |
| 37 | 303 | 306 | 310 | 317 | 326 | 334 | 341 | 346 | 350 |
| 38 | 307 | 310 | 314 | 322 | 330 | 339 | 346 | 351 | 355 |
| 39 | 310 | 314 | 318 | 326 | 334 | 343 | 351 | 356 | 360 |
| 40 | 313 | 317 | 321 | 329 | 338 | 347 | 355 | 360 | 365 |
| 41 | 315 | 319 | 324 | 332 | 341 | 350 | 359 | 364 | 369 |
| 42 | 317 | 321 | 326 | 334 | 343 | 353 | 362 | 368 | 372 |

*GA expressed as completed gestational weeks, e.g. 12 weeks corresponds to 12+0 weeks or 84 gestational days.

Mean and variance equation for HC in females:

*E(Z*_i_) = 8.43094715430745 + [-14.20131801584147 GA_i_^-0.5^] + [-0.0002270435072072 GA_i_^2^]

*Var(Z*_i_) = 0.0058741070210406 + [0.0669701657710137 GA_i_^-1^] + [-0.037011092570873 GA_i_^-0.5^] + [-1.45932208746e-06 GA_i_^2^] + [2.11182970655e-06 GA_i_^-0.5^GA_i_^2^] + [4.38262718998e-10 GA_i_^4^]
