# Supplementary material for: Swedish intrauterine growth reference ranges of biometric measurements of fetal head, abdomen and femur
Source: Sci Rep. 2020 Dec 31;10:22441. doi: 10.1038/s41598-020-79797-8 (PMC7775468; doi:10.1038/s41598-020-79797-8)
Supplement: Supplementary file 3 — Supplementary Table 3. [file 41598_2020_79797_MOESM3_ESM.docx]

Supplementary Table 3a. Estimated mean abdominal diameter (MAD) in mm by gestational age (GA) for males, Standard deviations (SD).

| GA (weeks*) | -3 SD | -2 SD | -1 SD | Median | +1 SD | +2 SD | +3 SD |
| --- | --- | --- | --- | --- | --- | --- | --- |
| 12 | 15 | 16 | 17 | 18 | 19 | 20 | 21 |
| 13 | 19 | 20 | 21 | 22 | 23 | 24 | 25 |
| 14 | 22 | 23 | 24 | 25 | 27 | 28 | 29 |
| 15 | 26 | 27 | 28 | 29 | 30 | 32 | 33 |
| 16 | 29 | 30 | 32 | 33 | 34 | 36 | 37 |
| 17 | 33 | 34 | 35 | 37 | 38 | 40 | 42 |
| 18 | 36 | 38 | 39 | 41 | 42 | 44 | 46 |
| 19 | 39 | 41 | 43 | 45 | 46 | 48 | 50 |
| 20 | 43 | 45 | 47 | 48 | 50 | 53 | 55 |
| 21 | 46 | 48 | 50 | 52 | 54 | 57 | 59 |
| 22 | 50 | 52 | 54 | 56 | 58 | 61 | 63 |
| 23 | 53 | 55 | 57 | 60 | 62 | 65 | 68 |
| 24 | 56 | 58 | 61 | 63 | 66 | 69 | 72 |
| 25 | 59 | 62 | 64 | 67 | 70 | 73 | 76 |
| 26 | 62 | 65 | 68 | 71 | 74 | 77 | 80 |
| 27 | 66 | 68 | 71 | 74 | 78 | 81 | 84 |
| 28 | 69 | 72 | 75 | 78 | 81 | 85 | 88 |
| 29 | 72 | 75 | 78 | 81 | 85 | 89 | 92 |
| 30 | 74 | 78 | 81 | 85 | 88 | 92 | 97 |
| 31 | 77 | 81 | 84 | 88 | 92 | 96 | 100 |
| 32 | 80 | 84 | 87 | 91 | 96 | 100 | 104 |
| 33 | 83 | 87 | 91 | 95 | 99 | 104 | 108 |
| 34 | 85 | 89 | 94 | 98 | 103 | 107 | 112 |
| 35 | 88 | 92 | 97 | 101 | 106 | 111 | 116 |
| 36 | 91 | 95 | 100 | 104 | 109 | 115 | 120 |
| 37 | 93 | 98 | 102 | 107 | 113 | 118 | 124 |
| 38 | 96 | 100 | 105 | 110 | 116 | 122 | 128 |
| 39 | 98 | 103 | 108 | 113 | 119 | 125 | 131 |
| 40 | 100 | 105 | 111 | 116 | 122 | 129 | 135 |
| 41 | 103 | 108 | 113 | 119 | 126 | 132 | 139 |
| 42 | 105 | 110 | 116 | 122 | 129 | 135 | 143 |

*GA expressed as completed gestational weeks, e.g. 12 weeks corresponds to 12+0 weeks or 84 gestational days.

Mean and variance equation for MAD in males:

*E(Z*_i_) = 6.694636716322746 + [-46.08213295655469 GA_i_^-2^] + {-12.07043478799945 GA_i_^-0.5^]

*Var(Z*_i_) = 0.0368950959781046 + [634.3569826060437 GA_i_^-4^] + [8.245737260111882 GA_i_^-2^] + [-0.4214438048547132 GA_i_^-0.5^] + [-26.58191816507051 GA_i_^-2^GA_i_^-0.5^] + [1.282908297335053 GA_i_^-1^]

Supplementary Table 3b. Estimated mean abdominal diameter (MAD) in mm by gestational age (GA) for males, percentiles.

| GA (weeks*) | 2.5^th^ | 5^th^ | 10^th^ | 25^th^ | Median | 75^th^ | 90^th^ | 95^th^ | 97.5^th^ |
| --- | --- | --- | --- | --- | --- | --- | --- | --- | --- |
| 12 | 16 | 16 | 17 | 17 | 18 | 19 | 19 | 20 | 20 |
| 13 | 20 | 20 | 20 | 21 | 22 | 22 | 23 | 23 | 24 |
| 14 | 23 | 24 | 24 | 25 | 25 | 26 | 27 | 27 | 28 |
| 15 | 27 | 27 | 28 | 28 | 29 | 30 | 31 | 31 | 32 |
| 16 | 30 | 31 | 31 | 32 | 33 | 34 | 35 | 35 | 36 |
| 17 | 34 | 34 | 35 | 36 | 37 | 38 | 39 | 39 | 40 |
| 18 | 38 | 38 | 39 | 40 | 41 | 42 | 43 | 44 | 44 |
| 19 | 41 | 42 | 42 | 43 | 45 | 46 | 47 | 48 | 48 |
| 20 | 45 | 45 | 46 | 47 | 48 | 50 | 51 | 52 | 52 |
| 21 | 48 | 49 | 50 | 51 | 52 | 54 | 55 | 56 | 57 |
| 22 | 52 | 52 | 53 | 55 | 56 | 58 | 59 | 60 | 61 |
| 23 | 55 | 56 | 57 | 58 | 60 | 61 | 63 | 64 | 65 |
| 24 | 59 | 59 | 60 | 62 | 63 | 65 | 67 | 68 | 69 |
| 25 | 62 | 63 | 64 | 65 | 67 | 69 | 71 | 72 | 73 |
| 26 | 65 | 66 | 67 | 69 | 71 | 73 | 75 | 76 | 77 |
| 27 | 68 | 69 | 70 | 72 | 74 | 76 | 78 | 80 | 81 |
| 28 | 72 | 73 | 74 | 76 | 78 | 80 | 82 | 83 | 85 |
| 29 | 75 | 76 | 77 | 79 | 81 | 84 | 86 | 87 | 88 |
| 30 | 78 | 79 | 80 | 82 | 85 | 87 | 90 | 91 | 92 |
| 31 | 81 | 82 | 83 | 86 | 88 | 91 | 93 | 95 | 96 |
| 32 | 84 | 85 | 86 | 89 | 91 | 94 | 97 | 98 | 100 |
| 33 | 87 | 88 | 89 | 92 | 95 | 98 | 100 | 102 | 103 |
| 34 | 90 | 91 | 92 | 95 | 98 | 101 | 104 | 106 | 107 |
| 35 | 92 | 94 | 95 | 98 | 101 | 104 | 107 | 109 | 111 |
| 36 | 95 | 97 | 98 | 101 | 104 | 108 | 111 | 113 | 114 |
| 37 | 98 | 99 | 101 | 104 | 107 | 111 | 114 | 116 | 118 |
| 38 | 100 | 102 | 104 | 107 | 110 | 114 | 118 | 120 | 121 |
| 39 | 103 | 105 | 107 | 110 | 113 | 117 | 121 | 123 | 125 |
| 40 | 106 | 107 | 109 | 113 | 116 | 120 | 124 | 126 | 128 |
| 41 | 108 | 110 | 112 | 115 | 119 | 123 | 127 | 130 | 132 |
| 42 | 111 | 112 | 114 | 118 | 122 | 127 | 131 | 133 | 135 |

*GA expressed as completed gestational weeks, e.g. 12 weeks corresponds to 12+0 weeks or 84 gestational days.

Mean and variance equation for MAD in males:

*E(Z*_i_) = 6.694636716322746 + [-46.08213295655469 GA_i_^-2^] + {-12.07043478799945 GA_i_^-0.5^]

*Var(Z*_i_) = 0.0368950959781046 + [634.3569826060437 GA_i_^-4^] + [8.245737260111882 GA_i_^-2^] + [-0.4214438048547132 GA_i_^-0.5^] + [-26.58191816507051 GA_i_^-2^GA_i_^-0.5^] + [1.282908297335053 GA_i_^-1^]

Supplementary Table 3c. Estimated mean abdominal diameter (MAD) in mm by gestational age (GA) for females, Standard deviations (SD).

| GA (weeks*) | -3 SD | -2 SD | -1 SD | Median | +1 SD | +2 SD | +3 SD |
| --- | --- | --- | --- | --- | --- | --- | --- |
| 12 | 15 | 16 | 17 | 18 | 19 | 20 | 21 |
| 13 | 19 | 20 | 21 | 21 | 23 | 24 | 25 |
| 14 | 22 | 23 | 24 | 25 | 26 | 27 | 29 |
| 15 | 25 | 27 | 28 | 29 | 30 | 31 | 33 |
| 16 | 29 | 30 | 31 | 33 | 34 | 36 | 37 |
| 17 | 32 | 33 | 35 | 36 | 38 | 40 | 41 |
| 18 | 35 | 37 | 39 | 40 | 42 | 44 | 46 |
| 19 | 39 | 40 | 42 | 44 | 46 | 48 | 50 |
| 20 | 42 | 44 | 46 | 48 | 50 | 52 | 54 |
| 21 | 45 | 47 | 49 | 52 | 54 | 56 | 59 |
| 22 | 49 | 51 | 53 | 55 | 58 | 60 | 63 |
| 23 | 52 | 54 | 57 | 59 | 62 | 64 | 67 |
| 24 | 55 | 58 | 60 | 63 | 66 | 68 | 71 |
| 25 | 58 | 61 | 64 | 66 | 69 | 72 | 76 |
| 26 | 61 | 64 | 67 | 70 | 73 | 76 | 80 |
| 27 | 64 | 67 | 70 | 74 | 77 | 80 | 84 |
| 28 | 67 | 70 | 74 | 77 | 81 | 84 | 88 |
| 29 | 70 | 74 | 77 | 81 | 84 | 88 | 92 |
| 30 | 73 | 77 | 80 | 84 | 88 | 92 | 96 |
| 31 | 76 | 79 | 83 | 87 | 91 | 96 | 100 |
| 32 | 79 | 82 | 86 | 91 | 95 | 100 | 105 |
| 33 | 81 | 85 | 89 | 94 | 99 | 104 | 109 |
| 34 | 84 | 88 | 92 | 97 | 102 | 107 | 113 |
| 35 | 86 | 91 | 95 | 100 | 106 | 111 | 117 |
| 36 | 89 | 93 | 98 | 103 | 109 | 115 | 121 |
| 37 | 91 | 96 | 101 | 107 | 112 | 119 | 125 |
| 38 | 93 | 98 | 104 | 110 | 116 | 122 | 129 |
| 39 | 95 | 101 | 107 | 113 | 119 | 126 | 133 |
| 40 | 98 | 103 | 109 | 116 | 122 | 129 | 137 |
| 41 | 100 | 106 | 112 | 119 | 126 | 133 | 141 |
| 42 | 102 | 108 | 115 | 121 | 129 | 137 | 145 |

*GA expressed as completed gestational weeks, e.g. 12 weeks corresponds to 12+0 weeks or 84 gestational days.

Mean and variance equation for MAD in females:

*E(Z*_i_) = 6.719175567245759 + [-41.02386170503235 GA_i_^-2^] + [-12.28921974746845 GA_i_^-0.5^]

*Var(Z*_i_) = 0.0550131949982896 + [681.0829298342567 GA_i_^-4^] + [10.85654875687294 GA_i_^-2^] + [-0.6173105870311364 GA_i_^-0.5^] + [-33.23519349739 GA_i_^-2^GA_i_^-0.5^] + [1.811973328348624 GA_i_^-1^]

Supplementary Table 3d. Estimated mean abdominal diameter (MAD) in mm by gestational age (GA) for females, percentiles.

| GA (weeks*) | 2.5^th^ | 5^th^ | 10^th^ | 25^th^ | Median | 75^th^ | 90^th^ | 95^th^ | 97.5^th^ |
| --- | --- | --- | --- | --- | --- | --- | --- | --- | --- |
| 12 | 16 | 16 | 17 | 17 | 18 | 19 | 19 | 20 | 20 |
| 13 | 20 | 20 | 20 | 21 | 21 | 22 | 23 | 23 | 24 |
| 14 | 23 | 23 | 24 | 24 | 25 | 26 | 27 | 27 | 27 |
| 15 | 27 | 27 | 27 | 28 | 29 | 30 | 31 | 31 | 31 |
| 16 | 30 | 30 | 31 | 32 | 33 | 34 | 35 | 35 | 36 |
| 17 | 34 | 34 | 35 | 35 | 36 | 38 | 39 | 39 | 40 |
| 18 | 37 | 38 | 38 | 39 | 40 | 41 | 43 | 43 | 44 |
| 19 | 41 | 41 | 42 | 43 | 44 | 45 | 47 | 47 | 48 |
| 20 | 44 | 45 | 45 | 47 | 48 | 49 | 51 | 51 | 52 |
| 21 | 48 | 48 | 49 | 50 | 52 | 53 | 55 | 55 | 56 |
| 22 | 51 | 52 | 52 | 54 | 55 | 57 | 59 | 59 | 60 |
| 23 | 54 | 55 | 56 | 57 | 59 | 61 | 62 | 63 | 64 |
| 24 | 58 | 58 | 59 | 61 | 63 | 65 | 66 | 67 | 68 |
| 25 | 61 | 62 | 63 | 64 | 66 | 68 | 70 | 71 | 72 |
| 26 | 64 | 65 | 66 | 68 | 70 | 72 | 74 | 75 | 76 |
| 27 | 67 | 68 | 69 | 71 | 74 | 76 | 78 | 79 | 80 |
| 28 | 71 | 72 | 73 | 75 | 77 | 79 | 82 | 83 | 84 |
| 29 | 74 | 75 | 76 | 78 | 81 | 83 | 85 | 87 | 88 |
| 30 | 77 | 78 | 79 | 81 | 84 | 87 | 89 | 91 | 92 |
| 31 | 80 | 81 | 82 | 85 | 87 | 90 | 93 | 94 | 96 |
| 32 | 83 | 84 | 85 | 88 | 91 | 94 | 96 | 98 | 100 |
| 33 | 85 | 87 | 88 | 91 | 94 | 97 | 100 | 102 | 103 |
| 34 | 88 | 89 | 91 | 94 | 97 | 100 | 104 | 105 | 107 |
| 35 | 91 | 92 | 94 | 97 | 100 | 104 | 107 | 109 | 111 |
| 36 | 93 | 95 | 97 | 100 | 103 | 107 | 111 | 113 | 115 |
| 37 | 96 | 98 | 100 | 103 | 107 | 110 | 114 | 116 | 118 |
| 38 | 99 | 100 | 102 | 106 | 110 | 114 | 118 | 120 | 122 |
| 39 | 101 | 103 | 105 | 109 | 113 | 117 | 121 | 123 | 126 |
| 40 | 104 | 105 | 108 | 111 | 116 | 120 | 124 | 127 | 129 |
| 41 | 106 | 108 | 110 | 114 | 119 | 123 | 128 | 130 | 133 |
| 42 | 108 | 110 | 113 | 117 | 121 | 126 | 131 | 134 | 136 |

*GA expressed as completed gestational weeks, e.g. 12 weeks corresponds to 12+0 weeks or 84 gestational days.

Mean and variance equation for MAD in females:

*E(Z*_i_) = 6.719175567245759 + [-41.02386170503235 GA_i_^-2^] + [-12.28921974746845 GA_i_^-0.5^]

*Var(Z*_i_) = 0.0550131949982896 + [681.0829298342567 GA_i_^-4^] + [10.85654875687294 GA_i_^-2^] + [-0.6173105870311364 GA_i_^-0.5^] + [-33.23519349739 GA_i_^-2^GA_i_^-0.5^] + [1.811973328348624 GA_i_^-1^]
