# Supplementary material for: Swedish intrauterine growth reference ranges of biometric measurements of fetal head, abdomen and femur
Source: Sci Rep. 2020 Dec 31;10:22441. doi: 10.1038/s41598-020-79797-8 (PMC7775468; doi:10.1038/s41598-020-79797-8)
Supplement: Supplementary file 4 — Supplementary Table 4. [file 41598_2020_79797_MOESM4_ESM.docx]

Supplementary Table 4a. Estimated abdominal circumference (AC) in mm by gestational age (GA) for males, Standard deviations (SD).

| GA (weeks*) | -3 SD | -2 SD | -1 SD | Median | +1 SD | +2 SD | +3 SD |
| --- | --- | --- | --- | --- | --- | --- | --- |
| 12 | 48 | 51 | 54 | 57 | 60 | 64 | 68 |
| 13 | 59 | 62 | 65 | 69 | 72 | 76 | 80 |
| 14 | 70 | 74 | 77 | 81 | 85 | 89 | 93 |
| 15 | 82 | 85 | 89 | 93 | 97 | 102 | 106 |
| 16 | 93 | 97 | 101 | 105 | 110 | 115 | 120 |
| 17 | 104 | 108 | 113 | 118 | 123 | 128 | 133 |
| 18 | 115 | 120 | 125 | 130 | 136 | 141 | 147 |
| 19 | 126 | 131 | 137 | 143 | 149 | 155 | 161 |
| 20 | 137 | 142 | 148 | 155 | 161 | 168 | 175 |
| 21 | 147 | 154 | 160 | 167 | 174 | 181 | 189 |
| 22 | 158 | 164 | 171 | 179 | 186 | 194 | 203 |
| 23 | 168 | 175 | 183 | 191 | 199 | 207 | 216 |
| 24 | 178 | 186 | 194 | 202 | 211 | 220 | 230 |
| 25 | 188 | 196 | 205 | 214 | 223 | 233 | 243 |
| 26 | 198 | 207 | 216 | 225 | 235 | 246 | 257 |
| 27 | 207 | 217 | 226 | 236 | 247 | 258 | 270 |
| 28 | 217 | 226 | 237 | 247 | 259 | 270 | 283 |
| 29 | 226 | 236 | 247 | 258 | 270 | 283 | 296 |
| 30 | 235 | 246 | 257 | 269 | 282 | 295 | 308 |
| 31 | 244 | 255 | 267 | 280 | 293 | 307 | 321 |
| 32 | 252 | 264 | 277 | 290 | 304 | 318 | 333 |
| 33 | 261 | 273 | 286 | 300 | 315 | 330 | 346 |
| 34 | 269 | 282 | 296 | 310 | 325 | 341 | 358 |
| 35 | 277 | 291 | 305 | 320 | 336 | 353 | 370 |
| 36 | 285 | 299 | 314 | 330 | 346 | 364 | 382 |
| 37 | 292 | 307 | 323 | 339 | 357 | 375 | 394 |
| 38 | 300 | 315 | 332 | 349 | 367 | 386 | 406 |
| 39 | 307 | 323 | 340 | 358 | 377 | 397 | 417 |
| 40 | 314 | 331 | 349 | 367 | 387 | 407 | 429 |
| 41 | 322 | 339 | 357 | 376 | 397 | 418 | 440 |
| 42 | 328 | 346 | 365 | 385 | 406 | 428 | 452 |

*GA expressed as completed gestational weeks, e.g. 12 weeks corresponds to 12+0 weeks or 84 gestational days.

Mean and variance equation for AC in males:

*E(Z*_i_) = 7.781280922525466 + [-54.23667732947342 GA_i_^-2^] + [-11.64505205666184 GA_i_^-0.5^]

*Var(Z*_i_) = 0.0283664387723667 + [457.887085282124 GA_i_^-4^] + [5.640268297900142 GA_i_^-2^] + [-0.3062461437502572 GA_i_^-0.5^] + [-18.25062125358063 GA_i_^-2^GA_i_^-0.5^] + [0.9054085884530401 GA_i_^-1^]

Supplementary Table 4b. Estimated abdominal circumference (AC) in mm by gestational age (GA) for males, percentiles.

| GA (weeks*) | 2.5^th^ | 5^th^ | 10^th^ | 25^th^ | Median | 75^th^ | 90^th^ | 95^th^ | 97.5^th^ |
| --- | --- | --- | --- | --- | --- | --- | --- | --- | --- |
| 12 | 51 | 52 | 53 | 55 | 57 | 59 | 61 | 63 | 64 |
| 13 | 62 | 63 | 64 | 66 | 69 | 71 | 73 | 75 | 76 |
| 14 | 74 | 75 | 76 | 78 | 81 | 83 | 86 | 87 | 88 |
| 15 | 85 | 87 | 88 | 90 | 93 | 96 | 98 | 100 | 101 |
| 16 | 97 | 98 | 100 | 102 | 105 | 108 | 111 | 113 | 114 |
| 17 | 109 | 110 | 112 | 115 | 118 | 121 | 124 | 126 | 128 |
| 18 | 120 | 122 | 124 | 127 | 130 | 134 | 137 | 139 | 141 |
| 19 | 131 | 133 | 135 | 139 | 143 | 147 | 150 | 153 | 155 |
| 20 | 143 | 145 | 147 | 150 | 155 | 159 | 163 | 166 | 168 |
| 21 | 154 | 156 | 158 | 162 | 167 | 172 | 176 | 179 | 181 |
| 22 | 165 | 167 | 169 | 174 | 179 | 184 | 189 | 192 | 194 |
| 23 | 176 | 178 | 181 | 185 | 191 | 196 | 201 | 204 | 207 |
| 24 | 186 | 189 | 192 | 197 | 202 | 208 | 214 | 217 | 220 |
| 25 | 197 | 199 | 202 | 208 | 214 | 220 | 226 | 230 | 233 |
| 26 | 207 | 210 | 213 | 219 | 225 | 232 | 238 | 242 | 245 |
| 27 | 217 | 220 | 224 | 230 | 236 | 244 | 250 | 254 | 258 |
| 28 | 227 | 230 | 234 | 240 | 247 | 255 | 262 | 266 | 270 |
| 29 | 237 | 240 | 244 | 251 | 258 | 266 | 274 | 278 | 282 |
| 30 | 246 | 250 | 254 | 261 | 269 | 277 | 285 | 290 | 294 |
| 31 | 256 | 259 | 264 | 271 | 280 | 288 | 297 | 302 | 306 |
| 32 | 265 | 269 | 273 | 281 | 290 | 299 | 308 | 313 | 318 |
| 33 | 274 | 278 | 283 | 291 | 300 | 310 | 319 | 324 | 329 |
| 34 | 282 | 287 | 292 | 300 | 310 | 320 | 330 | 336 | 341 |
| 35 | 291 | 296 | 301 | 310 | 320 | 331 | 341 | 347 | 352 |
| 36 | 300 | 304 | 310 | 319 | 330 | 341 | 351 | 358 | 363 |
| 37 | 308 | 313 | 318 | 328 | 339 | 351 | 362 | 368 | 374 |
| 38 | 316 | 321 | 327 | 337 | 349 | 361 | 372 | 379 | 385 |
| 39 | 324 | 329 | 335 | 346 | 358 | 371 | 382 | 390 | 396 |
| 40 | 332 | 337 | 344 | 355 | 367 | 380 | 392 | 400 | 406 |
| 41 | 340 | 345 | 352 | 363 | 376 | 390 | 402 | 410 | 417 |
| 42 | 347 | 353 | 360 | 372 | 385 | 399 | 412 | 420 | 427 |

*GA expressed as completed gestational weeks, e.g. 12 weeks corresponds to 12+0 weeks or 84 gestational days.

Mean and variance equation for AC in males:

*E(Z*_i_) = 7.781280922525466 + [-54.23667732947342 GA_i_^-2^] + [-11.64505205666184 GA_i_^-0.5^]

*Var(Z*_i_) = 0.0283664387723667 + [457.887085282124 GA_i_^-4^] + [5.640268297900142 GA_i_^-2^] + [-0.3062461437502572 GA_i_^-0.5^] + [-18.25062125358063 GA_i_^-2^GA_i_^-0.5^] + [0.9054085884530401 GA_i_^-1^]

Supplementary Table 4c. Estimated abdominal circumference (AC) in mm by gestational age (GA) for females, Standard deviations (SD).

| GA (weeks*) | -3 SD | -2 SD | -1 SD | Median | +1 SD | +2 SD | +3 SD |
| --- | --- | --- | --- | --- | --- | --- | --- |
| 12 | 50 | 52 | 55 | 57 | 60 | 63 | 66 |
| 13 | 60 | 63 | 66 | 69 | 72 | 75 | 79 |
| 14 | 70 | 73 | 77 | 80 | 84 | 88 | 92 |
| 15 | 81 | 84 | 88 | 92 | 97 | 101 | 106 |
| 16 | 91 | 95 | 100 | 104 | 109 | 114 | 120 |
| 17 | 102 | 106 | 111 | 117 | 122 | 127 | 133 |
| 18 | 112 | 118 | 123 | 129 | 134 | 141 | 147 |
| 19 | 123 | 129 | 135 | 141 | 147 | 154 | 161 |
| 20 | 134 | 140 | 146 | 153 | 160 | 167 | 174 |
| 21 | 144 | 151 | 157 | 165 | 172 | 180 | 188 |
| 22 | 154 | 161 | 169 | 176 | 184 | 193 | 202 |
| 23 | 165 | 172 | 180 | 188 | 197 | 206 | 215 |
| 24 | 175 | 183 | 191 | 200 | 209 | 218 | 228 |
| 25 | 184 | 193 | 202 | 211 | 221 | 231 | 242 |
| 26 | 194 | 203 | 212 | 222 | 233 | 244 | 255 |
| 27 | 203 | 213 | 223 | 234 | 245 | 256 | 268 |
| 28 | 212 | 223 | 233 | 244 | 256 | 268 | 281 |
| 29 | 221 | 232 | 243 | 255 | 268 | 281 | 294 |
| 30 | 230 | 242 | 253 | 266 | 279 | 293 | 307 |
| 31 | 239 | 251 | 263 | 276 | 290 | 305 | 320 |
| 32 | 247 | 260 | 273 | 287 | 301 | 317 | 333 |
| 33 | 255 | 269 | 282 | 297 | 312 | 329 | 346 |
| 34 | 263 | 277 | 292 | 307 | 323 | 340 | 358 |
| 35 | 271 | 286 | 301 | 317 | 334 | 352 | 371 |
| 36 | 279 | 294 | 310 | 327 | 345 | 363 | 383 |
| 37 | 286 | 302 | 319 | 336 | 355 | 375 | 395 |
| 38 | 294 | 310 | 328 | 346 | 365 | 386 | 408 |
| 39 | 301 | 318 | 336 | 355 | 376 | 397 | 420 |
| 40 | 308 | 326 | 345 | 365 | 386 | 408 | 432 |
| 41 | 315 | 333 | 353 | 374 | 396 | 419 | 444 |
| 42 | 321 | 341 | 361 | 383 | 405 | 430 | 455 |

*GA expressed as completed gestational weeks, e.g. 12 weeks corresponds to 12+0 weeks or 84 gestational days.

Mean and variance equation for AC in females:

*E(Z*_i_) = 7.821645531532069 + [-45.51786297721287 GA_i_^-2^] + [-11.98237732875936 GA_i_^-0.5^]

*Var(Z*_i_) = 0.0286119458728549 + [111.2220412857541 GA_i_^-4^] + [3.4597947221643 GA_i_^-2^] + [-0.2783945706520992 GA_i_^-0.5^] + [-8.978267788249893 GA_i_^-2^GA_i_^-0.5^] + [0.7258913670274708 GA_i_^-1^]

Supplementary Table 4d. Estimated abdominal circumference (AC) in mm by gestational age (GA) for females, percentiles.

| GA (weeks*) | 2.5^th^ | 5^th^ | 10^th^ | 25^th^ | Median | 75^th^ | 90^th^ | 95^th^ | 97.5^th^ |
| --- | --- | --- | --- | --- | --- | --- | --- | --- | --- |
| 12 | 52 | 53 | 54 | 55 | 57 | 59 | 61 | 62 | 63 |
| 13 | 63 | 64 | 65 | 67 | 69 | 71 | 73 | 74 | 75 |
| 14 | 73 | 75 | 76 | 78 | 80 | 83 | 85 | 87 | 88 |
| 15 | 84 | 86 | 87 | 90 | 92 | 95 | 98 | 100 | 101 |
| 16 | 96 | 97 | 99 | 101 | 104 | 108 | 111 | 112 | 114 |
| 17 | 107 | 108 | 110 | 113 | 117 | 120 | 123 | 125 | 127 |
| 18 | 118 | 120 | 121 | 125 | 129 | 133 | 136 | 138 | 140 |
| 19 | 129 | 131 | 133 | 137 | 141 | 145 | 149 | 151 | 154 |
| 20 | 140 | 142 | 144 | 148 | 153 | 157 | 162 | 164 | 167 |
| 21 | 151 | 153 | 156 | 160 | 165 | 170 | 174 | 177 | 180 |
| 22 | 162 | 164 | 167 | 171 | 176 | 182 | 187 | 190 | 192 |
| 23 | 172 | 175 | 178 | 183 | 188 | 194 | 199 | 202 | 205 |
| 24 | 183 | 185 | 189 | 194 | 200 | 206 | 211 | 215 | 218 |
| 25 | 193 | 196 | 199 | 205 | 211 | 218 | 224 | 227 | 231 |
| 26 | 203 | 206 | 210 | 216 | 222 | 229 | 236 | 240 | 243 |
| 27 | 213 | 216 | 220 | 226 | 234 | 241 | 248 | 252 | 256 |
| 28 | 223 | 226 | 230 | 237 | 244 | 252 | 260 | 264 | 268 |
| 29 | 233 | 236 | 240 | 247 | 255 | 264 | 271 | 276 | 280 |
| 30 | 242 | 246 | 250 | 257 | 266 | 275 | 283 | 288 | 292 |
| 31 | 251 | 255 | 260 | 268 | 276 | 286 | 294 | 300 | 304 |
| 32 | 260 | 264 | 269 | 277 | 287 | 297 | 306 | 311 | 316 |
| 33 | 269 | 273 | 278 | 287 | 297 | 307 | 317 | 323 | 328 |
| 34 | 278 | 282 | 288 | 297 | 307 | 318 | 328 | 334 | 340 |
| 35 | 286 | 291 | 297 | 306 | 317 | 328 | 339 | 345 | 351 |
| 36 | 295 | 300 | 305 | 315 | 327 | 339 | 350 | 357 | 363 |
| 37 | 303 | 308 | 314 | 324 | 336 | 349 | 360 | 368 | 374 |
| 38 | 311 | 316 | 323 | 333 | 346 | 359 | 371 | 378 | 385 |
| 39 | 319 | 324 | 331 | 342 | 355 | 369 | 382 | 389 | 396 |
| 40 | 326 | 332 | 339 | 351 | 365 | 379 | 392 | 400 | 407 |
| 41 | 334 | 340 | 347 | 359 | 374 | 388 | 402 | 410 | 418 |
| 42 | 341 | 348 | 355 | 368 | 383 | 398 | 412 | 421 | 429 |

*GA expressed as completed gestational weeks, e.g. 12 weeks corresponds to 12+0 weeks or 84 gestational days.

Mean and variance equation for AC in females:

*E(Z*_i_) = 7.821645531532069 + [-45.51786297721287 GA_i_^-2^] + [-11.98237732875936 GA_i_^-0.5^]

*Var(Z*_i_) = 0.0286119458728549 + [111.2220412857541 GA_i_^-4^] + [3.4597947221643 GA_i_^-2^] + [-0.2783945706520992 GA_i_^-0.5^] + [-8.978267788249893 GA_i_^-2^GA_i_^-0.5^] + [0.7258913670274708 GA_i_^-1^]
