# Supplementary material for: Swedish intrauterine growth reference ranges of biometric measurements of fetal head, abdomen and femur
Source: Sci Rep. 2020 Dec 31;10:22441. doi: 10.1038/s41598-020-79797-8 (PMC7775468; doi:10.1038/s41598-020-79797-8)
Supplement: Supplementary file 5 — Supplementary Table 5. [file 41598_2020_79797_MOESM5_ESM.docx]

Supplementary Table 5a. Estimated femur length (FL) in mm by gestational age (GA) for males, Standard deviations (SD).

| GA (weeks*) | -3 SD | -2 SD | -1 SD | Median | +1 SD | +2 SD | +3 SD |
| --- | --- | --- | --- | --- | --- | --- | --- |
| 12 | 4 | 5 | 6 | 6 | 7 | 8 | 9 |
| 13 | 7 | 7 | 8 | 9 | 10 | 11 | 12 |
| 14 | 10 | 10 | 11 | 12 | 13 | 14 | 15 |
| 15 | 13 | 13 | 14 | 15 | 16 | 17 | 18 |
| 16 | 16 | 17 | 18 | 19 | 20 | 21 | 22 |
| 17 | 19 | 20 | 21 | 22 | 23 | 24 | 26 |
| 18 | 22 | 23 | 24 | 25 | 26 | 28 | 29 |
| 19 | 25 | 26 | 27 | 28 | 30 | 31 | 33 |
| 20 | 28 | 29 | 30 | 31 | 33 | 34 | 36 |
| 21 | 30 | 32 | 33 | 34 | 36 | 38 | 39 |
| 22 | 33 | 34 | 36 | 37 | 39 | 41 | 43 |
| 23 | 35 | 37 | 38 | 40 | 42 | 44 | 46 |
| 24 | 38 | 39 | 41 | 43 | 45 | 47 | 49 |
| 25 | 40 | 42 | 43 | 45 | 47 | 49 | 52 |
| 26 | 42 | 44 | 46 | 48 | 50 | 52 | 54 |
| 27 | 44 | 46 | 48 | 50 | 52 | 55 | 57 |
| 28 | 46 | 48 | 50 | 52 | 55 | 57 | 60 |
| 29 | 48 | 50 | 52 | 54 | 57 | 59 | 62 |
| 30 | 50 | 52 | 54 | 57 | 59 | 62 | 64 |
| 31 | 51 | 54 | 56 | 59 | 61 | 64 | 67 |
| 32 | 53 | 55 | 58 | 60 | 63 | 66 | 69 |
| 33 | 55 | 57 | 60 | 62 | 65 | 68 | 71 |
| 34 | 56 | 59 | 61 | 64 | 67 | 70 | 73 |
| 35 | 58 | 60 | 63 | 66 | 69 | 72 | 75 |
| 36 | 59 | 62 | 65 | 68 | 71 | 74 | 77 |
| 37 | 61 | 64 | 66 | 69 | 72 | 76 | 79 |
| 38 | 62 | 65 | 68 | 71 | 74 | 77 | 81 |
| 39 | 64 | 67 | 70 | 73 | 76 | 79 | 83 |
| 40 | 65 | 68 | 71 | 74 | 77 | 81 | 85 |
| 41 | 67 | 69 | 73 | 76 | 79 | 83 | 86 |
| 42 | 68 | 71 | 74 | 77 | 81 | 84 | 88 |

*GA expressed as completed gestational weeks, e.g. 12 weeks corresponds to 12+0 weeks or 84 gestational days.

Mean and variance equation for FL in males:

*E(Z*_i_) = 4.108789074759255 + [-346.9304247899768 GA_i_^-2^] + [0.0103728379807879 GA_i_^1^]

*Var(Z*_i_) = 0.0081544650055426 + [527.0642954388159 GA_i_^-4^] + [-3.623386453680172 GA_i_^-2^] + [-0.0002651463744066 GA_i_^1^] + [0.0376972877796724 GA_i_^-2^GA_i_^1^] + [2.81542307364e-06 GA_i_^2^]

Supplementary Table 5b. Estimated femur length (FL) in mm by gestational age (GA) for males, percentiles.

| GA (weeks*) | 2.5^th^ | 5^th^ | 10^th^ | 25^th^ | Median | 75^th^ | 90^th^ | 95^th^ | 97.5^th^ |
| --- | --- | --- | --- | --- | --- | --- | --- | --- | --- |
| 12 | 5 | 5 | 5 | 6 | 6 | 7 | 7 | 7 | 8 |
| 13 | 8 | 8 | 8 | 8 | 9 | 9 | 10 | 10 | 11 |
| 14 | 10 | 11 | 11 | 11 | 12 | 13 | 13 | 14 | 14 |
| 15 | 13 | 14 | 14 | 15 | 15 | 16 | 17 | 17 | 17 |
| 16 | 17 | 17 | 17 | 18 | 19 | 19 | 20 | 20 | 21 |
| 17 | 20 | 20 | 20 | 21 | 22 | 23 | 23 | 24 | 24 |
| 18 | 23 | 23 | 24 | 24 | 25 | 26 | 27 | 27 | 28 |
| 19 | 26 | 26 | 27 | 27 | 28 | 29 | 30 | 31 | 31 |
| 20 | 29 | 29 | 30 | 31 | 31 | 32 | 33 | 34 | 34 |
| 21 | 32 | 32 | 33 | 33 | 34 | 35 | 36 | 37 | 38 |
| 22 | 34 | 35 | 35 | 36 | 37 | 38 | 39 | 40 | 41 |
| 23 | 37 | 37 | 38 | 39 | 40 | 41 | 42 | 43 | 44 |
| 24 | 39 | 40 | 40 | 42 | 43 | 44 | 45 | 46 | 47 |
| 25 | 42 | 42 | 43 | 44 | 45 | 47 | 48 | 49 | 49 |
| 26 | 44 | 44 | 45 | 46 | 48 | 49 | 50 | 51 | 52 |
| 27 | 46 | 47 | 47 | 49 | 50 | 52 | 53 | 54 | 54 |
| 28 | 48 | 49 | 49 | 51 | 52 | 54 | 55 | 56 | 57 |
| 29 | 50 | 51 | 52 | 53 | 54 | 56 | 58 | 58 | 59 |
| 30 | 52 | 53 | 53 | 55 | 57 | 58 | 60 | 61 | 62 |
| 31 | 54 | 54 | 55 | 57 | 59 | 60 | 62 | 63 | 64 |
| 32 | 56 | 56 | 57 | 59 | 60 | 62 | 64 | 65 | 66 |
| 33 | 57 | 58 | 59 | 61 | 62 | 64 | 66 | 67 | 68 |
| 34 | 59 | 60 | 61 | 62 | 64 | 66 | 68 | 69 | 70 |
| 35 | 61 | 61 | 62 | 64 | 66 | 68 | 70 | 71 | 72 |
| 36 | 62 | 63 | 64 | 66 | 68 | 70 | 72 | 73 | 74 |
| 37 | 64 | 65 | 66 | 67 | 69 | 71 | 73 | 74 | 75 |
| 38 | 65 | 66 | 67 | 69 | 71 | 73 | 75 | 76 | 77 |
| 39 | 67 | 68 | 69 | 71 | 73 | 75 | 77 | 78 | 79 |
| 40 | 68 | 69 | 70 | 72 | 74 | 76 | 78 | 80 | 81 |
| 41 | 70 | 71 | 72 | 74 | 76 | 78 | 80 | 81 | 83 |
| 42 | 71 | 72 | 73 | 75 | 77 | 80 | 82 | 83 | 84 |

*GA expressed as completed gestational weeks, e.g. 12 weeks corresponds to 12+0 weeks or 84 gestational days.

Mean and variance equation for FL in males:

*E(Z*_i_) = 4.108789074759255 + [-346.9304247899768 GA_i_^-2^] + [0.0103728379807879 GA_i_^1^]

*Var(Z*_i_) = 0.0081544650055426 + [527.0642954388159 GA_i_^-4^] + [-3.623386453680172 GA_i_^-2^] + [-0.0002651463744066 GA_i_^1^] + [0.0376972877796724 GA_i_^-2^GA_i_^1^] + [2.81542307364e-06 GA_i_^2^]

Supplementary Table 5c. Estimated femur length (FL) in mm by gestational age (GA) for females, Standard deviations (SD).

| GA (weeks*) | -3 SD | -2 SD | -1 SD | Median | +1 SD | +2 SD | +3 SD |
| --- | --- | --- | --- | --- | --- | --- | --- |
| 12 | 5 | 5 | 6 | 6 | 7 | 8 | 9 |
| 13 | 7 | 8 | 8 | 9 | 10 | 11 | 12 |
| 14 | 10 | 11 | 11 | 12 | 13 | 14 | 15 |
| 15 | 13 | 14 | 15 | 16 | 16 | 18 | 19 |
| 16 | 16 | 17 | 18 | 19 | 20 | 21 | 22 |
| 17 | 19 | 20 | 21 | 22 | 23 | 24 | 26 |
| 18 | 22 | 23 | 24 | 25 | 27 | 28 | 29 |
| 19 | 25 | 26 | 27 | 29 | 30 | 31 | 33 |
| 20 | 28 | 29 | 30 | 32 | 33 | 35 | 36 |
| 21 | 31 | 32 | 33 | 35 | 36 | 38 | 39 |
| 22 | 33 | 35 | 36 | 38 | 39 | 41 | 43 |
| 23 | 36 | 37 | 39 | 40 | 42 | 44 | 46 |
| 24 | 38 | 40 | 41 | 43 | 45 | 47 | 49 |
| 25 | 40 | 42 | 44 | 45 | 47 | 49 | 51 |
| 26 | 42 | 44 | 46 | 48 | 50 | 52 | 54 |
| 27 | 44 | 46 | 48 | 50 | 52 | 54 | 57 |
| 28 | 46 | 48 | 50 | 52 | 55 | 57 | 59 |
| 29 | 48 | 50 | 52 | 55 | 57 | 59 | 62 |
| 30 | 50 | 52 | 54 | 57 | 59 | 61 | 64 |
| 31 | 52 | 54 | 56 | 59 | 61 | 63 | 66 |
| 32 | 54 | 56 | 58 | 61 | 63 | 66 | 68 |
| 33 | 55 | 58 | 60 | 62 | 65 | 68 | 70 |
| 34 | 57 | 59 | 62 | 64 | 67 | 70 | 72 |
| 35 | 58 | 61 | 63 | 66 | 69 | 71 | 74 |
| 36 | 60 | 62 | 65 | 68 | 70 | 73 | 76 |
| 37 | 61 | 64 | 67 | 69 | 72 | 75 | 78 |
| 38 | 63 | 65 | 68 | 71 | 74 | 77 | 80 |
| 39 | 64 | 67 | 70 | 73 | 76 | 79 | 82 |
| 40 | 66 | 68 | 71 | 74 | 77 | 81 | 84 |
| 41 | 67 | 70 | 73 | 76 | 79 | 82 | 86 |
| 42 | 68 | 71 | 74 | 77 | 81 | 84 | 88 |

*GA expressed as completed gestational weeks, e.g. 12 weeks corresponds to 12+0 weeks or 84 gestational days.

Mean and variance equation for FL in females:

*E(Z*_i_) = 4.101960342625556 + [-341.1624016859801 GA_i_^-2^] + [0.0104420358599529 GA_i_^1^]

*Var(Z*_i_) = 0.0123785446749309 + [570.1822895829761 GA_i_^-4^] + [-4.773346467239806 GA_i_^-2^] + [-0.0005219388137002 GA_i_^1^] + [0.0569645385918154 GA_i_^-2^GA_i_^1^] + [6.32593894463e-06 GA_i_^2^]

Supplementary Table 5d. Estimated femur length (FL) in mm by gestational age (GA) for females, percentiles.

| GA (weeks*) | 2.5^th^ | 5^th^ | 10^th^ | 25^th^ | Median | 75^th^ | 90^th^ | 95^th^ | 97.5^th^ |
| --- | --- | --- | --- | --- | --- | --- | --- | --- | --- |
| 12 | 5 | 5 | 6 | 6 | 6 | 7 | 7 | 8 | 8 |
| 13 | 8 | 8 | 8 | 9 | 9 | 10 | 10 | 11 | 11 |
| 14 | 11 | 11 | 11 | 12 | 12 | 13 | 13 | 14 | 14 |
| 15 | 14 | 14 | 14 | 15 | 16 | 16 | 17 | 17 | 17 |
| 16 | 17 | 17 | 18 | 18 | 19 | 20 | 20 | 21 | 21 |
| 17 | 20 | 20 | 21 | 21 | 22 | 23 | 24 | 24 | 24 |
| 18 | 23 | 24 | 24 | 25 | 25 | 26 | 27 | 27 | 28 |
| 19 | 26 | 27 | 27 | 28 | 29 | 30 | 30 | 31 | 31 |
| 20 | 29 | 30 | 30 | 31 | 32 | 33 | 34 | 34 | 35 |
| 21 | 32 | 32 | 33 | 34 | 35 | 36 | 37 | 37 | 38 |
| 22 | 35 | 35 | 36 | 37 | 38 | 39 | 40 | 40 | 41 |
| 23 | 37 | 38 | 38 | 39 | 40 | 41 | 43 | 43 | 44 |
| 24 | 40 | 40 | 41 | 42 | 43 | 44 | 45 | 46 | 47 |
| 25 | 42 | 43 | 43 | 44 | 45 | 47 | 48 | 49 | 49 |
| 26 | 44 | 45 | 45 | 47 | 48 | 49 | 50 | 51 | 52 |
| 27 | 46 | 47 | 48 | 49 | 50 | 52 | 53 | 54 | 54 |
| 28 | 48 | 49 | 50 | 51 | 52 | 54 | 55 | 56 | 57 |
| 29 | 50 | 51 | 52 | 53 | 55 | 56 | 57 | 58 | 59 |
| 30 | 52 | 53 | 54 | 55 | 57 | 58 | 60 | 60 | 61 |
| 31 | 54 | 55 | 56 | 57 | 59 | 60 | 62 | 63 | 63 |
| 32 | 56 | 57 | 57 | 59 | 61 | 62 | 64 | 65 | 65 |
| 33 | 58 | 58 | 59 | 61 | 62 | 64 | 66 | 67 | 67 |
| 34 | 59 | 60 | 61 | 62 | 64 | 66 | 68 | 69 | 69 |
| 35 | 61 | 62 | 63 | 64 | 66 | 68 | 69 | 70 | 71 |
| 36 | 63 | 63 | 64 | 66 | 68 | 70 | 71 | 72 | 73 |
| 37 | 64 | 65 | 66 | 67 | 69 | 71 | 73 | 74 | 75 |
| 38 | 66 | 66 | 67 | 69 | 71 | 73 | 75 | 76 | 77 |
| 39 | 67 | 68 | 69 | 71 | 73 | 75 | 77 | 78 | 79 |
| 40 | 68 | 69 | 70 | 72 | 74 | 76 | 78 | 79 | 80 |
| 41 | 70 | 71 | 72 | 74 | 76 | 78 | 80 | 81 | 82 |
| 42 | 71 | 72 | 73 | 75 | 77 | 80 | 82 | 83 | 84 |

*GA expressed as completed gestational weeks, e.g. 12 weeks corresponds to 12+0 weeks or 84 gestational days.

Mean and variance equation for FL in females:

*E(Z*_i_) = 4.101960342625556 + [-341.1624016859801 GA_i_^-2^] + [0.0104420358599529 GA_i_^1^]

*Var(Z*_i_) = 0.0123785446749309 + [570.1822895829761 GA_i_^-4^] + [-4.773346467239806 GA_i_^-2^] + [-0.0005219388137002 GA_i_^1^] + [0.0569645385918154 GA_i_^-2^GA_i_^1^] + [6.32593894463e-06 GA_i_^2^]
