# Supplementary material for: Swedish intrauterine growth reference ranges of biometric measurements of fetal head, abdomen and femur
Source: Sci Rep. 2020 Dec 31;10:22441. doi: 10.1038/s41598-020-79797-8 (PMC7775468; doi:10.1038/s41598-020-79797-8)
Supplement: Supplementary file 6 — Supplementary Table 6. [file 41598_2020_79797_MOESM6_ESM.docx]

Supplementary Table 6a. Estimated biparietal diameter (BPD) in mm by gestational age (GA) for males and females, Standard deviations (SD).

| GA (days) | -3 SD | -2 SD | -1 SD | Median | +1 SD | +2 SD | +3 SD |
| --- | --- | --- | --- | --- | --- | --- | --- |
| 84 | 19 | 19 | 20 | 20 | 21 | 21 | 22 |
| 85 | 19 | 20 | 20 | 21 | 21 | 22 | 22 |
| 86 | 20 | 20 | 21 | 21 | 22 | 22 | 23 |
| 87 | 20 | 21 | 21 | 22 | 22 | 23 | 23 |
| 88 | 21 | 21 | 22 | 22 | 23 | 23 | 24 |
| 89 | 21 | 21 | 22 | 22 | 23 | 24 | 24 |
| 90 | 21 | 22 | 22 | 23 | 23 | 24 | 25 |
| 91 | 22 | 22 | 23 | 23 | 24 | 24 | 25 |
| 92 | 22 | 23 | 23 | 24 | 24 | 25 | 26 |
| 93 | 23 | 23 | 24 | 24 | 25 | 25 | 26 |
| 94 | 23 | 24 | 24 | 25 | 25 | 26 | 27 |
| 95 | 24 | 24 | 25 | 25 | 26 | 26 | 27 |
| 96 | 24 | 25 | 25 | 26 | 26 | 27 | 28 |
| 97 | 24 | 25 | 26 | 26 | 27 | 28 | 28 |
| 98 | 25 | 25 | 26 | 27 | 27 | 28 | 29 |
| 99 | 25 | 26 | 27 | 27 | 28 | 29 | 29 |
| 100 | 26 | 26 | 27 | 28 | 28 | 29 | 30 |
| 101 | 26 | 27 | 27 | 28 | 29 | 30 | 30 |
| 102 | 27 | 27 | 28 | 29 | 29 | 30 | 31 |
| 103 | 27 | 28 | 28 | 29 | 30 | 31 | 31 |
| 104 | 27 | 28 | 29 | 30 | 30 | 31 | 32 |
| 105 | 28 | 29 | 29 | 30 | 31 | 32 | 32 |
| 106 | 28 | 29 | 30 | 31 | 31 | 32 | 33 |
| 107 | 29 | 29 | 30 | 31 | 32 | 33 | 34 |
| 108 | 29 | 30 | 31 | 32 | 32 | 33 | 34 |
| 109 | 30 | 30 | 31 | 32 | 33 | 34 | 35 |
| 110 | 30 | 31 | 32 | 33 | 33 | 34 | 35 |
| 111 | 30 | 31 | 32 | 33 | 34 | 35 | 36 |
| 112 | 31 | 32 | 33 | 34 | 34 | 35 | 36 |
| 113 | 31 | 32 | 33 | 34 | 35 | 36 | 37 |
| 114 | 32 | 33 | 34 | 35 | 36 | 37 | 38 |
| 115 | 32 | 33 | 34 | 35 | 36 | 37 | 38 |
| 116 | 33 | 34 | 35 | 36 | 37 | 38 | 39 |
| 117 | 33 | 34 | 35 | 36 | 37 | 38 | 39 |
| 118 | 34 | 34 | 36 | 37 | 38 | 39 | 40 |
| 119 | 34 | 35 | 36 | 37 | 38 | 39 | 40 |
| 120 | 34 | 35 | 36 | 38 | 39 | 40 | 41 |
| 121 | 35 | 36 | 37 | 38 | 39 | 40 | 42 |
| 122 | 35 | 36 | 37 | 39 | 40 | 41 | 42 |
| 123 | 36 | 37 | 38 | 39 | 40 | 41 | 43 |
| 124 | 36 | 37 | 38 | 40 | 41 | 42 | 43 |
| 125 | 37 | 38 | 39 | 40 | 41 | 43 | 44 |
| 126 | 37 | 38 | 39 | 41 | 42 | 43 | 44 |
| 127 | 37 | 39 | 40 | 41 | 42 | 44 | 45 |
| 128 | 38 | 39 | 40 | 42 | 43 | 44 | 46 |
| 129 | 38 | 39 | 41 | 42 | 43 | 45 | 46 |
| 130 | 39 | 40 | 41 | 43 | 44 | 45 | 47 |
| 131 | 39 | 40 | 42 | 43 | 44 | 46 | 47 |
| 132 | 40 | 41 | 42 | 44 | 45 | 46 | 48 |
| 133 | 40 | 41 | 43 | 44 | 45 | 47 | 48 |
| 134 | 40 | 42 | 43 | 45 | 46 | 48 | 49 |
| 135 | 41 | 42 | 44 | 45 | 47 | 48 | 50 |
| 136 | 41 | 43 | 44 | 46 | 47 | 49 | 50 |
| 137 | 42 | 43 | 45 | 46 | 48 | 49 | 51 |
| 138 | 42 | 44 | 45 | 47 | 48 | 50 | 51 |
| 139 | 43 | 44 | 45 | 47 | 49 | 50 | 52 |
| 140 | 43 | 44 | 46 | 48 | 49 | 51 | 53 |
| 141 | 43 | 45 | 46 | 48 | 50 | 51 | 53 |
| 142 | 44 | 45 | 47 | 49 | 50 | 52 | 54 |
| 143 | 44 | 46 | 47 | 49 | 51 | 52 | 54 |
| 144 | 45 | 46 | 48 | 49 | 51 | 53 | 55 |
| 145 | 45 | 47 | 48 | 50 | 52 | 54 | 55 |
| 146 | 46 | 47 | 49 | 50 | 52 | 54 | 56 |
| 147 | 46 | 48 | 49 | 51 | 53 | 55 | 56 |
| 148 | 46 | 48 | 50 | 51 | 53 | 55 | 57 |
| 149 | 47 | 48 | 50 | 52 | 54 | 56 | 58 |
| 150 | 47 | 49 | 51 | 52 | 54 | 56 | 58 |
| 151 | 48 | 49 | 51 | 53 | 55 | 57 | 59 |
| 152 | 48 | 50 | 52 | 53 | 55 | 57 | 59 |
| 153 | 48 | 50 | 52 | 54 | 56 | 58 | 60 |
| 154 | 49 | 51 | 52 | 54 | 56 | 58 | 60 |
| 155 | 49 | 51 | 53 | 55 | 57 | 59 | 61 |
| 156 | 50 | 51 | 53 | 55 | 57 | 59 | 61 |
| 157 | 50 | 52 | 54 | 56 | 58 | 60 | 62 |
| 158 | 51 | 52 | 54 | 56 | 58 | 60 | 63 |
| 159 | 51 | 53 | 55 | 57 | 59 | 61 | 63 |
| 160 | 51 | 53 | 55 | 57 | 59 | 61 | 64 |
| 161 | 52 | 54 | 56 | 58 | 60 | 62 | 64 |
| 162 | 52 | 54 | 56 | 58 | 60 | 62 | 65 |
| 163 | 53 | 54 | 56 | 59 | 61 | 63 | 65 |
| 164 | 53 | 55 | 57 | 59 | 61 | 63 | 66 |
| 165 | 53 | 55 | 57 | 59 | 62 | 64 | 66 |
| 166 | 54 | 56 | 58 | 60 | 62 | 64 | 67 |
| 167 | 54 | 56 | 58 | 60 | 63 | 65 | 67 |
| 168 | 55 | 57 | 59 | 61 | 63 | 65 | 68 |
| 169 | 55 | 57 | 59 | 61 | 64 | 66 | 68 |
| 170 | 55 | 57 | 60 | 62 | 64 | 66 | 69 |
| 171 | 56 | 58 | 60 | 62 | 65 | 67 | 69 |
| 172 | 56 | 58 | 60 | 63 | 65 | 67 | 70 |
| 173 | 57 | 59 | 61 | 63 | 65 | 68 | 70 |
| 174 | 57 | 59 | 61 | 64 | 66 | 68 | 71 |
| 175 | 57 | 59 | 62 | 64 | 66 | 69 | 71 |
| 176 | 58 | 60 | 62 | 64 | 67 | 69 | 72 |
| 177 | 58 | 60 | 62 | 65 | 67 | 70 | 72 |
| 178 | 58 | 61 | 63 | 65 | 68 | 70 | 73 |
| 179 | 59 | 61 | 63 | 66 | 68 | 71 | 73 |
| 180 | 59 | 61 | 64 | 66 | 69 | 71 | 74 |
| 181 | 60 | 62 | 64 | 67 | 69 | 72 | 74 |
| 182 | 60 | 62 | 65 | 67 | 70 | 72 | 75 |
| 183 | 60 | 63 | 65 | 67 | 70 | 73 | 75 |
| 184 | 61 | 63 | 65 | 68 | 70 | 73 | 76 |
| 185 | 61 | 63 | 66 | 68 | 71 | 73 | 76 |
| 186 | 61 | 64 | 66 | 69 | 71 | 74 | 77 |
| 187 | 62 | 64 | 67 | 69 | 72 | 74 | 77 |
| 188 | 62 | 65 | 67 | 69 | 72 | 75 | 78 |
| 189 | 63 | 65 | 67 | 70 | 73 | 75 | 78 |
| 190 | 63 | 65 | 68 | 70 | 73 | 76 | 79 |
| 191 | 63 | 66 | 68 | 71 | 73 | 76 | 79 |
| 192 | 64 | 66 | 69 | 71 | 74 | 77 | 79 |
| 193 | 64 | 66 | 69 | 71 | 74 | 77 | 80 |
| 194 | 64 | 67 | 69 | 72 | 75 | 77 | 80 |
| 195 | 65 | 67 | 70 | 72 | 75 | 78 | 81 |
| 196 | 65 | 67 | 70 | 73 | 75 | 78 | 81 |
| 197 | 65 | 68 | 70 | 73 | 76 | 79 | 82 |
| 198 | 66 | 68 | 71 | 73 | 76 | 79 | 82 |
| 199 | 66 | 69 | 71 | 74 | 77 | 80 | 83 |
| 200 | 66 | 69 | 71 | 74 | 77 | 80 | 83 |
| 201 | 67 | 69 | 72 | 75 | 77 | 80 | 83 |
| 202 | 67 | 70 | 72 | 75 | 78 | 81 | 84 |
| 203 | 67 | 70 | 73 | 75 | 78 | 81 | 84 |
| 204 | 68 | 70 | 73 | 76 | 79 | 82 | 85 |
| 205 | 68 | 71 | 73 | 76 | 79 | 82 | 85 |
| 206 | 68 | 71 | 74 | 76 | 79 | 82 | 85 |
| 207 | 69 | 71 | 74 | 77 | 80 | 83 | 86 |
| 208 | 69 | 72 | 74 | 77 | 80 | 83 | 86 |
| 209 | 69 | 72 | 75 | 77 | 80 | 83 | 87 |
| 210 | 70 | 72 | 75 | 78 | 81 | 84 | 87 |
| 211 | 70 | 73 | 75 | 78 | 81 | 84 | 87 |
| 212 | 70 | 73 | 76 | 79 | 82 | 85 | 88 |
| 213 | 71 | 73 | 76 | 79 | 82 | 85 | 88 |
| 214 | 71 | 74 | 76 | 79 | 82 | 85 | 89 |
| 215 | 71 | 74 | 77 | 80 | 83 | 86 | 89 |
| 216 | 71 | 74 | 77 | 80 | 83 | 86 | 89 |
| 217 | 72 | 75 | 77 | 80 | 83 | 86 | 90 |
| 218 | 72 | 75 | 78 | 81 | 84 | 87 | 90 |
| 219 | 72 | 75 | 78 | 81 | 84 | 87 | 90 |
| 220 | 73 | 75 | 78 | 81 | 84 | 87 | 91 |
| 221 | 73 | 76 | 79 | 82 | 85 | 88 | 91 |
| 222 | 73 | 76 | 79 | 82 | 85 | 88 | 91 |
| 223 | 74 | 76 | 79 | 82 | 85 | 88 | 92 |
| 224 | 74 | 77 | 79 | 82 | 86 | 89 | 92 |
| 225 | 74 | 77 | 80 | 83 | 86 | 89 | 92 |
| 226 | 74 | 77 | 80 | 83 | 86 | 89 | 93 |
| 227 | 75 | 77 | 80 | 83 | 87 | 90 | 93 |
| 228 | 75 | 78 | 81 | 84 | 87 | 90 | 93 |
| 229 | 75 | 78 | 81 | 84 | 87 | 90 | 94 |
| 230 | 75 | 78 | 81 | 84 | 87 | 91 | 94 |
| 231 | 76 | 79 | 82 | 85 | 88 | 91 | 94 |
| 232 | 76 | 79 | 82 | 85 | 88 | 91 | 95 |
| 233 | 76 | 79 | 82 | 85 | 88 | 92 | 95 |
| 234 | 77 | 79 | 82 | 85 | 89 | 92 | 95 |
| 235 | 77 | 80 | 83 | 86 | 89 | 92 | 96 |
| 236 | 77 | 80 | 83 | 86 | 89 | 93 | 96 |
| 237 | 77 | 80 | 83 | 86 | 89 | 93 | 96 |
| 238 | 77 | 80 | 83 | 87 | 90 | 93 | 97 |
| 239 | 78 | 81 | 84 | 87 | 90 | 93 | 97 |
| 240 | 78 | 81 | 84 | 87 | 90 | 94 | 97 |
| 241 | 78 | 81 | 84 | 87 | 91 | 94 | 98 |
| 242 | 78 | 81 | 84 | 88 | 91 | 94 | 98 |
| 243 | 79 | 82 | 85 | 88 | 91 | 95 | 98 |
| 244 | 79 | 82 | 85 | 88 | 91 | 95 | 98 |
| 245 | 79 | 82 | 85 | 88 | 92 | 95 | 99 |
| 246 | 79 | 82 | 85 | 89 | 92 | 95 | 99 |
| 247 | 80 | 83 | 86 | 89 | 92 | 96 | 99 |
| 248 | 80 | 83 | 86 | 89 | 92 | 96 | 99 |
| 249 | 80 | 83 | 86 | 89 | 93 | 96 | 100 |
| 250 | 80 | 83 | 86 | 90 | 93 | 96 | 100 |
| 251 | 80 | 83 | 87 | 90 | 93 | 97 | 100 |
| 252 | 81 | 84 | 87 | 90 | 93 | 97 | 101 |
| 253 | 81 | 84 | 87 | 90 | 94 | 97 | 101 |
| 254 | 81 | 84 | 87 | 90 | 94 | 97 | 101 |
| 255 | 81 | 84 | 87 | 91 | 94 | 98 | 101 |
| 256 | 81 | 84 | 88 | 91 | 94 | 98 | 102 |
| 257 | 82 | 85 | 88 | 91 | 95 | 98 | 102 |
| 258 | 82 | 85 | 88 | 91 | 95 | 98 | 102 |
| 259 | 82 | 85 | 88 | 92 | 95 | 99 | 102 |
| 260 | 82 | 85 | 88 | 92 | 95 | 99 | 103 |
| 261 | 82 | 85 | 89 | 92 | 95 | 99 | 103 |
| 262 | 82 | 86 | 89 | 92 | 96 | 99 | 103 |
| 263 | 83 | 86 | 89 | 92 | 96 | 99 | 103 |
| 264 | 83 | 86 | 89 | 93 | 96 | 100 | 103 |
| 265 | 83 | 86 | 89 | 93 | 96 | 100 | 104 |
| 266 | 83 | 86 | 90 | 93 | 96 | 100 | 104 |
| 267 | 83 | 86 | 90 | 93 | 97 | 100 | 104 |
| 268 | 83 | 87 | 90 | 93 | 97 | 101 | 104 |
| 269 | 84 | 87 | 90 | 93 | 97 | 101 | 105 |
| 270 | 84 | 87 | 90 | 94 | 97 | 101 | 105 |
| 271 | 84 | 87 | 90 | 94 | 97 | 101 | 105 |
| 272 | 84 | 87 | 91 | 94 | 98 | 101 | 105 |
| 273 | 84 | 87 | 91 | 94 | 98 | 102 | 105 |
| 274 | 84 | 87 | 91 | 94 | 98 | 102 | 106 |
| 275 | 84 | 88 | 91 | 95 | 98 | 102 | 106 |
| 276 | 85 | 88 | 91 | 95 | 98 | 102 | 106 |
| 277 | 85 | 88 | 91 | 95 | 99 | 102 | 106 |
| 278 | 85 | 88 | 91 | 95 | 99 | 103 | 106 |
| 279 | 85 | 88 | 92 | 95 | 99 | 103 | 107 |
| 280 | 85 | 88 | 92 | 95 | 99 | 103 | 107 |
| 281 | 85 | 88 | 92 | 95 | 99 | 103 | 107 |
| 282 | 85 | 89 | 92 | 96 | 99 | 103 | 107 |
| 283 | 85 | 89 | 92 | 96 | 99 | 103 | 107 |
| 284 | 85 | 89 | 92 | 96 | 100 | 104 | 108 |
| 285 | 85 | 89 | 92 | 96 | 100 | 104 | 108 |
| 286 | 86 | 89 | 92 | 96 | 100 | 104 | 108 |
| 287 | 86 | 89 | 93 | 96 | 100 | 104 | 108 |
| 288 | 86 | 89 | 93 | 96 | 100 | 104 | 108 |
| 289 | 86 | 89 | 93 | 97 | 100 | 104 | 109 |
| 290 | 86 | 89 | 93 | 97 | 101 | 105 | 109 |
| 291 | 86 | 89 | 93 | 97 | 101 | 105 | 109 |
| 292 | 86 | 90 | 93 | 97 | 101 | 105 | 109 |
| 293 | 86 | 90 | 93 | 97 | 101 | 105 | 109 |
| 294 | 86 | 90 | 93 | 97 | 101 | 105 | 109 |

Mean and variance equation for BPD in males and females:

*E(Z*_i_) = -2.529176209942673 + [2.473072527732841 log(GA_i_)] + [-0.0509174652560741 GA_i_^1^]

*Var(Z*_i_) = 0.0819841080152605 + [0.0200671890593554 log(GA_i_)^2^] + [-0.0807247079472814 log(GA_i_)] + [0.0032704019422098 GA_i_^1^] + [-0.0008227067786255 log(GA_i_)GA_i_^1^] + [0.0000351017588443 GA_i_^2^]

Supplementary Table 6b. Estimated biparietal diameter (BPD) in mm by gestational age (GA) for males and females, percentiles.

| GA (days) | 2.5^th^ | 5^th^ | 10^th^ | 25^th^ | Median | 75^th^ | 90^th^ | 95^th^ | 97.5^th^ |
| --- | --- | --- | --- | --- | --- | --- | --- | --- | --- |
| 84 | 19 | 19 | 20 | 20 | 20 | 21 | 21 | 21 | 21 |
| 85 | 20 | 20 | 20 | 20 | 21 | 21 | 21 | 21 | 22 |
| 86 | 20 | 20 | 20 | 21 | 21 | 21 | 22 | 22 | 22 |
| 87 | 21 | 21 | 21 | 21 | 22 | 22 | 22 | 22 | 23 |
| 88 | 21 | 21 | 21 | 22 | 22 | 22 | 23 | 23 | 23 |
| 89 | 21 | 22 | 22 | 22 | 22 | 23 | 23 | 23 | 23 |
| 90 | 22 | 22 | 22 | 23 | 23 | 23 | 24 | 24 | 24 |
| 91 | 22 | 23 | 23 | 23 | 23 | 24 | 24 | 24 | 24 |
| 92 | 23 | 23 | 23 | 23 | 24 | 24 | 25 | 25 | 25 |
| 93 | 23 | 23 | 24 | 24 | 24 | 25 | 25 | 25 | 25 |
| 94 | 24 | 24 | 24 | 24 | 25 | 25 | 26 | 26 | 26 |
| 95 | 24 | 24 | 25 | 25 | 25 | 26 | 26 | 26 | 26 |
| 96 | 25 | 25 | 25 | 25 | 26 | 26 | 27 | 27 | 27 |
| 97 | 25 | 25 | 25 | 26 | 26 | 27 | 27 | 27 | 27 |
| 98 | 25 | 26 | 26 | 26 | 27 | 27 | 28 | 28 | 28 |
| 99 | 26 | 26 | 26 | 27 | 27 | 28 | 28 | 28 | 29 |
| 100 | 26 | 27 | 27 | 27 | 28 | 28 | 29 | 29 | 29 |
| 101 | 27 | 27 | 27 | 28 | 28 | 29 | 29 | 29 | 30 |
| 102 | 27 | 27 | 28 | 28 | 29 | 29 | 30 | 30 | 30 |
| 103 | 28 | 28 | 28 | 29 | 29 | 30 | 30 | 30 | 31 |
| 104 | 28 | 28 | 29 | 29 | 30 | 30 | 31 | 31 | 31 |
| 105 | 29 | 29 | 29 | 30 | 30 | 31 | 31 | 31 | 32 |
| 106 | 29 | 29 | 30 | 30 | 31 | 31 | 32 | 32 | 32 |
| 107 | 30 | 30 | 30 | 31 | 31 | 32 | 32 | 32 | 33 |
| 108 | 30 | 30 | 31 | 31 | 32 | 32 | 33 | 33 | 33 |
| 109 | 30 | 31 | 31 | 31 | 32 | 33 | 33 | 33 | 34 |
| 110 | 31 | 31 | 31 | 32 | 33 | 33 | 34 | 34 | 34 |
| 111 | 31 | 32 | 32 | 32 | 33 | 34 | 34 | 35 | 35 |
| 112 | 32 | 32 | 32 | 33 | 34 | 34 | 35 | 35 | 35 |
| 113 | 32 | 33 | 33 | 33 | 34 | 35 | 35 | 36 | 36 |
| 114 | 33 | 33 | 33 | 34 | 35 | 35 | 36 | 36 | 36 |
| 115 | 33 | 33 | 34 | 34 | 35 | 36 | 36 | 37 | 37 |
| 116 | 34 | 34 | 34 | 35 | 36 | 36 | 37 | 37 | 38 |
| 117 | 34 | 34 | 35 | 35 | 36 | 37 | 37 | 38 | 38 |
| 118 | 35 | 35 | 35 | 36 | 37 | 37 | 38 | 38 | 39 |
| 119 | 35 | 35 | 36 | 36 | 37 | 38 | 38 | 39 | 39 |
| 120 | 35 | 36 | 36 | 37 | 38 | 38 | 39 | 39 | 40 |
| 121 | 36 | 36 | 37 | 37 | 38 | 39 | 40 | 40 | 40 |
| 122 | 36 | 37 | 37 | 38 | 39 | 39 | 40 | 40 | 41 |
| 123 | 37 | 37 | 38 | 38 | 39 | 40 | 41 | 41 | 41 |
| 124 | 37 | 38 | 38 | 39 | 40 | 40 | 41 | 42 | 42 |
| 125 | 38 | 38 | 39 | 39 | 40 | 41 | 42 | 42 | 43 |
| 126 | 38 | 39 | 39 | 40 | 41 | 41 | 42 | 43 | 43 |
| 127 | 39 | 39 | 39 | 40 | 41 | 42 | 43 | 43 | 44 |
| 128 | 39 | 39 | 40 | 41 | 42 | 42 | 43 | 44 | 44 |
| 129 | 40 | 40 | 40 | 41 | 42 | 43 | 44 | 44 | 45 |
| 130 | 40 | 40 | 41 | 42 | 43 | 43 | 44 | 45 | 45 |
| 131 | 40 | 41 | 41 | 42 | 43 | 44 | 45 | 45 | 46 |
| 132 | 41 | 41 | 42 | 43 | 44 | 44 | 45 | 46 | 46 |
| 133 | 41 | 42 | 42 | 43 | 44 | 45 | 46 | 46 | 47 |
| 134 | 42 | 42 | 43 | 44 | 45 | 46 | 46 | 47 | 47 |
| 135 | 42 | 43 | 43 | 44 | 45 | 46 | 47 | 48 | 48 |
| 136 | 43 | 43 | 44 | 45 | 46 | 47 | 47 | 48 | 49 |
| 137 | 43 | 44 | 44 | 45 | 46 | 47 | 48 | 49 | 49 |
| 138 | 44 | 44 | 45 | 46 | 47 | 48 | 49 | 49 | 50 |
| 139 | 44 | 45 | 45 | 46 | 47 | 48 | 49 | 50 | 50 |
| 140 | 45 | 45 | 46 | 46 | 48 | 49 | 50 | 50 | 51 |
| 141 | 45 | 45 | 46 | 47 | 48 | 49 | 50 | 51 | 51 |
| 142 | 45 | 46 | 46 | 47 | 49 | 50 | 51 | 51 | 52 |
| 143 | 46 | 46 | 47 | 48 | 49 | 50 | 51 | 52 | 52 |
| 144 | 46 | 47 | 47 | 48 | 49 | 51 | 52 | 52 | 53 |
| 145 | 47 | 47 | 48 | 49 | 50 | 51 | 52 | 53 | 53 |
| 146 | 47 | 48 | 48 | 49 | 50 | 52 | 53 | 53 | 54 |
| 147 | 48 | 48 | 49 | 50 | 51 | 52 | 53 | 54 | 55 |
| 148 | 48 | 49 | 49 | 50 | 51 | 53 | 54 | 54 | 55 |
| 149 | 49 | 49 | 50 | 51 | 52 | 53 | 54 | 55 | 56 |
| 150 | 49 | 49 | 50 | 51 | 52 | 54 | 55 | 55 | 56 |
| 151 | 49 | 50 | 51 | 52 | 53 | 54 | 55 | 56 | 57 |
| 152 | 50 | 50 | 51 | 52 | 53 | 55 | 56 | 57 | 57 |
| 153 | 50 | 51 | 51 | 53 | 54 | 55 | 56 | 57 | 58 |
| 154 | 51 | 51 | 52 | 53 | 54 | 56 | 57 | 58 | 58 |
| 155 | 51 | 52 | 52 | 54 | 55 | 56 | 57 | 58 | 59 |
| 156 | 52 | 52 | 53 | 54 | 55 | 57 | 58 | 59 | 59 |
| 157 | 52 | 53 | 53 | 54 | 56 | 57 | 58 | 59 | 60 |
| 158 | 52 | 53 | 54 | 55 | 56 | 58 | 59 | 60 | 60 |
| 159 | 53 | 53 | 54 | 55 | 57 | 58 | 59 | 60 | 61 |
| 160 | 53 | 54 | 55 | 56 | 57 | 59 | 60 | 61 | 61 |
| 161 | 54 | 54 | 55 | 56 | 58 | 59 | 60 | 61 | 62 |
| 162 | 54 | 55 | 55 | 57 | 58 | 60 | 61 | 62 | 62 |
| 163 | 55 | 55 | 56 | 57 | 59 | 60 | 61 | 62 | 63 |
| 164 | 55 | 56 | 56 | 58 | 59 | 60 | 62 | 63 | 63 |
| 165 | 55 | 56 | 57 | 58 | 59 | 61 | 62 | 63 | 64 |
| 166 | 56 | 56 | 57 | 58 | 60 | 61 | 63 | 64 | 64 |
| 167 | 56 | 57 | 58 | 59 | 60 | 62 | 63 | 64 | 65 |
| 168 | 57 | 57 | 58 | 59 | 61 | 62 | 64 | 65 | 65 |
| 169 | 57 | 58 | 59 | 60 | 61 | 63 | 64 | 65 | 66 |
| 170 | 57 | 58 | 59 | 60 | 62 | 63 | 65 | 66 | 66 |
| 171 | 58 | 59 | 59 | 61 | 62 | 64 | 65 | 66 | 67 |
| 172 | 58 | 59 | 60 | 61 | 63 | 64 | 66 | 67 | 67 |
| 173 | 59 | 59 | 60 | 62 | 63 | 65 | 66 | 67 | 68 |
| 174 | 59 | 60 | 61 | 62 | 64 | 65 | 67 | 67 | 68 |
| 175 | 60 | 60 | 61 | 62 | 64 | 66 | 67 | 68 | 69 |
| 176 | 60 | 61 | 61 | 63 | 64 | 66 | 68 | 68 | 69 |
| 177 | 60 | 61 | 62 | 63 | 65 | 66 | 68 | 69 | 70 |
| 178 | 61 | 61 | 62 | 64 | 65 | 67 | 68 | 69 | 70 |
| 179 | 61 | 62 | 63 | 64 | 66 | 67 | 69 | 70 | 71 |
| 180 | 62 | 62 | 63 | 65 | 66 | 68 | 69 | 70 | 71 |
| 181 | 62 | 63 | 63 | 65 | 67 | 68 | 70 | 71 | 72 |
| 182 | 62 | 63 | 64 | 65 | 67 | 69 | 70 | 71 | 72 |
| 183 | 63 | 63 | 64 | 66 | 67 | 69 | 71 | 72 | 72 |
| 184 | 63 | 64 | 65 | 66 | 68 | 70 | 71 | 72 | 73 |
| 185 | 63 | 64 | 65 | 67 | 68 | 70 | 72 | 73 | 73 |
| 186 | 64 | 65 | 65 | 67 | 69 | 70 | 72 | 73 | 74 |
| 187 | 64 | 65 | 66 | 67 | 69 | 71 | 72 | 73 | 74 |
| 188 | 65 | 65 | 66 | 68 | 69 | 71 | 73 | 74 | 75 |
| 189 | 65 | 66 | 67 | 68 | 70 | 72 | 73 | 74 | 75 |
| 190 | 65 | 66 | 67 | 69 | 70 | 72 | 74 | 75 | 76 |
| 191 | 66 | 67 | 67 | 69 | 71 | 72 | 74 | 75 | 76 |
| 192 | 66 | 67 | 68 | 69 | 71 | 73 | 75 | 76 | 76 |
| 193 | 66 | 67 | 68 | 70 | 71 | 73 | 75 | 76 | 77 |
| 194 | 67 | 68 | 69 | 70 | 72 | 74 | 75 | 76 | 77 |
| 195 | 67 | 68 | 69 | 70 | 72 | 74 | 76 | 77 | 78 |
| 196 | 68 | 68 | 69 | 71 | 73 | 75 | 76 | 77 | 78 |
| 197 | 68 | 69 | 70 | 71 | 73 | 75 | 77 | 78 | 79 |
| 198 | 68 | 69 | 70 | 72 | 73 | 75 | 77 | 78 | 79 |
| 199 | 69 | 69 | 70 | 72 | 74 | 76 | 77 | 78 | 79 |
| 200 | 69 | 70 | 71 | 72 | 74 | 76 | 78 | 79 | 80 |
| 201 | 69 | 70 | 71 | 73 | 75 | 76 | 78 | 79 | 80 |
| 202 | 70 | 71 | 71 | 73 | 75 | 77 | 79 | 80 | 81 |
| 203 | 70 | 71 | 72 | 73 | 75 | 77 | 79 | 80 | 81 |
| 204 | 70 | 71 | 72 | 74 | 76 | 78 | 79 | 80 | 81 |
| 205 | 71 | 72 | 73 | 74 | 76 | 78 | 80 | 81 | 82 |
| 206 | 71 | 72 | 73 | 75 | 76 | 78 | 80 | 81 | 82 |
| 207 | 71 | 72 | 73 | 75 | 77 | 79 | 81 | 82 | 83 |
| 208 | 72 | 73 | 74 | 75 | 77 | 79 | 81 | 82 | 83 |
| 209 | 72 | 73 | 74 | 76 | 77 | 79 | 81 | 82 | 83 |
| 210 | 72 | 73 | 74 | 76 | 78 | 80 | 82 | 83 | 84 |
| 211 | 73 | 74 | 75 | 76 | 78 | 80 | 82 | 83 | 84 |
| 212 | 73 | 74 | 75 | 77 | 79 | 81 | 82 | 83 | 84 |
| 213 | 73 | 74 | 75 | 77 | 79 | 81 | 83 | 84 | 85 |
| 214 | 74 | 75 | 76 | 77 | 79 | 81 | 83 | 84 | 85 |
| 215 | 74 | 75 | 76 | 78 | 80 | 82 | 83 | 85 | 86 |
| 216 | 74 | 75 | 76 | 78 | 80 | 82 | 84 | 85 | 86 |
| 217 | 75 | 75 | 77 | 78 | 80 | 82 | 84 | 85 | 86 |
| 218 | 75 | 76 | 77 | 79 | 81 | 83 | 84 | 86 | 87 |
| 219 | 75 | 76 | 77 | 79 | 81 | 83 | 85 | 86 | 87 |
| 220 | 76 | 76 | 77 | 79 | 81 | 83 | 85 | 86 | 87 |
| 221 | 76 | 77 | 78 | 80 | 82 | 84 | 85 | 87 | 88 |
| 222 | 76 | 77 | 78 | 80 | 82 | 84 | 86 | 87 | 88 |
| 223 | 76 | 77 | 78 | 80 | 82 | 84 | 86 | 87 | 88 |
| 224 | 77 | 78 | 79 | 80 | 82 | 85 | 86 | 88 | 89 |
| 225 | 77 | 78 | 79 | 81 | 83 | 85 | 87 | 88 | 89 |
| 226 | 77 | 78 | 79 | 81 | 83 | 85 | 87 | 88 | 89 |
| 227 | 78 | 78 | 80 | 81 | 83 | 85 | 87 | 89 | 90 |
| 228 | 78 | 79 | 80 | 82 | 84 | 86 | 88 | 89 | 90 |
| 229 | 78 | 79 | 80 | 82 | 84 | 86 | 88 | 89 | 90 |
| 230 | 78 | 79 | 80 | 82 | 84 | 86 | 88 | 90 | 91 |
| 231 | 79 | 80 | 81 | 83 | 85 | 87 | 89 | 90 | 91 |
| 232 | 79 | 80 | 81 | 83 | 85 | 87 | 89 | 90 | 91 |
| 233 | 79 | 80 | 81 | 83 | 85 | 87 | 89 | 90 | 92 |
| 234 | 79 | 80 | 81 | 83 | 85 | 88 | 90 | 91 | 92 |
| 235 | 80 | 81 | 82 | 84 | 86 | 88 | 90 | 91 | 92 |
| 236 | 80 | 81 | 82 | 84 | 86 | 88 | 90 | 91 | 92 |
| 237 | 80 | 81 | 82 | 84 | 86 | 88 | 90 | 92 | 93 |
| 238 | 81 | 81 | 83 | 84 | 87 | 89 | 91 | 92 | 93 |
| 239 | 81 | 82 | 83 | 85 | 87 | 89 | 91 | 92 | 93 |
| 240 | 81 | 82 | 83 | 85 | 87 | 89 | 91 | 92 | 94 |
| 241 | 81 | 82 | 83 | 85 | 87 | 90 | 92 | 93 | 94 |
| 242 | 82 | 82 | 84 | 85 | 88 | 90 | 92 | 93 | 94 |
| 243 | 82 | 83 | 84 | 86 | 88 | 90 | 92 | 93 | 94 |
| 244 | 82 | 83 | 84 | 86 | 88 | 90 | 92 | 94 | 95 |
| 245 | 82 | 83 | 84 | 86 | 88 | 91 | 93 | 94 | 95 |
| 246 | 82 | 83 | 85 | 86 | 89 | 91 | 93 | 94 | 95 |
| 247 | 83 | 84 | 85 | 87 | 89 | 91 | 93 | 94 | 95 |
| 248 | 83 | 84 | 85 | 87 | 89 | 91 | 93 | 95 | 96 |
| 249 | 83 | 84 | 85 | 87 | 89 | 92 | 94 | 95 | 96 |
| 250 | 83 | 84 | 85 | 87 | 90 | 92 | 94 | 95 | 96 |
| 251 | 84 | 85 | 86 | 88 | 90 | 92 | 94 | 95 | 97 |
| 252 | 84 | 85 | 86 | 88 | 90 | 92 | 94 | 96 | 97 |
| 253 | 84 | 85 | 86 | 88 | 90 | 93 | 95 | 96 | 97 |
| 254 | 84 | 85 | 86 | 88 | 90 | 93 | 95 | 96 | 97 |
| 255 | 84 | 85 | 87 | 88 | 91 | 93 | 95 | 96 | 98 |
| 256 | 85 | 86 | 87 | 89 | 91 | 93 | 95 | 97 | 98 |
| 257 | 85 | 86 | 87 | 89 | 91 | 93 | 96 | 97 | 98 |
| 258 | 85 | 86 | 87 | 89 | 91 | 94 | 96 | 97 | 98 |
| 259 | 85 | 86 | 87 | 89 | 92 | 94 | 96 | 97 | 98 |
| 260 | 85 | 86 | 88 | 89 | 92 | 94 | 96 | 98 | 99 |
| 261 | 86 | 87 | 88 | 90 | 92 | 94 | 96 | 98 | 99 |
| 262 | 86 | 87 | 88 | 90 | 92 | 95 | 97 | 98 | 99 |
| 263 | 86 | 87 | 88 | 90 | 92 | 95 | 97 | 98 | 99 |
| 264 | 86 | 87 | 88 | 90 | 93 | 95 | 97 | 98 | 100 |
| 265 | 86 | 87 | 88 | 90 | 93 | 95 | 97 | 99 | 100 |
| 266 | 86 | 87 | 89 | 91 | 93 | 95 | 97 | 99 | 100 |
| 267 | 87 | 88 | 89 | 91 | 93 | 96 | 98 | 99 | 100 |
| 268 | 87 | 88 | 89 | 91 | 93 | 96 | 98 | 99 | 100 |
| 269 | 87 | 88 | 89 | 91 | 93 | 96 | 98 | 99 | 101 |
| 270 | 87 | 88 | 89 | 91 | 94 | 96 | 98 | 100 | 101 |
| 271 | 87 | 88 | 89 | 92 | 94 | 96 | 98 | 100 | 101 |
| 272 | 87 | 88 | 90 | 92 | 94 | 96 | 99 | 100 | 101 |
| 273 | 87 | 89 | 90 | 92 | 94 | 97 | 99 | 100 | 101 |
| 274 | 88 | 89 | 90 | 92 | 94 | 97 | 99 | 100 | 102 |
| 275 | 88 | 89 | 90 | 92 | 95 | 97 | 99 | 101 | 102 |
| 276 | 88 | 89 | 90 | 92 | 95 | 97 | 99 | 101 | 102 |
| 277 | 88 | 89 | 90 | 92 | 95 | 97 | 100 | 101 | 102 |
| 278 | 88 | 89 | 90 | 93 | 95 | 97 | 100 | 101 | 102 |
| 279 | 88 | 89 | 91 | 93 | 95 | 98 | 100 | 101 | 103 |
| 280 | 88 | 89 | 91 | 93 | 95 | 98 | 100 | 101 | 103 |
| 281 | 89 | 90 | 91 | 93 | 95 | 98 | 100 | 102 | 103 |
| 282 | 89 | 90 | 91 | 93 | 96 | 98 | 100 | 102 | 103 |
| 283 | 89 | 90 | 91 | 93 | 96 | 98 | 101 | 102 | 103 |
| 284 | 89 | 90 | 91 | 93 | 96 | 98 | 101 | 102 | 103 |
| 285 | 89 | 90 | 91 | 94 | 96 | 99 | 101 | 102 | 104 |
| 286 | 89 | 90 | 91 | 94 | 96 | 99 | 101 | 102 | 104 |
| 287 | 89 | 90 | 92 | 94 | 96 | 99 | 101 | 103 | 104 |
| 288 | 89 | 90 | 92 | 94 | 96 | 99 | 101 | 103 | 104 |
| 289 | 89 | 91 | 92 | 94 | 97 | 99 | 101 | 103 | 104 |
| 290 | 89 | 91 | 92 | 94 | 97 | 99 | 102 | 103 | 104 |
| 291 | 90 | 91 | 92 | 94 | 97 | 99 | 102 | 103 | 105 |
| 292 | 90 | 91 | 92 | 94 | 97 | 99 | 102 | 103 | 105 |
| 293 | 90 | 91 | 92 | 94 | 97 | 100 | 102 | 104 | 105 |
| 294 | 90 | 91 | 92 | 95 | 97 | 100 | 102 | 104 | 105 |

Mean and variance equation for BPD in males and females:

*E(Z*_i_) = -2.529176209942673 + [2.473072527732841 log(GA_i_)] + [-0.0509174652560741 GA_i_^1^]

*Var(Z*_i_) = 0.0819841080152605 + [0.0200671890593554 log(GA_i_)^2^] + [-0.0807247079472814 log(GA_i_)] + [0.0032704019422098 GA_i_^1^] + [-0.0008227067786255 log(GA_i_)GA_i_^1^] + [0.0000351017588443 GA_i_^2^]
