# Supplementary material for: Swedish intrauterine growth reference ranges of biometric measurements of fetal head, abdomen and femur
Source: Sci Rep. 2020 Dec 31;10:22441. doi: 10.1038/s41598-020-79797-8 (PMC7775468; doi:10.1038/s41598-020-79797-8)
Supplement: Supplementary file 7 — Supplementary Table 7. [file 41598_2020_79797_MOESM7_ESM.docx]

Supplementary Table 7a. Estimated head circumference (HC) in mm by gestational age (GA) for males and females, Standard deviations (SD).

| GA (days) | -3 SD | -2 SD | -1 SD | Median | +1 SD | +2 SD | +3 SD |
| --- | --- | --- | --- | --- | --- | --- | --- |
| 84 | 66 | 69 | 71 | 74 | 76 | 79 | 82 |
| 85 | 68 | 70 | 73 | 75 | 78 | 81 | 83 |
| 86 | 70 | 72 | 74 | 77 | 80 | 82 | 85 |
| 87 | 71 | 74 | 76 | 79 | 81 | 84 | 87 |
| 88 | 73 | 75 | 78 | 81 | 83 | 86 | 89 |
| 89 | 75 | 77 | 80 | 82 | 85 | 88 | 91 |
| 90 | 76 | 79 | 82 | 84 | 87 | 90 | 93 |
| 91 | 78 | 81 | 83 | 86 | 89 | 92 | 95 |
| 92 | 80 | 82 | 85 | 88 | 91 | 94 | 97 |
| 93 | 82 | 84 | 87 | 90 | 92 | 95 | 98 |
| 94 | 83 | 86 | 89 | 91 | 94 | 97 | 100 |
| 95 | 85 | 88 | 90 | 93 | 96 | 99 | 102 |
| 96 | 87 | 89 | 92 | 95 | 98 | 101 | 104 |
| 97 | 88 | 91 | 94 | 97 | 100 | 103 | 106 |
| 98 | 90 | 93 | 96 | 99 | 102 | 105 | 108 |
| 99 | 92 | 95 | 98 | 101 | 104 | 107 | 110 |
| 100 | 94 | 96 | 99 | 102 | 106 | 109 | 112 |
| 101 | 95 | 98 | 101 | 104 | 107 | 111 | 114 |
| 102 | 97 | 100 | 103 | 106 | 109 | 113 | 116 |
| 103 | 99 | 102 | 105 | 108 | 111 | 115 | 118 |
| 104 | 101 | 104 | 107 | 110 | 113 | 117 | 120 |
| 105 | 102 | 105 | 108 | 112 | 115 | 118 | 122 |
| 106 | 104 | 107 | 110 | 114 | 117 | 120 | 124 |
| 107 | 106 | 109 | 112 | 115 | 119 | 122 | 126 |
| 108 | 107 | 111 | 114 | 117 | 121 | 124 | 128 |
| 109 | 109 | 112 | 116 | 119 | 123 | 126 | 130 |
| 110 | 111 | 114 | 118 | 121 | 125 | 128 | 132 |
| 111 | 113 | 116 | 119 | 123 | 127 | 130 | 134 |
| 112 | 114 | 118 | 121 | 125 | 128 | 132 | 136 |
| 113 | 116 | 119 | 123 | 127 | 130 | 134 | 138 |
| 114 | 118 | 121 | 125 | 128 | 132 | 136 | 140 |
| 115 | 120 | 123 | 127 | 130 | 134 | 138 | 142 |
| 116 | 121 | 125 | 128 | 132 | 136 | 140 | 144 |
| 117 | 123 | 127 | 130 | 134 | 138 | 142 | 146 |
| 118 | 125 | 128 | 132 | 136 | 140 | 144 | 148 |
| 119 | 126 | 130 | 134 | 138 | 142 | 146 | 150 |
| 120 | 128 | 132 | 136 | 140 | 144 | 148 | 152 |
| 121 | 130 | 134 | 137 | 142 | 146 | 150 | 154 |
| 122 | 131 | 135 | 139 | 143 | 148 | 152 | 156 |
| 123 | 133 | 137 | 141 | 145 | 149 | 154 | 158 |
| 124 | 135 | 139 | 143 | 147 | 151 | 156 | 160 |
| 125 | 137 | 141 | 145 | 149 | 153 | 158 | 162 |
| 126 | 138 | 142 | 146 | 151 | 155 | 160 | 164 |
| 127 | 140 | 144 | 148 | 153 | 157 | 162 | 166 |
| 128 | 142 | 146 | 150 | 154 | 159 | 164 | 168 |
| 129 | 143 | 147 | 152 | 156 | 161 | 166 | 170 |
| 130 | 145 | 149 | 154 | 158 | 163 | 168 | 172 |
| 131 | 147 | 151 | 155 | 160 | 165 | 169 | 175 |
| 132 | 148 | 153 | 157 | 162 | 167 | 171 | 177 |
| 133 | 150 | 154 | 159 | 164 | 168 | 173 | 179 |
| 134 | 151 | 156 | 161 | 165 | 170 | 175 | 181 |
| 135 | 153 | 158 | 162 | 167 | 172 | 177 | 183 |
| 136 | 155 | 159 | 164 | 169 | 174 | 179 | 185 |
| 137 | 156 | 161 | 166 | 171 | 176 | 181 | 187 |
| 138 | 158 | 163 | 168 | 173 | 178 | 183 | 189 |
| 139 | 160 | 164 | 169 | 174 | 180 | 185 | 190 |
| 140 | 161 | 166 | 171 | 176 | 181 | 187 | 192 |
| 141 | 163 | 168 | 173 | 178 | 183 | 189 | 194 |
| 142 | 164 | 169 | 174 | 180 | 185 | 191 | 196 |
| 143 | 166 | 171 | 176 | 181 | 187 | 193 | 198 |
| 144 | 168 | 173 | 178 | 183 | 189 | 194 | 200 |
| 145 | 169 | 174 | 180 | 185 | 191 | 196 | 202 |
| 146 | 171 | 176 | 181 | 187 | 192 | 198 | 204 |
| 147 | 172 | 178 | 183 | 188 | 194 | 200 | 206 |
| 148 | 174 | 179 | 185 | 190 | 196 | 202 | 208 |
| 149 | 175 | 181 | 186 | 192 | 198 | 204 | 210 |
| 150 | 177 | 182 | 188 | 194 | 200 | 206 | 212 |
| 151 | 179 | 184 | 190 | 195 | 201 | 208 | 214 |
| 152 | 180 | 186 | 191 | 197 | 203 | 209 | 216 |
| 153 | 182 | 187 | 193 | 199 | 205 | 211 | 218 |
| 154 | 183 | 189 | 195 | 201 | 207 | 213 | 220 |
| 155 | 185 | 190 | 196 | 202 | 208 | 215 | 222 |
| 156 | 186 | 192 | 198 | 204 | 210 | 217 | 223 |
| 157 | 188 | 193 | 199 | 206 | 212 | 219 | 225 |
| 158 | 189 | 195 | 201 | 207 | 214 | 220 | 227 |
| 159 | 191 | 197 | 203 | 209 | 215 | 222 | 229 |
| 160 | 192 | 198 | 204 | 211 | 217 | 224 | 231 |
| 161 | 194 | 200 | 206 | 212 | 219 | 226 | 233 |
| 162 | 195 | 201 | 207 | 214 | 221 | 227 | 235 |
| 163 | 196 | 203 | 209 | 215 | 222 | 229 | 236 |
| 164 | 198 | 204 | 211 | 217 | 224 | 231 | 238 |
| 165 | 199 | 206 | 212 | 219 | 226 | 233 | 240 |
| 166 | 201 | 207 | 214 | 220 | 227 | 234 | 242 |
| 167 | 202 | 209 | 215 | 222 | 229 | 236 | 244 |
| 168 | 204 | 210 | 217 | 224 | 231 | 238 | 245 |
| 169 | 205 | 212 | 218 | 225 | 232 | 240 | 247 |
| 170 | 206 | 213 | 220 | 227 | 234 | 241 | 249 |
| 171 | 208 | 214 | 221 | 228 | 236 | 243 | 251 |
| 172 | 209 | 216 | 223 | 230 | 237 | 245 | 253 |
| 173 | 211 | 217 | 224 | 231 | 239 | 246 | 254 |
| 174 | 212 | 219 | 226 | 233 | 240 | 248 | 256 |
| 175 | 213 | 220 | 227 | 234 | 242 | 250 | 258 |
| 176 | 215 | 222 | 229 | 236 | 244 | 251 | 259 |
| 177 | 216 | 223 | 230 | 238 | 245 | 253 | 261 |
| 178 | 217 | 224 | 232 | 239 | 247 | 255 | 263 |
| 179 | 219 | 226 | 233 | 241 | 248 | 256 | 265 |
| 180 | 220 | 227 | 234 | 242 | 250 | 258 | 266 |
| 181 | 221 | 228 | 236 | 243 | 251 | 259 | 268 |
| 182 | 223 | 230 | 237 | 245 | 253 | 261 | 270 |
| 183 | 224 | 231 | 239 | 246 | 254 | 263 | 271 |
| 184 | 225 | 233 | 240 | 248 | 256 | 264 | 273 |
| 185 | 226 | 234 | 241 | 249 | 257 | 266 | 274 |
| 186 | 228 | 235 | 243 | 251 | 259 | 267 | 276 |
| 187 | 229 | 236 | 244 | 252 | 260 | 269 | 278 |
| 188 | 230 | 238 | 246 | 254 | 262 | 270 | 279 |
| 189 | 231 | 239 | 247 | 255 | 263 | 272 | 281 |
| 190 | 233 | 240 | 248 | 256 | 265 | 273 | 282 |
| 191 | 234 | 242 | 250 | 258 | 266 | 275 | 284 |
| 192 | 235 | 243 | 251 | 259 | 268 | 276 | 286 |
| 193 | 236 | 244 | 252 | 261 | 269 | 278 | 287 |
| 194 | 238 | 245 | 253 | 262 | 271 | 279 | 289 |
| 195 | 239 | 247 | 255 | 263 | 272 | 281 | 290 |
| 196 | 240 | 248 | 256 | 265 | 273 | 282 | 292 |
| 197 | 241 | 249 | 257 | 266 | 275 | 284 | 293 |
| 198 | 242 | 250 | 259 | 267 | 276 | 285 | 295 |
| 199 | 243 | 251 | 260 | 269 | 277 | 287 | 296 |
| 200 | 245 | 253 | 261 | 270 | 279 | 288 | 298 |
| 201 | 246 | 254 | 262 | 271 | 280 | 289 | 299 |
| 202 | 247 | 255 | 264 | 272 | 281 | 291 | 301 |
| 203 | 248 | 256 | 265 | 274 | 283 | 292 | 302 |
| 204 | 249 | 257 | 266 | 275 | 284 | 294 | 303 |
| 205 | 250 | 258 | 267 | 276 | 285 | 295 | 305 |
| 206 | 251 | 260 | 268 | 277 | 287 | 296 | 306 |
| 207 | 252 | 261 | 270 | 279 | 288 | 298 | 308 |
| 208 | 253 | 262 | 271 | 280 | 289 | 299 | 309 |
| 209 | 254 | 263 | 272 | 281 | 291 | 300 | 310 |
| 210 | 255 | 264 | 273 | 282 | 292 | 302 | 312 |
| 211 | 256 | 265 | 274 | 283 | 293 | 303 | 313 |
| 212 | 257 | 266 | 275 | 285 | 294 | 304 | 315 |
| 213 | 258 | 267 | 276 | 286 | 295 | 306 | 316 |
| 214 | 259 | 268 | 277 | 287 | 297 | 307 | 317 |
| 215 | 260 | 269 | 279 | 288 | 298 | 308 | 319 |
| 216 | 261 | 270 | 280 | 289 | 299 | 309 | 320 |
| 217 | 262 | 271 | 281 | 290 | 300 | 311 | 321 |
| 218 | 263 | 272 | 282 | 291 | 301 | 312 | 322 |
| 219 | 264 | 273 | 283 | 293 | 303 | 313 | 324 |
| 220 | 265 | 274 | 284 | 294 | 304 | 314 | 325 |
| 221 | 266 | 275 | 285 | 295 | 305 | 315 | 326 |
| 222 | 267 | 276 | 286 | 296 | 306 | 317 | 328 |
| 223 | 268 | 277 | 287 | 297 | 307 | 318 | 329 |
| 224 | 269 | 278 | 288 | 298 | 308 | 319 | 330 |
| 225 | 270 | 279 | 289 | 299 | 309 | 320 | 331 |
| 226 | 271 | 280 | 290 | 300 | 310 | 321 | 332 |
| 227 | 272 | 281 | 291 | 301 | 311 | 322 | 334 |
| 228 | 272 | 282 | 292 | 302 | 313 | 323 | 335 |
| 229 | 273 | 283 | 293 | 303 | 314 | 325 | 336 |
| 230 | 274 | 284 | 294 | 304 | 315 | 326 | 337 |
| 231 | 275 | 285 | 295 | 305 | 316 | 327 | 338 |
| 232 | 276 | 286 | 296 | 306 | 317 | 328 | 339 |
| 233 | 277 | 286 | 296 | 307 | 318 | 329 | 341 |
| 234 | 277 | 287 | 297 | 308 | 319 | 330 | 342 |
| 235 | 278 | 288 | 298 | 309 | 320 | 331 | 343 |
| 236 | 279 | 289 | 299 | 310 | 321 | 332 | 344 |
| 237 | 280 | 290 | 300 | 311 | 322 | 333 | 345 |
| 238 | 280 | 290 | 301 | 312 | 323 | 334 | 346 |
| 239 | 281 | 291 | 302 | 312 | 324 | 335 | 347 |
| 240 | 282 | 292 | 302 | 313 | 325 | 336 | 348 |
| 241 | 283 | 293 | 303 | 314 | 325 | 337 | 349 |
| 242 | 283 | 294 | 304 | 315 | 326 | 338 | 350 |
| 243 | 284 | 294 | 305 | 316 | 327 | 339 | 351 |
| 244 | 285 | 295 | 306 | 317 | 328 | 340 | 352 |
| 245 | 285 | 296 | 306 | 318 | 329 | 341 | 353 |
| 246 | 286 | 296 | 307 | 318 | 330 | 342 | 354 |
| 247 | 287 | 297 | 308 | 319 | 331 | 343 | 355 |
| 248 | 287 | 298 | 309 | 320 | 332 | 344 | 356 |
| 249 | 288 | 299 | 309 | 321 | 333 | 345 | 357 |
| 250 | 289 | 299 | 310 | 322 | 333 | 346 | 358 |
| 251 | 289 | 300 | 311 | 322 | 334 | 346 | 359 |
| 252 | 290 | 301 | 312 | 323 | 335 | 347 | 360 |
| 253 | 290 | 301 | 312 | 324 | 336 | 348 | 361 |
| 254 | 291 | 302 | 313 | 325 | 337 | 349 | 362 |
| 255 | 292 | 302 | 314 | 325 | 337 | 350 | 363 |
| 256 | 292 | 303 | 314 | 326 | 338 | 351 | 364 |
| 257 | 293 | 304 | 315 | 327 | 339 | 352 | 365 |
| 258 | 293 | 304 | 316 | 327 | 340 | 352 | 366 |
| 259 | 294 | 305 | 316 | 328 | 340 | 353 | 367 |
| 260 | 294 | 305 | 317 | 329 | 341 | 354 | 367 |
| 261 | 295 | 306 | 317 | 329 | 342 | 355 | 368 |
| 262 | 295 | 306 | 318 | 330 | 343 | 356 | 369 |
| 263 | 296 | 307 | 319 | 331 | 343 | 356 | 370 |
| 264 | 296 | 307 | 319 | 331 | 344 | 357 | 371 |
| 265 | 296 | 308 | 320 | 332 | 345 | 358 | 372 |
| 266 | 297 | 308 | 320 | 333 | 345 | 359 | 373 |
| 267 | 297 | 309 | 321 | 333 | 346 | 359 | 373 |
| 268 | 298 | 309 | 321 | 334 | 347 | 360 | 374 |
| 269 | 298 | 310 | 322 | 334 | 347 | 361 | 375 |
| 270 | 299 | 310 | 322 | 335 | 348 | 362 | 376 |
| 271 | 299 | 311 | 323 | 336 | 349 | 362 | 377 |
| 272 | 299 | 311 | 323 | 336 | 349 | 363 | 377 |
| 273 | 300 | 311 | 324 | 337 | 350 | 364 | 378 |
| 274 | 300 | 312 | 324 | 337 | 351 | 364 | 379 |
| 275 | 300 | 312 | 325 | 338 | 351 | 365 | 380 |
| 276 | 301 | 313 | 325 | 338 | 352 | 366 | 380 |
| 277 | 301 | 313 | 326 | 339 | 352 | 366 | 381 |
| 278 | 301 | 313 | 326 | 339 | 353 | 367 | 382 |
| 279 | 301 | 314 | 326 | 340 | 353 | 368 | 383 |
| 280 | 302 | 314 | 327 | 340 | 354 | 368 | 383 |
| 281 | 302 | 314 | 327 | 341 | 354 | 369 | 384 |
| 282 | 302 | 315 | 328 | 341 | 355 | 370 | 385 |
| 283 | 303 | 315 | 328 | 341 | 355 | 370 | 385 |
| 284 | 303 | 315 | 328 | 342 | 356 | 371 | 386 |
| 285 | 303 | 316 | 329 | 342 | 356 | 371 | 387 |
| 286 | 303 | 316 | 329 | 343 | 357 | 372 | 387 |
| 287 | 303 | 316 | 329 | 343 | 357 | 372 | 388 |
| 288 | 304 | 316 | 330 | 343 | 358 | 373 | 389 |
| 289 | 304 | 317 | 330 | 344 | 358 | 374 | 389 |
| 290 | 304 | 317 | 330 | 344 | 359 | 374 | 390 |
| 291 | 304 | 317 | 330 | 345 | 359 | 375 | 391 |
| 292 | 304 | 317 | 331 | 345 | 360 | 375 | 391 |
| 293 | 304 | 317 | 331 | 345 | 360 | 376 | 392 |
| 294 | 304 | 318 | 331 | 346 | 361 | 376 | 392 |

Mean and variance equation for HC in males and females:

*E(Z*_i_) = 8.475388695560605 + [-14.35535180588728 GA_i_^-0.5^] + [-0.0002352579455448 GA_i_^2^]

*Var(Z*_i_) = 0.0183844497195926 + [0.2541680653875657 GA_i_^-1^] + [-0.1331594083132348 GA_i_^-0.5]^ + [-8.24021018612e-06 GA_i_^2^] + [0.0000146556506322 GA_i_^-0.5^GA_i_^2^] + [1.43492118971e-09 GA_i_^4^]

Supplementary Table 7b. Estimated head circumference (HC) in mm by gestational age (GA) for males and females, percentiles.

| GA (days) | 2.5^th^ | 5^th^ | 10^th^ | 25^th^ | Median | 75^th^ | 90^th^ | 95^th^ | 97.5^th^ |
| --- | --- | --- | --- | --- | --- | --- | --- | --- | --- |
| 84 | 69 | 69 | 70 | 72 | 74 | 75 | 77 | 78 | 79 |
| 85 | 70 | 71 | 72 | 74 | 75 | 77 | 79 | 80 | 80 |
| 86 | 72 | 73 | 74 | 75 | 77 | 79 | 80 | 81 | 82 |
| 87 | 74 | 75 | 76 | 77 | 79 | 81 | 82 | 83 | 84 |
| 88 | 76 | 76 | 77 | 79 | 81 | 82 | 84 | 85 | 86 |
| 89 | 77 | 78 | 79 | 81 | 82 | 84 | 86 | 87 | 88 |
| 90 | 79 | 80 | 81 | 82 | 84 | 86 | 88 | 89 | 90 |
| 91 | 81 | 82 | 83 | 84 | 86 | 88 | 90 | 91 | 92 |
| 92 | 82 | 83 | 84 | 86 | 88 | 90 | 91 | 93 | 93 |
| 93 | 84 | 85 | 86 | 88 | 90 | 92 | 93 | 94 | 95 |
| 94 | 86 | 87 | 88 | 90 | 91 | 93 | 95 | 96 | 97 |
| 95 | 88 | 89 | 90 | 91 | 93 | 95 | 97 | 98 | 99 |
| 96 | 90 | 90 | 91 | 93 | 95 | 97 | 99 | 100 | 101 |
| 97 | 91 | 92 | 93 | 95 | 97 | 99 | 101 | 102 | 103 |
| 98 | 93 | 94 | 95 | 97 | 99 | 101 | 103 | 104 | 105 |
| 99 | 95 | 96 | 97 | 99 | 101 | 103 | 105 | 106 | 107 |
| 100 | 97 | 97 | 99 | 100 | 102 | 105 | 106 | 108 | 109 |
| 101 | 98 | 99 | 100 | 102 | 104 | 106 | 108 | 110 | 111 |
| 102 | 100 | 101 | 102 | 104 | 106 | 108 | 110 | 111 | 113 |
| 103 | 102 | 103 | 104 | 106 | 108 | 110 | 112 | 113 | 114 |
| 104 | 104 | 105 | 106 | 108 | 110 | 112 | 114 | 115 | 116 |
| 105 | 105 | 106 | 108 | 110 | 112 | 114 | 116 | 117 | 118 |
| 106 | 107 | 108 | 109 | 111 | 114 | 116 | 118 | 119 | 120 |
| 107 | 109 | 110 | 111 | 113 | 115 | 118 | 120 | 121 | 122 |
| 108 | 111 | 112 | 113 | 115 | 117 | 120 | 122 | 123 | 124 |
| 109 | 113 | 114 | 115 | 117 | 119 | 122 | 124 | 125 | 126 |
| 110 | 114 | 115 | 117 | 119 | 121 | 123 | 126 | 127 | 128 |
| 111 | 116 | 117 | 118 | 121 | 123 | 125 | 128 | 129 | 130 |
| 112 | 118 | 119 | 120 | 122 | 125 | 127 | 129 | 131 | 132 |
| 113 | 120 | 121 | 122 | 124 | 127 | 129 | 131 | 133 | 134 |
| 114 | 121 | 123 | 124 | 126 | 128 | 131 | 133 | 135 | 136 |
| 115 | 123 | 124 | 126 | 128 | 130 | 133 | 135 | 137 | 138 |
| 116 | 125 | 126 | 127 | 130 | 132 | 135 | 137 | 139 | 140 |
| 117 | 127 | 128 | 129 | 131 | 134 | 137 | 139 | 141 | 142 |
| 118 | 128 | 130 | 131 | 133 | 136 | 139 | 141 | 143 | 144 |
| 119 | 130 | 131 | 133 | 135 | 138 | 140 | 143 | 144 | 146 |
| 120 | 132 | 133 | 135 | 137 | 140 | 142 | 145 | 146 | 148 |
| 121 | 134 | 135 | 136 | 139 | 142 | 144 | 147 | 148 | 150 |
| 122 | 135 | 137 | 138 | 141 | 143 | 146 | 149 | 150 | 152 |
| 123 | 137 | 138 | 140 | 142 | 145 | 148 | 151 | 152 | 154 |
| 124 | 139 | 140 | 142 | 144 | 147 | 150 | 153 | 154 | 156 |
| 125 | 141 | 142 | 143 | 146 | 149 | 152 | 155 | 156 | 158 |
| 126 | 142 | 144 | 145 | 148 | 151 | 154 | 156 | 158 | 160 |
| 127 | 144 | 145 | 147 | 150 | 153 | 156 | 158 | 160 | 162 |
| 128 | 146 | 147 | 149 | 151 | 154 | 157 | 160 | 162 | 163 |
| 129 | 148 | 149 | 151 | 153 | 156 | 159 | 162 | 164 | 165 |
| 130 | 149 | 151 | 152 | 155 | 158 | 161 | 164 | 166 | 167 |
| 131 | 151 | 152 | 154 | 157 | 160 | 163 | 166 | 168 | 169 |
| 132 | 153 | 154 | 156 | 159 | 162 | 165 | 168 | 170 | 171 |
| 133 | 154 | 156 | 158 | 160 | 164 | 167 | 170 | 172 | 173 |
| 134 | 156 | 158 | 159 | 162 | 165 | 169 | 172 | 174 | 175 |
| 135 | 158 | 159 | 161 | 164 | 167 | 171 | 174 | 175 | 177 |
| 136 | 160 | 161 | 163 | 166 | 169 | 172 | 175 | 177 | 179 |
| 137 | 161 | 163 | 164 | 167 | 171 | 174 | 177 | 179 | 181 |
| 138 | 163 | 164 | 166 | 169 | 173 | 176 | 179 | 181 | 183 |
| 139 | 165 | 166 | 168 | 171 | 174 | 178 | 181 | 183 | 185 |
| 140 | 166 | 168 | 170 | 173 | 176 | 180 | 183 | 185 | 187 |
| 141 | 168 | 169 | 171 | 174 | 178 | 182 | 185 | 187 | 189 |
| 142 | 170 | 171 | 173 | 176 | 180 | 183 | 187 | 189 | 190 |
| 143 | 171 | 173 | 175 | 178 | 181 | 185 | 189 | 191 | 192 |
| 144 | 173 | 174 | 176 | 180 | 183 | 187 | 190 | 192 | 194 |
| 145 | 174 | 176 | 178 | 181 | 185 | 189 | 192 | 194 | 196 |
| 146 | 176 | 178 | 180 | 183 | 187 | 191 | 194 | 196 | 198 |
| 147 | 178 | 179 | 181 | 185 | 188 | 192 | 196 | 198 | 200 |
| 148 | 179 | 181 | 183 | 186 | 190 | 194 | 198 | 200 | 202 |
| 149 | 181 | 183 | 185 | 188 | 192 | 196 | 200 | 202 | 204 |
| 150 | 183 | 184 | 186 | 190 | 194 | 198 | 201 | 204 | 205 |
| 151 | 184 | 186 | 188 | 191 | 195 | 199 | 203 | 205 | 207 |
| 152 | 186 | 188 | 190 | 193 | 197 | 201 | 205 | 207 | 209 |
| 153 | 187 | 189 | 191 | 195 | 199 | 203 | 207 | 209 | 211 |
| 154 | 189 | 191 | 193 | 196 | 201 | 205 | 208 | 211 | 213 |
| 155 | 191 | 192 | 194 | 198 | 202 | 206 | 210 | 213 | 215 |
| 156 | 192 | 194 | 196 | 200 | 204 | 208 | 212 | 214 | 216 |
| 157 | 194 | 196 | 198 | 201 | 206 | 210 | 214 | 216 | 218 |
| 158 | 195 | 197 | 199 | 203 | 207 | 212 | 216 | 218 | 220 |
| 159 | 197 | 199 | 201 | 205 | 209 | 213 | 217 | 220 | 222 |
| 160 | 198 | 200 | 202 | 206 | 211 | 215 | 219 | 221 | 224 |
| 161 | 200 | 202 | 204 | 208 | 212 | 217 | 221 | 223 | 225 |
| 162 | 201 | 203 | 206 | 209 | 214 | 218 | 222 | 225 | 227 |
| 163 | 203 | 205 | 207 | 211 | 215 | 220 | 224 | 227 | 229 |
| 164 | 204 | 206 | 209 | 213 | 217 | 222 | 226 | 228 | 231 |
| 165 | 206 | 208 | 210 | 214 | 219 | 223 | 228 | 230 | 232 |
| 166 | 207 | 209 | 212 | 216 | 220 | 225 | 229 | 232 | 234 |
| 167 | 209 | 211 | 213 | 217 | 222 | 227 | 231 | 234 | 236 |
| 168 | 210 | 212 | 215 | 219 | 224 | 228 | 233 | 235 | 238 |
| 169 | 212 | 214 | 216 | 220 | 225 | 230 | 234 | 237 | 239 |
| 170 | 213 | 215 | 218 | 222 | 227 | 232 | 236 | 239 | 241 |
| 171 | 215 | 217 | 219 | 224 | 228 | 233 | 238 | 240 | 243 |
| 172 | 216 | 218 | 221 | 225 | 230 | 235 | 239 | 242 | 244 |
| 173 | 218 | 220 | 222 | 227 | 231 | 236 | 241 | 244 | 246 |
| 174 | 219 | 221 | 224 | 228 | 233 | 238 | 243 | 245 | 248 |
| 175 | 220 | 223 | 225 | 230 | 234 | 239 | 244 | 247 | 249 |
| 176 | 222 | 224 | 227 | 231 | 236 | 241 | 246 | 249 | 251 |
| 177 | 223 | 225 | 228 | 232 | 238 | 243 | 247 | 250 | 253 |
| 178 | 225 | 227 | 229 | 234 | 239 | 244 | 249 | 252 | 254 |
| 179 | 226 | 228 | 231 | 235 | 241 | 246 | 250 | 253 | 256 |
| 180 | 227 | 230 | 232 | 237 | 242 | 247 | 252 | 255 | 258 |
| 181 | 229 | 231 | 234 | 238 | 243 | 249 | 254 | 257 | 259 |
| 182 | 230 | 232 | 235 | 240 | 245 | 250 | 255 | 258 | 261 |
| 183 | 231 | 234 | 237 | 241 | 246 | 252 | 257 | 260 | 262 |
| 184 | 233 | 235 | 238 | 243 | 248 | 253 | 258 | 261 | 264 |
| 185 | 234 | 237 | 239 | 244 | 249 | 255 | 260 | 263 | 265 |
| 186 | 235 | 238 | 241 | 245 | 251 | 256 | 261 | 264 | 267 |
| 187 | 237 | 239 | 242 | 247 | 252 | 258 | 263 | 266 | 269 |
| 188 | 238 | 241 | 243 | 248 | 254 | 259 | 264 | 267 | 270 |
| 189 | 239 | 242 | 245 | 250 | 255 | 261 | 266 | 269 | 272 |
| 190 | 241 | 243 | 246 | 251 | 256 | 262 | 267 | 270 | 273 |
| 191 | 242 | 244 | 247 | 252 | 258 | 263 | 269 | 272 | 275 |
| 192 | 243 | 246 | 249 | 254 | 259 | 265 | 270 | 273 | 276 |
| 193 | 244 | 247 | 250 | 255 | 261 | 266 | 272 | 275 | 278 |
| 194 | 246 | 248 | 251 | 256 | 262 | 268 | 273 | 276 | 279 |
| 195 | 247 | 249 | 252 | 258 | 263 | 269 | 274 | 278 | 281 |
| 196 | 248 | 251 | 254 | 259 | 265 | 270 | 276 | 279 | 282 |
| 197 | 249 | 252 | 255 | 260 | 266 | 272 | 277 | 281 | 283 |
| 198 | 251 | 253 | 256 | 261 | 267 | 273 | 279 | 282 | 285 |
| 199 | 252 | 254 | 257 | 263 | 269 | 274 | 280 | 283 | 286 |
| 200 | 253 | 256 | 259 | 264 | 270 | 276 | 281 | 285 | 288 |
| 201 | 254 | 257 | 260 | 265 | 271 | 277 | 283 | 286 | 289 |
| 202 | 255 | 258 | 261 | 266 | 272 | 278 | 284 | 288 | 291 |
| 203 | 257 | 259 | 262 | 268 | 274 | 280 | 285 | 289 | 292 |
| 204 | 258 | 260 | 264 | 269 | 275 | 281 | 287 | 290 | 293 |
| 205 | 259 | 262 | 265 | 270 | 276 | 282 | 288 | 292 | 295 |
| 206 | 260 | 263 | 266 | 271 | 277 | 284 | 289 | 293 | 296 |
| 207 | 261 | 264 | 267 | 272 | 279 | 285 | 291 | 294 | 297 |
| 208 | 262 | 265 | 268 | 274 | 280 | 286 | 292 | 296 | 299 |
| 209 | 263 | 266 | 269 | 275 | 281 | 287 | 293 | 297 | 300 |
| 210 | 264 | 267 | 270 | 276 | 282 | 289 | 295 | 298 | 301 |
| 211 | 265 | 268 | 272 | 277 | 283 | 290 | 296 | 299 | 303 |
| 212 | 267 | 269 | 273 | 278 | 285 | 291 | 297 | 301 | 304 |
| 213 | 268 | 270 | 274 | 279 | 286 | 292 | 298 | 302 | 305 |
| 214 | 269 | 272 | 275 | 281 | 287 | 293 | 300 | 303 | 306 |
| 215 | 270 | 273 | 276 | 282 | 288 | 295 | 301 | 304 | 308 |
| 216 | 271 | 274 | 277 | 283 | 289 | 296 | 302 | 306 | 309 |
| 217 | 272 | 275 | 278 | 284 | 290 | 297 | 303 | 307 | 310 |
| 218 | 273 | 276 | 279 | 285 | 291 | 298 | 304 | 308 | 311 |
| 219 | 274 | 277 | 280 | 286 | 293 | 299 | 305 | 309 | 313 |
| 220 | 275 | 278 | 281 | 287 | 294 | 300 | 307 | 310 | 314 |
| 221 | 276 | 279 | 282 | 288 | 295 | 302 | 308 | 312 | 315 |
| 222 | 277 | 280 | 283 | 289 | 296 | 303 | 309 | 313 | 316 |
| 223 | 278 | 281 | 284 | 290 | 297 | 304 | 310 | 314 | 317 |
| 224 | 279 | 282 | 285 | 291 | 298 | 305 | 311 | 315 | 318 |
| 225 | 280 | 283 | 286 | 292 | 299 | 306 | 312 | 316 | 320 |
| 226 | 281 | 284 | 287 | 293 | 300 | 307 | 313 | 317 | 321 |
| 227 | 281 | 285 | 288 | 294 | 301 | 308 | 315 | 318 | 322 |
| 228 | 282 | 285 | 289 | 295 | 302 | 309 | 316 | 320 | 323 |
| 229 | 283 | 286 | 290 | 296 | 303 | 310 | 317 | 321 | 324 |
| 230 | 284 | 287 | 291 | 297 | 304 | 311 | 318 | 322 | 325 |
| 231 | 285 | 288 | 292 | 298 | 305 | 312 | 319 | 323 | 326 |
| 232 | 286 | 289 | 293 | 299 | 306 | 313 | 320 | 324 | 327 |
| 233 | 287 | 290 | 294 | 300 | 307 | 314 | 321 | 325 | 328 |
| 234 | 288 | 291 | 294 | 301 | 308 | 315 | 322 | 326 | 330 |
| 235 | 288 | 292 | 295 | 302 | 309 | 316 | 323 | 327 | 331 |
| 236 | 289 | 292 | 296 | 303 | 310 | 317 | 324 | 328 | 332 |
| 237 | 290 | 293 | 297 | 303 | 311 | 318 | 325 | 329 | 333 |
| 238 | 291 | 294 | 298 | 304 | 312 | 319 | 326 | 330 | 334 |
| 239 | 292 | 295 | 299 | 305 | 312 | 320 | 327 | 331 | 335 |
| 240 | 292 | 296 | 299 | 306 | 313 | 321 | 328 | 332 | 336 |
| 241 | 293 | 296 | 300 | 307 | 314 | 322 | 329 | 333 | 337 |
| 242 | 294 | 297 | 301 | 308 | 315 | 323 | 330 | 334 | 338 |
| 243 | 295 | 298 | 302 | 308 | 316 | 324 | 331 | 335 | 339 |
| 244 | 295 | 299 | 303 | 309 | 317 | 324 | 331 | 336 | 340 |
| 245 | 296 | 300 | 303 | 310 | 318 | 325 | 332 | 337 | 341 |
| 246 | 297 | 300 | 304 | 311 | 318 | 326 | 333 | 338 | 341 |
| 247 | 298 | 301 | 305 | 312 | 319 | 327 | 334 | 339 | 342 |
| 248 | 298 | 302 | 306 | 312 | 320 | 328 | 335 | 339 | 343 |
| 249 | 299 | 302 | 306 | 313 | 321 | 329 | 336 | 340 | 344 |
| 250 | 300 | 303 | 307 | 314 | 322 | 329 | 337 | 341 | 345 |
| 251 | 300 | 304 | 308 | 315 | 322 | 330 | 338 | 342 | 346 |
| 252 | 301 | 304 | 308 | 315 | 323 | 331 | 338 | 343 | 347 |
| 253 | 302 | 305 | 309 | 316 | 324 | 332 | 339 | 344 | 348 |
| 254 | 302 | 306 | 310 | 317 | 325 | 333 | 340 | 345 | 349 |
| 255 | 303 | 306 | 310 | 317 | 325 | 333 | 341 | 345 | 349 |
| 256 | 303 | 307 | 311 | 318 | 326 | 334 | 342 | 346 | 350 |
| 257 | 304 | 308 | 312 | 319 | 327 | 335 | 342 | 347 | 351 |
| 258 | 305 | 308 | 312 | 319 | 327 | 336 | 343 | 348 | 352 |
| 259 | 305 | 309 | 313 | 320 | 328 | 336 | 344 | 349 | 353 |
| 260 | 306 | 309 | 314 | 321 | 329 | 337 | 345 | 349 | 354 |
| 261 | 306 | 310 | 314 | 321 | 329 | 338 | 346 | 350 | 354 |
| 262 | 307 | 310 | 315 | 322 | 330 | 339 | 346 | 351 | 355 |
| 263 | 307 | 311 | 315 | 322 | 331 | 339 | 347 | 352 | 356 |
| 264 | 308 | 312 | 316 | 323 | 331 | 340 | 348 | 352 | 357 |
| 265 | 308 | 312 | 316 | 324 | 332 | 341 | 348 | 353 | 357 |
| 266 | 309 | 313 | 317 | 324 | 333 | 341 | 349 | 354 | 358 |
| 267 | 309 | 313 | 317 | 325 | 333 | 342 | 350 | 355 | 359 |
| 268 | 310 | 314 | 318 | 325 | 334 | 342 | 350 | 355 | 360 |
| 269 | 310 | 314 | 318 | 326 | 334 | 343 | 351 | 356 | 360 |
| 270 | 311 | 314 | 319 | 326 | 335 | 344 | 352 | 357 | 361 |
| 271 | 311 | 315 | 319 | 327 | 336 | 344 | 352 | 357 | 362 |
| 272 | 312 | 315 | 320 | 327 | 336 | 345 | 353 | 358 | 362 |
| 273 | 312 | 316 | 320 | 328 | 337 | 346 | 354 | 359 | 363 |
| 274 | 312 | 316 | 321 | 328 | 337 | 346 | 354 | 359 | 364 |
| 275 | 313 | 317 | 321 | 329 | 338 | 347 | 355 | 360 | 365 |
| 276 | 313 | 317 | 322 | 329 | 338 | 347 | 356 | 361 | 365 |
| 277 | 313 | 317 | 322 | 330 | 339 | 348 | 356 | 361 | 366 |
| 278 | 314 | 318 | 322 | 330 | 339 | 348 | 357 | 362 | 366 |
| 279 | 314 | 318 | 323 | 331 | 340 | 349 | 357 | 363 | 367 |
| 280 | 315 | 319 | 323 | 331 | 340 | 349 | 358 | 363 | 368 |
| 281 | 315 | 319 | 324 | 331 | 341 | 350 | 358 | 364 | 368 |
| 282 | 315 | 319 | 324 | 332 | 341 | 350 | 359 | 364 | 369 |
| 283 | 315 | 320 | 324 | 332 | 341 | 351 | 360 | 365 | 370 |
| 284 | 316 | 320 | 325 | 333 | 342 | 351 | 360 | 365 | 370 |
| 285 | 316 | 320 | 325 | 333 | 342 | 352 | 361 | 366 | 371 |
| 286 | 316 | 320 | 325 | 333 | 343 | 352 | 361 | 367 | 371 |
| 287 | 317 | 321 | 325 | 334 | 343 | 353 | 362 | 367 | 372 |
| 288 | 317 | 321 | 326 | 334 | 343 | 353 | 362 | 368 | 372 |
| 289 | 317 | 321 | 326 | 334 | 344 | 354 | 363 | 368 | 373 |
| 290 | 317 | 321 | 326 | 335 | 344 | 354 | 363 | 369 | 373 |
| 291 | 317 | 322 | 327 | 335 | 345 | 354 | 364 | 369 | 374 |
| 292 | 318 | 322 | 327 | 335 | 345 | 355 | 364 | 370 | 375 |
| 293 | 318 | 322 | 327 | 336 | 345 | 355 | 364 | 370 | 375 |
| 294 | 318 | 322 | 327 | 336 | 346 | 356 | 365 | 371 | 376 |

Mean and variance equation for HC in males and females:

*E(Z*_i_) = 8.475388695560605 + [-14.35535180588728 GA_i_^-0.5^] + [-0.0002352579455448 GA_i_^2^]

*Var(Z*_i_) = 0.0183844497195926 + [0.2541680653875657 GA_i_^-1^] + [-0.1331594083132348 GA_i_^-0.5]^ + [-8.24021018612e-06 GA_i_^2^] + [0.0000146556506322 GA_i_^-0.5^GA_i_^2^] + [1.43492118971e-09 GA_i_^4^]
