# Supplementary material for: Swedish intrauterine growth reference ranges of biometric measurements of fetal head, abdomen and femur
Source: Sci Rep. 2020 Dec 31;10:22441. doi: 10.1038/s41598-020-79797-8 (PMC7775468; doi:10.1038/s41598-020-79797-8)
Supplement: Supplementary file 9 — Supplementary Table 9. [file 41598_2020_79797_MOESM9_ESM.docx]

Supplementary Table 9a. Estimated abdominal circumference (AC) in mm by gestational age (GA) for males and females, Standard deviations (SD).

| GA (days) | -3 SD | -2 SD | -1 SD | Median | +1 SD | +2 SD | +3 SD |
| --- | --- | --- | --- | --- | --- | --- | --- |
| 84 | 48 | 51 | 54 | 57 | 60 | 64 | 67 |
| 85 | 50 | 53 | 56 | 59 | 62 | 65 | 69 |
| 86 | 52 | 54 | 57 | 60 | 64 | 67 | 71 |
| 87 | 53 | 56 | 59 | 62 | 65 | 69 | 73 |
| 88 | 55 | 58 | 61 | 64 | 67 | 71 | 74 |
| 89 | 56 | 59 | 62 | 65 | 69 | 72 | 76 |
| 90 | 58 | 61 | 64 | 67 | 70 | 74 | 78 |
| 91 | 59 | 62 | 65 | 69 | 72 | 76 | 80 |
| 92 | 61 | 64 | 67 | 70 | 74 | 78 | 81 |
| 93 | 62 | 66 | 69 | 72 | 76 | 79 | 83 |
| 94 | 64 | 67 | 70 | 74 | 77 | 81 | 85 |
| 95 | 66 | 69 | 72 | 76 | 79 | 83 | 87 |
| 96 | 67 | 70 | 74 | 77 | 81 | 85 | 89 |
| 97 | 69 | 72 | 75 | 79 | 83 | 87 | 91 |
| 98 | 70 | 74 | 77 | 81 | 84 | 88 | 93 |
| 99 | 72 | 75 | 79 | 82 | 86 | 90 | 94 |
| 100 | 73 | 77 | 80 | 84 | 88 | 92 | 96 |
| 101 | 75 | 78 | 82 | 86 | 90 | 94 | 98 |
| 102 | 77 | 80 | 84 | 88 | 92 | 96 | 100 |
| 103 | 78 | 82 | 85 | 89 | 93 | 98 | 102 |
| 104 | 80 | 83 | 87 | 91 | 95 | 100 | 104 |
| 105 | 81 | 85 | 89 | 93 | 97 | 101 | 106 |
| 106 | 83 | 87 | 90 | 95 | 99 | 103 | 108 |
| 107 | 84 | 88 | 92 | 96 | 101 | 105 | 110 |
| 108 | 86 | 90 | 94 | 98 | 102 | 107 | 112 |
| 109 | 87 | 91 | 95 | 100 | 104 | 109 | 114 |
| 110 | 89 | 93 | 97 | 101 | 106 | 111 | 116 |
| 111 | 91 | 95 | 99 | 103 | 108 | 113 | 118 |
| 112 | 92 | 96 | 100 | 105 | 110 | 115 | 120 |
| 113 | 94 | 98 | 102 | 107 | 112 | 116 | 122 |
| 114 | 95 | 99 | 104 | 108 | 113 | 118 | 124 |
| 115 | 97 | 101 | 106 | 110 | 115 | 120 | 126 |
| 116 | 98 | 103 | 107 | 112 | 117 | 122 | 128 |
| 117 | 100 | 104 | 109 | 114 | 119 | 124 | 130 |
| 118 | 101 | 106 | 111 | 115 | 121 | 126 | 132 |
| 119 | 103 | 107 | 112 | 117 | 122 | 128 | 134 |
| 120 | 104 | 109 | 114 | 119 | 124 | 130 | 136 |
| 121 | 106 | 111 | 116 | 121 | 126 | 132 | 138 |
| 122 | 108 | 112 | 117 | 123 | 128 | 134 | 140 |
| 123 | 109 | 114 | 119 | 124 | 130 | 136 | 142 |
| 124 | 111 | 116 | 121 | 126 | 132 | 137 | 144 |
| 125 | 112 | 117 | 122 | 128 | 133 | 139 | 146 |
| 126 | 114 | 119 | 124 | 129 | 135 | 141 | 147 |
| 127 | 115 | 120 | 126 | 131 | 137 | 143 | 149 |
| 128 | 117 | 122 | 127 | 133 | 139 | 145 | 151 |
| 129 | 118 | 124 | 129 | 135 | 141 | 147 | 153 |
| 130 | 120 | 125 | 131 | 136 | 143 | 149 | 155 |
| 131 | 121 | 127 | 132 | 138 | 144 | 151 | 157 |
| 132 | 123 | 128 | 134 | 140 | 146 | 153 | 159 |
| 133 | 124 | 130 | 136 | 142 | 148 | 155 | 161 |
| 134 | 126 | 132 | 137 | 143 | 150 | 156 | 163 |
| 135 | 127 | 133 | 139 | 145 | 152 | 158 | 165 |
| 136 | 129 | 135 | 141 | 147 | 153 | 160 | 167 |
| 137 | 130 | 136 | 142 | 149 | 155 | 162 | 169 |
| 138 | 132 | 138 | 144 | 150 | 157 | 164 | 171 |
| 139 | 134 | 139 | 146 | 152 | 159 | 166 | 173 |
| 140 | 135 | 141 | 147 | 154 | 161 | 168 | 175 |
| 141 | 137 | 143 | 149 | 156 | 162 | 170 | 177 |
| 142 | 138 | 144 | 151 | 157 | 164 | 172 | 179 |
| 143 | 140 | 146 | 152 | 159 | 166 | 173 | 181 |
| 144 | 141 | 147 | 154 | 161 | 168 | 175 | 183 |
| 145 | 143 | 149 | 156 | 162 | 170 | 177 | 185 |
| 146 | 144 | 150 | 157 | 164 | 171 | 179 | 187 |
| 147 | 146 | 152 | 159 | 166 | 173 | 181 | 189 |
| 148 | 147 | 154 | 160 | 168 | 175 | 183 | 191 |
| 149 | 149 | 155 | 162 | 169 | 177 | 185 | 193 |
| 150 | 150 | 157 | 164 | 171 | 179 | 187 | 195 |
| 151 | 151 | 158 | 165 | 173 | 180 | 188 | 197 |
| 152 | 153 | 160 | 167 | 174 | 182 | 190 | 199 |
| 153 | 154 | 161 | 169 | 176 | 184 | 192 | 201 |
| 154 | 156 | 163 | 170 | 178 | 186 | 194 | 203 |
| 155 | 157 | 164 | 172 | 179 | 187 | 196 | 205 |
| 156 | 159 | 166 | 173 | 181 | 189 | 198 | 207 |
| 157 | 160 | 167 | 175 | 183 | 191 | 200 | 208 |
| 158 | 162 | 169 | 177 | 184 | 193 | 201 | 210 |
| 159 | 163 | 171 | 178 | 186 | 195 | 203 | 212 |
| 160 | 165 | 172 | 180 | 188 | 196 | 205 | 214 |
| 161 | 166 | 174 | 181 | 190 | 198 | 207 | 216 |
| 162 | 168 | 175 | 183 | 191 | 200 | 209 | 218 |
| 163 | 169 | 177 | 185 | 193 | 202 | 211 | 220 |
| 164 | 170 | 178 | 186 | 195 | 203 | 212 | 222 |
| 165 | 172 | 180 | 188 | 196 | 205 | 214 | 224 |
| 166 | 173 | 181 | 189 | 198 | 207 | 216 | 226 |
| 167 | 175 | 183 | 191 | 199 | 208 | 218 | 228 |
| 168 | 176 | 184 | 192 | 201 | 210 | 220 | 230 |
| 169 | 178 | 186 | 194 | 203 | 212 | 222 | 232 |
| 170 | 179 | 187 | 196 | 204 | 214 | 223 | 233 |
| 171 | 180 | 189 | 197 | 206 | 215 | 225 | 235 |
| 172 | 182 | 190 | 199 | 208 | 217 | 227 | 237 |
| 173 | 183 | 192 | 200 | 209 | 219 | 229 | 239 |
| 174 | 185 | 193 | 202 | 211 | 221 | 231 | 241 |
| 175 | 186 | 195 | 203 | 213 | 222 | 232 | 243 |
| 176 | 187 | 196 | 205 | 214 | 224 | 234 | 245 |
| 177 | 189 | 197 | 206 | 216 | 226 | 236 | 247 |
| 178 | 190 | 199 | 208 | 217 | 227 | 238 | 249 |
| 179 | 192 | 200 | 210 | 219 | 229 | 240 | 251 |
| 180 | 193 | 202 | 211 | 221 | 231 | 241 | 252 |
| 181 | 194 | 203 | 213 | 222 | 233 | 243 | 254 |
| 182 | 196 | 205 | 214 | 224 | 234 | 245 | 256 |
| 183 | 197 | 206 | 216 | 226 | 236 | 247 | 258 |
| 184 | 199 | 208 | 217 | 227 | 238 | 249 | 260 |
| 185 | 200 | 209 | 219 | 229 | 239 | 250 | 262 |
| 186 | 201 | 210 | 220 | 230 | 241 | 252 | 264 |
| 187 | 203 | 212 | 222 | 232 | 243 | 254 | 266 |
| 188 | 204 | 213 | 223 | 234 | 244 | 256 | 267 |
| 189 | 205 | 215 | 225 | 235 | 246 | 257 | 269 |
| 190 | 207 | 216 | 226 | 237 | 248 | 259 | 271 |
| 191 | 208 | 218 | 228 | 238 | 249 | 261 | 273 |
| 192 | 209 | 219 | 229 | 240 | 251 | 263 | 275 |
| 193 | 211 | 220 | 231 | 241 | 253 | 264 | 277 |
| 194 | 212 | 222 | 232 | 243 | 254 | 266 | 279 |
| 195 | 213 | 223 | 234 | 245 | 256 | 268 | 280 |
| 196 | 215 | 225 | 235 | 246 | 258 | 270 | 282 |
| 197 | 216 | 226 | 237 | 248 | 259 | 271 | 284 |
| 198 | 217 | 227 | 238 | 249 | 261 | 273 | 286 |
| 199 | 219 | 229 | 240 | 251 | 263 | 275 | 288 |
| 200 | 220 | 230 | 241 | 252 | 264 | 277 | 290 |
| 201 | 221 | 232 | 242 | 254 | 266 | 278 | 292 |
| 202 | 222 | 233 | 244 | 255 | 267 | 280 | 293 |
| 203 | 224 | 234 | 245 | 257 | 269 | 282 | 295 |
| 204 | 225 | 236 | 247 | 259 | 271 | 284 | 297 |
| 205 | 226 | 237 | 248 | 260 | 272 | 285 | 299 |
| 206 | 228 | 238 | 250 | 262 | 274 | 287 | 301 |
| 207 | 229 | 240 | 251 | 263 | 276 | 289 | 303 |
| 208 | 230 | 241 | 253 | 265 | 277 | 290 | 304 |
| 209 | 231 | 242 | 254 | 266 | 279 | 292 | 306 |
| 210 | 233 | 244 | 255 | 268 | 280 | 294 | 308 |
| 211 | 234 | 245 | 257 | 269 | 282 | 296 | 310 |
| 212 | 235 | 246 | 258 | 271 | 284 | 297 | 312 |
| 213 | 236 | 248 | 260 | 272 | 285 | 299 | 313 |
| 214 | 238 | 249 | 261 | 274 | 287 | 301 | 315 |
| 215 | 239 | 250 | 263 | 275 | 288 | 302 | 317 |
| 216 | 240 | 252 | 264 | 277 | 290 | 304 | 319 |
| 217 | 241 | 253 | 265 | 278 | 292 | 306 | 321 |
| 218 | 243 | 254 | 267 | 280 | 293 | 308 | 322 |
| 219 | 244 | 256 | 268 | 281 | 295 | 309 | 324 |
| 220 | 245 | 257 | 269 | 283 | 296 | 311 | 326 |
| 221 | 246 | 258 | 271 | 284 | 298 | 313 | 328 |
| 222 | 247 | 260 | 272 | 286 | 300 | 314 | 330 |
| 223 | 249 | 261 | 274 | 287 | 301 | 316 | 331 |
| 224 | 250 | 262 | 275 | 289 | 303 | 318 | 333 |
| 225 | 251 | 263 | 276 | 290 | 304 | 319 | 335 |
| 226 | 252 | 265 | 278 | 291 | 306 | 321 | 337 |
| 227 | 253 | 266 | 279 | 293 | 307 | 323 | 339 |
| 228 | 255 | 267 | 280 | 294 | 309 | 324 | 340 |
| 229 | 256 | 269 | 282 | 296 | 311 | 326 | 342 |
| 230 | 257 | 270 | 283 | 297 | 312 | 328 | 344 |
| 231 | 258 | 271 | 285 | 299 | 314 | 329 | 346 |
| 232 | 259 | 272 | 286 | 300 | 315 | 331 | 348 |
| 233 | 260 | 274 | 287 | 302 | 317 | 333 | 349 |
| 234 | 262 | 275 | 289 | 303 | 318 | 334 | 351 |
| 235 | 263 | 276 | 290 | 305 | 320 | 336 | 353 |
| 236 | 264 | 277 | 291 | 306 | 321 | 338 | 355 |
| 237 | 265 | 279 | 293 | 307 | 323 | 339 | 356 |
| 238 | 266 | 280 | 294 | 309 | 324 | 341 | 358 |
| 239 | 267 | 281 | 295 | 310 | 326 | 343 | 360 |
| 240 | 269 | 282 | 297 | 312 | 328 | 344 | 362 |
| 241 | 270 | 283 | 298 | 313 | 329 | 346 | 363 |
| 242 | 271 | 285 | 299 | 314 | 331 | 347 | 365 |
| 243 | 272 | 286 | 301 | 316 | 332 | 349 | 367 |
| 244 | 273 | 287 | 302 | 317 | 334 | 351 | 369 |
| 245 | 274 | 288 | 303 | 319 | 335 | 352 | 370 |
| 246 | 275 | 290 | 304 | 320 | 337 | 354 | 372 |
| 247 | 276 | 291 | 306 | 322 | 338 | 356 | 374 |
| 248 | 278 | 292 | 307 | 323 | 340 | 357 | 376 |
| 249 | 279 | 293 | 308 | 324 | 341 | 359 | 377 |
| 250 | 280 | 294 | 310 | 326 | 343 | 360 | 379 |
| 251 | 281 | 295 | 311 | 327 | 344 | 362 | 381 |
| 252 | 282 | 297 | 312 | 328 | 346 | 364 | 383 |
| 253 | 283 | 298 | 313 | 330 | 347 | 365 | 384 |
| 254 | 284 | 299 | 315 | 331 | 349 | 367 | 386 |
| 255 | 285 | 300 | 316 | 333 | 350 | 369 | 388 |
| 256 | 286 | 301 | 317 | 334 | 352 | 370 | 390 |
| 257 | 287 | 303 | 319 | 335 | 353 | 372 | 391 |
| 258 | 288 | 304 | 320 | 337 | 355 | 373 | 393 |
| 259 | 290 | 305 | 321 | 338 | 356 | 375 | 395 |
| 260 | 291 | 306 | 322 | 339 | 357 | 377 | 397 |
| 261 | 292 | 307 | 324 | 341 | 359 | 378 | 398 |
| 262 | 293 | 308 | 325 | 342 | 360 | 380 | 400 |
| 263 | 294 | 309 | 326 | 344 | 362 | 381 | 402 |
| 264 | 295 | 311 | 327 | 345 | 363 | 383 | 403 |
| 265 | 296 | 312 | 329 | 346 | 365 | 384 | 405 |
| 266 | 297 | 313 | 330 | 348 | 366 | 386 | 407 |
| 267 | 298 | 314 | 331 | 349 | 368 | 388 | 409 |
| 268 | 299 | 315 | 332 | 350 | 369 | 389 | 410 |
| 269 | 300 | 316 | 333 | 352 | 371 | 391 | 412 |
| 270 | 301 | 317 | 335 | 353 | 372 | 392 | 414 |
| 271 | 302 | 319 | 336 | 354 | 374 | 394 | 415 |
| 272 | 303 | 320 | 337 | 356 | 375 | 395 | 417 |
| 273 | 304 | 321 | 338 | 357 | 376 | 397 | 419 |
| 274 | 305 | 322 | 340 | 358 | 378 | 399 | 420 |
| 275 | 306 | 323 | 341 | 360 | 379 | 400 | 422 |
| 276 | 307 | 324 | 342 | 361 | 381 | 402 | 424 |
| 277 | 308 | 325 | 343 | 362 | 382 | 403 | 426 |
| 278 | 309 | 326 | 344 | 363 | 384 | 405 | 427 |
| 279 | 310 | 327 | 346 | 365 | 385 | 406 | 429 |
| 280 | 311 | 329 | 347 | 366 | 386 | 408 | 431 |
| 281 | 312 | 330 | 348 | 367 | 388 | 409 | 432 |
| 282 | 313 | 331 | 349 | 369 | 389 | 411 | 434 |
| 283 | 314 | 332 | 350 | 370 | 391 | 413 | 436 |
| 284 | 315 | 333 | 352 | 371 | 392 | 414 | 437 |
| 285 | 316 | 334 | 353 | 373 | 393 | 416 | 439 |
| 286 | 317 | 335 | 354 | 374 | 395 | 417 | 441 |
| 287 | 318 | 336 | 355 | 375 | 396 | 419 | 442 |
| 288 | 319 | 337 | 356 | 376 | 398 | 420 | 444 |
| 289 | 320 | 338 | 357 | 378 | 399 | 422 | 446 |
| 290 | 321 | 339 | 359 | 379 | 400 | 423 | 447 |
| 291 | 322 | 340 | 360 | 380 | 402 | 425 | 449 |
| 292 | 323 | 341 | 361 | 381 | 403 | 426 | 451 |
| 293 | 324 | 342 | 362 | 383 | 405 | 428 | 452 |
| 294 | 325 | 343 | 363 | 384 | 406 | 429 | 454 |

Mean and variance equation for AC in males and females:

*E(Z*_i_) = 7.800537079069353 + [-50.05235065244585 GA_i_^-2^] + [-11.80463933785626 GA_i_^-0.5^]

*Var(Z*_i_) = 0.0359192944867489 + [470.5473665227667 GA_i_^-4^] + [6.860014631698228 GA_i_^-2^] + [-0.3916055110949738 GA_i_^-0.5^] + [-21.39824174335798 GA_i_^-2^GA_i_^-0.5^] + [1.147347158396176 GA_i_^-1^]

Supplementary Table 9b. Estimated abdominal circumference in mm by gestational age (GA) for males and females, percentiles.

| GA (days) | 2.5^th^ | 5^th^ | 10^th^ | 25^th^ | Median | 75^th^ | 90^th^ | 95^th^ | 97.5^th^ |
| --- | --- | --- | --- | --- | --- | --- | --- | --- | --- |
| 84 | 51 | 52 | 53 | 55 | 57 | 59 | 61 | 63 | 64 |
| 85 | 53 | 54 | 55 | 57 | 59 | 61 | 63 | 64 | 65 |
| 86 | 54 | 55 | 56 | 58 | 60 | 63 | 65 | 66 | 67 |
| 87 | 56 | 57 | 58 | 60 | 62 | 64 | 66 | 68 | 69 |
| 88 | 58 | 59 | 60 | 62 | 64 | 66 | 68 | 69 | 70 |
| 89 | 59 | 60 | 61 | 63 | 65 | 68 | 70 | 71 | 72 |
| 90 | 61 | 62 | 63 | 65 | 67 | 69 | 71 | 73 | 74 |
| 91 | 62 | 63 | 65 | 67 | 69 | 71 | 73 | 75 | 76 |
| 92 | 64 | 65 | 66 | 68 | 70 | 73 | 75 | 76 | 77 |
| 93 | 66 | 67 | 68 | 70 | 72 | 74 | 77 | 78 | 79 |
| 94 | 67 | 68 | 69 | 71 | 74 | 76 | 78 | 80 | 81 |
| 95 | 69 | 70 | 71 | 73 | 76 | 78 | 80 | 82 | 83 |
| 96 | 70 | 72 | 73 | 75 | 77 | 80 | 82 | 83 | 85 |
| 97 | 72 | 73 | 74 | 77 | 79 | 81 | 84 | 85 | 86 |
| 98 | 74 | 75 | 76 | 78 | 81 | 83 | 86 | 87 | 88 |
| 99 | 75 | 76 | 78 | 80 | 82 | 85 | 87 | 89 | 90 |
| 100 | 77 | 78 | 79 | 82 | 84 | 87 | 89 | 91 | 92 |
| 101 | 79 | 80 | 81 | 83 | 86 | 88 | 91 | 92 | 94 |
| 102 | 80 | 81 | 83 | 85 | 88 | 90 | 93 | 94 | 96 |
| 103 | 82 | 83 | 84 | 87 | 89 | 92 | 95 | 96 | 97 |
| 104 | 83 | 85 | 86 | 88 | 91 | 94 | 96 | 98 | 99 |
| 105 | 85 | 86 | 88 | 90 | 93 | 96 | 98 | 100 | 101 |
| 106 | 87 | 88 | 89 | 92 | 95 | 97 | 100 | 102 | 103 |
| 107 | 88 | 90 | 91 | 93 | 96 | 99 | 102 | 104 | 105 |
| 108 | 90 | 91 | 93 | 95 | 98 | 101 | 104 | 105 | 107 |
| 109 | 92 | 93 | 94 | 97 | 100 | 103 | 106 | 107 | 109 |
| 110 | 93 | 94 | 96 | 99 | 101 | 105 | 107 | 109 | 111 |
| 111 | 95 | 96 | 98 | 100 | 103 | 106 | 109 | 111 | 112 |
| 112 | 96 | 98 | 99 | 102 | 105 | 108 | 111 | 113 | 114 |
| 113 | 98 | 99 | 101 | 104 | 107 | 110 | 113 | 115 | 116 |
| 114 | 100 | 101 | 103 | 105 | 108 | 112 | 115 | 117 | 118 |
| 115 | 101 | 103 | 104 | 107 | 110 | 114 | 117 | 118 | 120 |
| 116 | 103 | 104 | 106 | 109 | 112 | 115 | 118 | 120 | 122 |
| 117 | 104 | 106 | 108 | 110 | 114 | 117 | 120 | 122 | 124 |
| 118 | 106 | 108 | 109 | 112 | 115 | 119 | 122 | 124 | 126 |
| 119 | 108 | 109 | 111 | 114 | 117 | 121 | 124 | 126 | 128 |
| 120 | 109 | 111 | 113 | 116 | 119 | 123 | 126 | 128 | 130 |
| 121 | 111 | 112 | 114 | 117 | 121 | 124 | 128 | 130 | 131 |
| 122 | 113 | 114 | 116 | 119 | 123 | 126 | 130 | 132 | 133 |
| 123 | 114 | 116 | 118 | 121 | 124 | 128 | 131 | 133 | 135 |
| 124 | 116 | 117 | 119 | 122 | 126 | 130 | 133 | 135 | 137 |
| 125 | 117 | 119 | 121 | 124 | 128 | 132 | 135 | 137 | 139 |
| 126 | 119 | 121 | 122 | 126 | 129 | 133 | 137 | 139 | 141 |
| 127 | 121 | 122 | 124 | 127 | 131 | 135 | 139 | 141 | 143 |
| 128 | 122 | 124 | 126 | 129 | 133 | 137 | 141 | 143 | 145 |
| 129 | 124 | 125 | 127 | 131 | 135 | 139 | 142 | 145 | 147 |
| 130 | 125 | 127 | 129 | 133 | 136 | 141 | 144 | 147 | 149 |
| 131 | 127 | 129 | 131 | 134 | 138 | 142 | 146 | 148 | 150 |
| 132 | 129 | 130 | 132 | 136 | 140 | 144 | 148 | 150 | 152 |
| 133 | 130 | 132 | 134 | 138 | 142 | 146 | 150 | 152 | 154 |
| 134 | 132 | 134 | 136 | 139 | 143 | 148 | 152 | 154 | 156 |
| 135 | 133 | 135 | 137 | 141 | 145 | 149 | 153 | 156 | 158 |
| 136 | 135 | 137 | 139 | 143 | 147 | 151 | 155 | 158 | 160 |
| 137 | 137 | 138 | 141 | 144 | 149 | 153 | 157 | 160 | 162 |
| 138 | 138 | 140 | 142 | 146 | 150 | 155 | 159 | 162 | 164 |
| 139 | 140 | 142 | 144 | 148 | 152 | 157 | 161 | 163 | 166 |
| 140 | 141 | 143 | 145 | 149 | 154 | 158 | 163 | 165 | 167 |
| 141 | 143 | 145 | 147 | 151 | 156 | 160 | 164 | 167 | 169 |
| 142 | 144 | 146 | 149 | 153 | 157 | 162 | 166 | 169 | 171 |
| 143 | 146 | 148 | 150 | 154 | 159 | 164 | 168 | 171 | 173 |
| 144 | 148 | 150 | 152 | 156 | 161 | 165 | 170 | 173 | 175 |
| 145 | 149 | 151 | 154 | 158 | 162 | 167 | 172 | 174 | 177 |
| 146 | 151 | 153 | 155 | 159 | 164 | 169 | 174 | 176 | 179 |
| 147 | 152 | 154 | 157 | 161 | 166 | 171 | 175 | 178 | 181 |
| 148 | 154 | 156 | 158 | 163 | 168 | 173 | 177 | 180 | 182 |
| 149 | 155 | 158 | 160 | 164 | 169 | 174 | 179 | 182 | 184 |
| 150 | 157 | 159 | 162 | 166 | 171 | 176 | 181 | 184 | 186 |
| 151 | 159 | 161 | 163 | 168 | 173 | 178 | 183 | 186 | 188 |
| 152 | 160 | 162 | 165 | 169 | 174 | 180 | 184 | 187 | 190 |
| 153 | 162 | 164 | 166 | 171 | 176 | 181 | 186 | 189 | 192 |
| 154 | 163 | 165 | 168 | 173 | 178 | 183 | 188 | 191 | 194 |
| 155 | 165 | 167 | 170 | 174 | 179 | 185 | 190 | 193 | 196 |
| 156 | 166 | 169 | 171 | 176 | 181 | 187 | 192 | 195 | 197 |
| 157 | 168 | 170 | 173 | 177 | 183 | 188 | 193 | 196 | 199 |
| 158 | 169 | 172 | 174 | 179 | 184 | 190 | 195 | 198 | 201 |
| 159 | 171 | 173 | 176 | 181 | 186 | 192 | 197 | 200 | 203 |
| 160 | 172 | 175 | 178 | 182 | 188 | 193 | 199 | 202 | 205 |
| 161 | 174 | 176 | 179 | 184 | 190 | 195 | 200 | 204 | 207 |
| 162 | 175 | 178 | 181 | 186 | 191 | 197 | 202 | 206 | 208 |
| 163 | 177 | 179 | 182 | 187 | 193 | 199 | 204 | 207 | 210 |
| 164 | 178 | 181 | 184 | 189 | 195 | 200 | 206 | 209 | 212 |
| 165 | 180 | 182 | 185 | 190 | 196 | 202 | 208 | 211 | 214 |
| 166 | 181 | 184 | 187 | 192 | 198 | 204 | 209 | 213 | 216 |
| 167 | 183 | 186 | 189 | 194 | 199 | 206 | 211 | 215 | 218 |
| 168 | 184 | 187 | 190 | 195 | 201 | 207 | 213 | 216 | 219 |
| 169 | 186 | 189 | 192 | 197 | 203 | 209 | 215 | 218 | 221 |
| 170 | 187 | 190 | 193 | 198 | 204 | 211 | 216 | 220 | 223 |
| 171 | 189 | 192 | 195 | 200 | 206 | 212 | 218 | 222 | 225 |
| 172 | 190 | 193 | 196 | 202 | 208 | 214 | 220 | 223 | 227 |
| 173 | 192 | 195 | 198 | 203 | 209 | 216 | 222 | 225 | 228 |
| 174 | 193 | 196 | 199 | 205 | 211 | 217 | 223 | 227 | 230 |
| 175 | 195 | 198 | 201 | 206 | 213 | 219 | 225 | 229 | 232 |
| 176 | 196 | 199 | 202 | 208 | 214 | 221 | 227 | 231 | 234 |
| 177 | 198 | 201 | 204 | 209 | 216 | 222 | 229 | 232 | 236 |
| 178 | 199 | 202 | 205 | 211 | 217 | 224 | 230 | 234 | 237 |
| 179 | 201 | 204 | 207 | 213 | 219 | 226 | 232 | 236 | 239 |
| 180 | 202 | 205 | 208 | 214 | 221 | 227 | 234 | 238 | 241 |
| 181 | 204 | 207 | 210 | 216 | 222 | 229 | 235 | 239 | 243 |
| 182 | 205 | 208 | 211 | 217 | 224 | 231 | 237 | 241 | 245 |
| 183 | 207 | 209 | 213 | 219 | 226 | 232 | 239 | 243 | 246 |
| 184 | 208 | 211 | 214 | 220 | 227 | 234 | 241 | 245 | 248 |
| 185 | 209 | 212 | 216 | 222 | 229 | 236 | 242 | 246 | 250 |
| 186 | 211 | 214 | 217 | 223 | 230 | 237 | 244 | 248 | 252 |
| 187 | 212 | 215 | 219 | 225 | 232 | 239 | 246 | 250 | 253 |
| 188 | 214 | 217 | 220 | 227 | 234 | 241 | 247 | 252 | 255 |
| 189 | 215 | 218 | 222 | 228 | 235 | 242 | 249 | 253 | 257 |
| 190 | 217 | 220 | 223 | 230 | 237 | 244 | 251 | 255 | 259 |
| 191 | 218 | 221 | 225 | 231 | 238 | 246 | 253 | 257 | 260 |
| 192 | 219 | 223 | 226 | 233 | 240 | 247 | 254 | 258 | 262 |
| 193 | 221 | 224 | 228 | 234 | 241 | 249 | 256 | 260 | 264 |
| 194 | 222 | 225 | 229 | 236 | 243 | 251 | 258 | 262 | 266 |
| 195 | 224 | 227 | 231 | 237 | 245 | 252 | 259 | 264 | 267 |
| 196 | 225 | 228 | 232 | 239 | 246 | 254 | 261 | 265 | 269 |
| 197 | 226 | 230 | 234 | 240 | 248 | 255 | 263 | 267 | 271 |
| 198 | 228 | 231 | 235 | 242 | 249 | 257 | 264 | 269 | 273 |
| 199 | 229 | 233 | 236 | 243 | 251 | 259 | 266 | 270 | 274 |
| 200 | 231 | 234 | 238 | 245 | 252 | 260 | 268 | 272 | 276 |
| 201 | 232 | 235 | 239 | 246 | 254 | 262 | 269 | 274 | 278 |
| 202 | 233 | 237 | 241 | 248 | 255 | 263 | 271 | 276 | 280 |
| 203 | 235 | 238 | 242 | 249 | 257 | 265 | 273 | 277 | 281 |
| 204 | 236 | 240 | 244 | 251 | 259 | 267 | 274 | 279 | 283 |
| 205 | 237 | 241 | 245 | 252 | 260 | 268 | 276 | 281 | 285 |
| 206 | 239 | 242 | 246 | 254 | 262 | 270 | 278 | 282 | 287 |
| 207 | 240 | 244 | 248 | 255 | 263 | 271 | 279 | 284 | 288 |
| 208 | 242 | 245 | 249 | 256 | 265 | 273 | 281 | 286 | 290 |
| 209 | 243 | 246 | 251 | 258 | 266 | 275 | 283 | 287 | 292 |
| 210 | 244 | 248 | 252 | 259 | 268 | 276 | 284 | 289 | 293 |
| 211 | 246 | 249 | 253 | 261 | 269 | 278 | 286 | 291 | 295 |
| 212 | 247 | 251 | 255 | 262 | 271 | 279 | 287 | 292 | 297 |
| 213 | 248 | 252 | 256 | 264 | 272 | 281 | 289 | 294 | 298 |
| 214 | 250 | 253 | 258 | 265 | 274 | 283 | 291 | 296 | 300 |
| 215 | 251 | 255 | 259 | 267 | 275 | 284 | 292 | 297 | 302 |
| 216 | 252 | 256 | 260 | 268 | 277 | 286 | 294 | 299 | 304 |
| 217 | 254 | 257 | 262 | 269 | 278 | 287 | 296 | 301 | 305 |
| 218 | 255 | 259 | 263 | 271 | 280 | 289 | 297 | 302 | 307 |
| 219 | 256 | 260 | 265 | 272 | 281 | 290 | 299 | 304 | 309 |
| 220 | 257 | 261 | 266 | 274 | 283 | 292 | 300 | 306 | 310 |
| 221 | 259 | 263 | 267 | 275 | 284 | 293 | 302 | 307 | 312 |
| 222 | 260 | 264 | 269 | 277 | 286 | 295 | 304 | 309 | 314 |
| 223 | 261 | 265 | 270 | 278 | 287 | 297 | 305 | 311 | 315 |
| 224 | 263 | 267 | 271 | 279 | 289 | 298 | 307 | 312 | 317 |
| 225 | 264 | 268 | 273 | 281 | 290 | 300 | 308 | 314 | 319 |
| 226 | 265 | 269 | 274 | 282 | 291 | 301 | 310 | 316 | 320 |
| 227 | 266 | 271 | 275 | 284 | 293 | 303 | 312 | 317 | 322 |
| 228 | 268 | 272 | 277 | 285 | 294 | 304 | 313 | 319 | 324 |
| 229 | 269 | 273 | 278 | 286 | 296 | 306 | 315 | 320 | 325 |
| 230 | 270 | 274 | 279 | 288 | 297 | 307 | 316 | 322 | 327 |
| 231 | 272 | 276 | 281 | 289 | 299 | 309 | 318 | 324 | 329 |
| 232 | 273 | 277 | 282 | 290 | 300 | 310 | 320 | 325 | 330 |
| 233 | 274 | 278 | 283 | 292 | 302 | 312 | 321 | 327 | 332 |
| 234 | 275 | 280 | 285 | 293 | 303 | 313 | 323 | 329 | 334 |
| 235 | 277 | 281 | 286 | 295 | 305 | 315 | 324 | 330 | 335 |
| 236 | 278 | 282 | 287 | 296 | 306 | 316 | 326 | 332 | 337 |
| 237 | 279 | 283 | 289 | 297 | 307 | 318 | 327 | 333 | 339 |
| 238 | 280 | 285 | 290 | 299 | 309 | 319 | 329 | 335 | 340 |
| 239 | 282 | 286 | 291 | 300 | 310 | 321 | 331 | 337 | 342 |
| 240 | 283 | 287 | 292 | 301 | 312 | 322 | 332 | 338 | 344 |
| 241 | 284 | 288 | 294 | 303 | 313 | 324 | 334 | 340 | 345 |
| 242 | 285 | 290 | 295 | 304 | 314 | 325 | 335 | 341 | 347 |
| 243 | 286 | 291 | 296 | 305 | 316 | 327 | 337 | 343 | 348 |
| 244 | 288 | 292 | 298 | 307 | 317 | 328 | 338 | 345 | 350 |
| 245 | 289 | 293 | 299 | 308 | 319 | 330 | 340 | 346 | 352 |
| 246 | 290 | 295 | 300 | 309 | 320 | 331 | 341 | 348 | 353 |
| 247 | 291 | 296 | 301 | 311 | 322 | 333 | 343 | 349 | 355 |
| 248 | 293 | 297 | 303 | 312 | 323 | 334 | 345 | 351 | 357 |
| 249 | 294 | 298 | 304 | 313 | 324 | 336 | 346 | 352 | 358 |
| 250 | 295 | 300 | 305 | 315 | 326 | 337 | 348 | 354 | 360 |
| 251 | 296 | 301 | 306 | 316 | 327 | 338 | 349 | 356 | 361 |
| 252 | 297 | 302 | 308 | 317 | 328 | 340 | 351 | 357 | 363 |
| 253 | 298 | 303 | 309 | 319 | 330 | 341 | 352 | 359 | 365 |
| 254 | 300 | 305 | 310 | 320 | 331 | 343 | 354 | 360 | 366 |
| 255 | 301 | 306 | 311 | 321 | 333 | 344 | 355 | 362 | 368 |
| 256 | 302 | 307 | 313 | 323 | 334 | 346 | 357 | 363 | 369 |
| 257 | 303 | 308 | 314 | 324 | 335 | 347 | 358 | 365 | 371 |
| 258 | 304 | 309 | 315 | 325 | 337 | 349 | 360 | 367 | 373 |
| 259 | 306 | 311 | 316 | 327 | 338 | 350 | 361 | 368 | 374 |
| 260 | 307 | 312 | 318 | 328 | 339 | 352 | 363 | 370 | 376 |
| 261 | 308 | 313 | 319 | 329 | 341 | 353 | 364 | 371 | 377 |
| 262 | 309 | 314 | 320 | 330 | 342 | 354 | 366 | 373 | 379 |
| 263 | 310 | 315 | 321 | 332 | 344 | 356 | 367 | 374 | 380 |
| 264 | 311 | 316 | 323 | 333 | 345 | 357 | 369 | 376 | 382 |
| 265 | 312 | 318 | 324 | 334 | 346 | 359 | 370 | 377 | 384 |
| 266 | 314 | 319 | 325 | 335 | 348 | 360 | 372 | 379 | 385 |
| 267 | 315 | 320 | 326 | 337 | 349 | 361 | 373 | 380 | 387 |
| 268 | 316 | 321 | 327 | 338 | 350 | 363 | 375 | 382 | 388 |
| 269 | 317 | 322 | 329 | 339 | 352 | 364 | 376 | 383 | 390 |
| 270 | 318 | 323 | 330 | 341 | 353 | 366 | 378 | 385 | 391 |
| 271 | 319 | 325 | 331 | 342 | 354 | 367 | 379 | 387 | 393 |
| 272 | 320 | 326 | 332 | 343 | 356 | 369 | 381 | 388 | 395 |
| 273 | 321 | 327 | 333 | 344 | 357 | 370 | 382 | 390 | 396 |
| 274 | 323 | 328 | 334 | 346 | 358 | 371 | 384 | 391 | 398 |
| 275 | 324 | 329 | 336 | 347 | 360 | 373 | 385 | 393 | 399 |
| 276 | 325 | 330 | 337 | 348 | 361 | 374 | 387 | 394 | 401 |
| 277 | 326 | 331 | 338 | 349 | 362 | 375 | 388 | 396 | 402 |
| 278 | 327 | 333 | 339 | 350 | 363 | 377 | 389 | 397 | 404 |
| 279 | 328 | 334 | 340 | 352 | 365 | 378 | 391 | 399 | 405 |
| 280 | 329 | 335 | 342 | 353 | 366 | 380 | 392 | 400 | 407 |
| 281 | 330 | 336 | 343 | 354 | 367 | 381 | 394 | 402 | 409 |
| 282 | 331 | 337 | 344 | 355 | 369 | 382 | 395 | 403 | 410 |
| 283 | 332 | 338 | 345 | 357 | 370 | 384 | 397 | 405 | 412 |
| 284 | 334 | 339 | 346 | 358 | 371 | 385 | 398 | 406 | 413 |
| 285 | 335 | 340 | 347 | 359 | 373 | 387 | 400 | 408 | 415 |
| 286 | 336 | 342 | 348 | 360 | 374 | 388 | 401 | 409 | 416 |
| 287 | 337 | 343 | 350 | 361 | 375 | 389 | 402 | 411 | 418 |
| 288 | 338 | 344 | 351 | 363 | 376 | 391 | 404 | 412 | 419 |
| 289 | 339 | 345 | 352 | 364 | 378 | 392 | 405 | 414 | 421 |
| 290 | 340 | 346 | 353 | 365 | 379 | 393 | 407 | 415 | 422 |
| 291 | 341 | 347 | 354 | 366 | 380 | 395 | 408 | 416 | 424 |
| 292 | 342 | 348 | 355 | 367 | 381 | 396 | 410 | 418 | 425 |
| 293 | 343 | 349 | 356 | 369 | 383 | 397 | 411 | 419 | 427 |
| 294 | 344 | 350 | 358 | 370 | 384 | 399 | 412 | 421 | 428 |

Mean and variance equation for AC in males and females:

*E(Z*_i_) = 7.800537079069353 + [-50.05235065244585 GA_i_^-2^] + [-11.80463933785626 GA_i_^-0.5^]

*Var(Z*_i_) = 0.0359192944867489 + [470.5473665227667 GA_i_^-4^] + [6.860014631698228 GA_i_^-2^] + [-0.3916055110949738 GA_i_^-0.5^] + [-21.39824174335798 GA_i_^-2^GA_i_^-0.5^] + [1.147347158396176 GA_i_^-1^]
