# Supplementary material for: Swedish intrauterine growth reference ranges of biometric measurements of fetal head, abdomen and femur
Source: Sci Rep. 2020 Dec 31;10:22441. doi: 10.1038/s41598-020-79797-8 (PMC7775468; doi:10.1038/s41598-020-79797-8)
Supplement: Supplementary file 10 — Supplementary Table 10. [file 41598_2020_79797_MOESM10_ESM.docx]

Supplementary Table 10a. Estimated femur length (FL) in mm by gestational age (GA) for males and females, Standard deviations (SD).

| GA (days) | -3 SD | -2 SD | -1 SD | Median | +1 SD | +2 SD | +3 SD |
| --- | --- | --- | --- | --- | --- | --- | --- |
| 84 | 5 | 5 | 6 | 6 | 7 | 8 | 9 |
| 85 | 5 | 5 | 6 | 7 | 7 | 8 | 9 |
| 86 | 5 | 6 | 6 | 7 | 8 | 9 | 10 |
| 87 | 6 | 6 | 7 | 7 | 8 | 9 | 10 |
| 88 | 6 | 6 | 7 | 8 | 9 | 10 | 10 |
| 89 | 6 | 7 | 7 | 8 | 9 | 10 | 11 |
| 90 | 7 | 7 | 8 | 9 | 9 | 10 | 11 |
| 91 | 7 | 8 | 8 | 9 | 10 | 11 | 12 |
| 92 | 7 | 8 | 9 | 9 | 10 | 11 | 12 |
| 93 | 8 | 8 | 9 | 10 | 11 | 12 | 13 |
| 94 | 8 | 9 | 10 | 10 | 11 | 12 | 13 |
| 95 | 8 | 9 | 10 | 11 | 12 | 13 | 14 |
| 96 | 9 | 10 | 10 | 11 | 12 | 13 | 14 |
| 97 | 9 | 10 | 11 | 12 | 13 | 14 | 15 |
| 98 | 10 | 10 | 11 | 12 | 13 | 14 | 15 |
| 99 | 10 | 11 | 12 | 13 | 14 | 15 | 16 |
| 100 | 11 | 11 | 12 | 13 | 14 | 15 | 16 |
| 101 | 11 | 12 | 13 | 13 | 14 | 15 | 17 |
| 102 | 11 | 12 | 13 | 14 | 15 | 16 | 17 |
| 103 | 12 | 13 | 13 | 14 | 15 | 16 | 18 |
| 104 | 12 | 13 | 14 | 15 | 16 | 17 | 18 |
| 105 | 13 | 14 | 14 | 15 | 16 | 17 | 19 |
| 106 | 13 | 14 | 15 | 16 | 17 | 18 | 19 |
| 107 | 14 | 14 | 15 | 16 | 17 | 18 | 20 |
| 108 | 14 | 15 | 16 | 17 | 18 | 19 | 20 |
| 109 | 14 | 15 | 16 | 17 | 18 | 19 | 21 |
| 110 | 15 | 16 | 17 | 18 | 19 | 20 | 21 |
| 111 | 15 | 16 | 17 | 18 | 19 | 20 | 22 |
| 112 | 16 | 17 | 18 | 19 | 20 | 21 | 22 |
| 113 | 16 | 17 | 18 | 19 | 20 | 21 | 23 |
| 114 | 17 | 18 | 19 | 20 | 21 | 22 | 23 |
| 115 | 17 | 18 | 19 | 20 | 21 | 22 | 24 |
| 116 | 18 | 19 | 20 | 21 | 22 | 23 | 24 |
| 117 | 18 | 19 | 20 | 21 | 22 | 23 | 25 |
| 118 | 18 | 19 | 20 | 22 | 23 | 24 | 25 |
| 119 | 19 | 20 | 21 | 22 | 23 | 24 | 26 |
| 120 | 19 | 20 | 21 | 22 | 24 | 25 | 26 |
| 121 | 20 | 21 | 22 | 23 | 24 | 25 | 27 |
| 122 | 20 | 21 | 22 | 23 | 25 | 26 | 27 |
| 123 | 21 | 22 | 23 | 24 | 25 | 26 | 28 |
| 124 | 21 | 22 | 23 | 24 | 26 | 27 | 28 |
| 125 | 22 | 23 | 24 | 25 | 26 | 27 | 29 |
| 126 | 22 | 23 | 24 | 25 | 27 | 28 | 29 |
| 127 | 22 | 23 | 25 | 26 | 27 | 28 | 30 |
| 128 | 23 | 24 | 25 | 26 | 27 | 29 | 30 |
| 129 | 23 | 24 | 25 | 27 | 28 | 29 | 31 |
| 130 | 24 | 25 | 26 | 27 | 28 | 30 | 31 |
| 131 | 24 | 25 | 26 | 28 | 29 | 30 | 32 |
| 132 | 24 | 26 | 27 | 28 | 29 | 31 | 32 |
| 133 | 25 | 26 | 27 | 28 | 30 | 31 | 33 |
| 134 | 25 | 26 | 28 | 29 | 30 | 32 | 33 |
| 135 | 26 | 27 | 28 | 29 | 31 | 32 | 34 |
| 136 | 26 | 27 | 29 | 30 | 31 | 33 | 34 |
| 137 | 27 | 28 | 29 | 30 | 32 | 33 | 35 |
| 138 | 27 | 28 | 29 | 31 | 32 | 34 | 35 |
| 139 | 27 | 29 | 30 | 31 | 33 | 34 | 36 |
| 140 | 28 | 29 | 30 | 32 | 33 | 34 | 36 |
| 141 | 28 | 29 | 31 | 32 | 33 | 35 | 36 |
| 142 | 29 | 30 | 31 | 32 | 34 | 35 | 37 |
| 143 | 29 | 30 | 32 | 33 | 34 | 36 | 37 |
| 144 | 29 | 31 | 32 | 33 | 35 | 36 | 38 |
| 145 | 30 | 31 | 32 | 34 | 35 | 37 | 38 |
| 146 | 30 | 31 | 33 | 34 | 36 | 37 | 39 |
| 147 | 30 | 32 | 33 | 35 | 36 | 38 | 39 |
| 148 | 31 | 32 | 34 | 35 | 37 | 38 | 40 |
| 149 | 31 | 33 | 34 | 35 | 37 | 39 | 40 |
| 150 | 32 | 33 | 34 | 36 | 37 | 39 | 41 |
| 151 | 32 | 33 | 35 | 36 | 38 | 39 | 41 |
| 152 | 32 | 34 | 35 | 37 | 38 | 40 | 42 |
| 153 | 33 | 34 | 36 | 37 | 39 | 40 | 42 |
| 154 | 33 | 34 | 36 | 37 | 39 | 41 | 43 |
| 155 | 33 | 35 | 36 | 38 | 39 | 41 | 43 |
| 156 | 34 | 35 | 37 | 38 | 40 | 42 | 43 |
| 157 | 34 | 36 | 37 | 39 | 40 | 42 | 44 |
| 158 | 34 | 36 | 37 | 39 | 41 | 42 | 44 |
| 159 | 35 | 36 | 38 | 39 | 41 | 43 | 45 |
| 160 | 35 | 37 | 38 | 40 | 42 | 43 | 45 |
| 161 | 35 | 37 | 39 | 40 | 42 | 44 | 46 |
| 162 | 36 | 37 | 39 | 41 | 42 | 44 | 46 |
| 163 | 36 | 38 | 39 | 41 | 43 | 45 | 46 |
| 164 | 36 | 38 | 40 | 41 | 43 | 45 | 47 |
| 165 | 37 | 38 | 40 | 42 | 44 | 45 | 47 |
| 166 | 37 | 39 | 40 | 42 | 44 | 46 | 48 |
| 167 | 37 | 39 | 41 | 42 | 44 | 46 | 48 |
| 168 | 38 | 39 | 41 | 43 | 45 | 47 | 49 |
| 169 | 38 | 40 | 41 | 43 | 45 | 47 | 49 |
| 170 | 38 | 40 | 42 | 44 | 45 | 47 | 49 |
| 171 | 39 | 40 | 42 | 44 | 46 | 48 | 50 |
| 172 | 39 | 41 | 42 | 44 | 46 | 48 | 50 |
| 173 | 39 | 41 | 43 | 45 | 47 | 49 | 51 |
| 174 | 40 | 41 | 43 | 45 | 47 | 49 | 51 |
| 175 | 40 | 42 | 44 | 45 | 47 | 49 | 51 |
| 176 | 40 | 42 | 44 | 46 | 48 | 50 | 52 |
| 177 | 41 | 42 | 44 | 46 | 48 | 50 | 52 |
| 178 | 41 | 43 | 45 | 46 | 48 | 50 | 53 |
| 179 | 41 | 43 | 45 | 47 | 49 | 51 | 53 |
| 180 | 42 | 43 | 45 | 47 | 49 | 51 | 53 |
| 181 | 42 | 44 | 46 | 47 | 49 | 52 | 54 |
| 182 | 42 | 44 | 46 | 48 | 50 | 52 | 54 |
| 183 | 42 | 44 | 46 | 48 | 50 | 52 | 55 |
| 184 | 43 | 45 | 46 | 48 | 51 | 53 | 55 |
| 185 | 43 | 45 | 47 | 49 | 51 | 53 | 55 |
| 186 | 43 | 45 | 47 | 49 | 51 | 53 | 56 |
| 187 | 44 | 45 | 47 | 49 | 52 | 54 | 56 |
| 188 | 44 | 46 | 48 | 50 | 52 | 54 | 56 |
| 189 | 44 | 46 | 48 | 50 | 52 | 54 | 57 |
| 190 | 44 | 46 | 48 | 50 | 53 | 55 | 57 |
| 191 | 45 | 47 | 49 | 51 | 53 | 55 | 58 |
| 192 | 45 | 47 | 49 | 51 | 53 | 56 | 58 |
| 193 | 45 | 47 | 49 | 51 | 54 | 56 | 58 |
| 194 | 46 | 48 | 50 | 52 | 54 | 56 | 59 |
| 195 | 46 | 48 | 50 | 52 | 54 | 57 | 59 |
| 196 | 46 | 48 | 50 | 52 | 55 | 57 | 59 |
| 197 | 46 | 48 | 50 | 53 | 55 | 57 | 60 |
| 198 | 47 | 49 | 51 | 53 | 55 | 58 | 60 |
| 199 | 47 | 49 | 51 | 53 | 56 | 58 | 60 |
| 200 | 47 | 49 | 51 | 54 | 56 | 58 | 61 |
| 201 | 48 | 50 | 52 | 54 | 56 | 59 | 61 |
| 202 | 48 | 50 | 52 | 54 | 56 | 59 | 61 |
| 203 | 48 | 50 | 52 | 54 | 57 | 59 | 62 |
| 204 | 48 | 50 | 53 | 55 | 57 | 60 | 62 |
| 205 | 49 | 51 | 53 | 55 | 57 | 60 | 62 |
| 206 | 49 | 51 | 53 | 55 | 58 | 60 | 63 |
| 207 | 49 | 51 | 53 | 56 | 58 | 61 | 63 |
| 208 | 49 | 51 | 54 | 56 | 58 | 61 | 63 |
| 209 | 50 | 52 | 54 | 56 | 59 | 61 | 64 |
| 210 | 50 | 52 | 54 | 57 | 59 | 61 | 64 |
| 211 | 50 | 52 | 55 | 57 | 59 | 62 | 64 |
| 212 | 50 | 53 | 55 | 57 | 60 | 62 | 65 |
| 213 | 51 | 53 | 55 | 57 | 60 | 62 | 65 |
| 214 | 51 | 53 | 55 | 58 | 60 | 63 | 65 |
| 215 | 51 | 53 | 56 | 58 | 60 | 63 | 66 |
| 216 | 51 | 54 | 56 | 58 | 61 | 63 | 66 |
| 217 | 52 | 54 | 56 | 59 | 61 | 64 | 66 |
| 218 | 52 | 54 | 56 | 59 | 61 | 64 | 67 |
| 219 | 52 | 54 | 57 | 59 | 62 | 64 | 67 |
| 220 | 52 | 55 | 57 | 59 | 62 | 65 | 67 |
| 221 | 53 | 55 | 57 | 60 | 62 | 65 | 68 |
| 222 | 53 | 55 | 57 | 60 | 62 | 65 | 68 |
| 223 | 53 | 55 | 58 | 60 | 63 | 65 | 68 |
| 224 | 53 | 56 | 58 | 60 | 63 | 66 | 69 |
| 225 | 54 | 56 | 58 | 61 | 63 | 66 | 69 |
| 226 | 54 | 56 | 59 | 61 | 64 | 66 | 69 |
| 227 | 54 | 56 | 59 | 61 | 64 | 67 | 69 |
| 228 | 54 | 57 | 59 | 62 | 64 | 67 | 70 |
| 229 | 55 | 57 | 59 | 62 | 64 | 67 | 70 |
| 230 | 55 | 57 | 60 | 62 | 65 | 67 | 70 |
| 231 | 55 | 57 | 60 | 62 | 65 | 68 | 71 |
| 232 | 55 | 58 | 60 | 63 | 65 | 68 | 71 |
| 233 | 55 | 58 | 60 | 63 | 66 | 68 | 71 |
| 234 | 56 | 58 | 61 | 63 | 66 | 69 | 72 |
| 235 | 56 | 58 | 61 | 63 | 66 | 69 | 72 |
| 236 | 56 | 59 | 61 | 64 | 66 | 69 | 72 |
| 237 | 56 | 59 | 61 | 64 | 67 | 69 | 72 |
| 238 | 57 | 59 | 62 | 64 | 67 | 70 | 73 |
| 239 | 57 | 59 | 62 | 64 | 67 | 70 | 73 |
| 240 | 57 | 59 | 62 | 65 | 67 | 70 | 73 |
| 241 | 57 | 60 | 62 | 65 | 68 | 71 | 74 |
| 242 | 57 | 60 | 63 | 65 | 68 | 71 | 74 |
| 243 | 58 | 60 | 63 | 65 | 68 | 71 | 74 |
| 244 | 58 | 60 | 63 | 66 | 68 | 71 | 74 |
| 245 | 58 | 61 | 63 | 66 | 69 | 72 | 75 |
| 246 | 58 | 61 | 63 | 66 | 69 | 72 | 75 |
| 247 | 59 | 61 | 64 | 66 | 69 | 72 | 75 |
| 248 | 59 | 61 | 64 | 67 | 70 | 72 | 76 |
| 249 | 59 | 62 | 64 | 67 | 70 | 73 | 76 |
| 250 | 59 | 62 | 64 | 67 | 70 | 73 | 76 |
| 251 | 59 | 62 | 65 | 67 | 70 | 73 | 76 |
| 252 | 60 | 62 | 65 | 68 | 71 | 74 | 77 |
| 253 | 60 | 62 | 65 | 68 | 71 | 74 | 77 |
| 254 | 60 | 63 | 65 | 68 | 71 | 74 | 77 |
| 255 | 60 | 63 | 66 | 68 | 71 | 74 | 78 |
| 256 | 60 | 63 | 66 | 69 | 72 | 75 | 78 |
| 257 | 61 | 63 | 66 | 69 | 72 | 75 | 78 |
| 258 | 61 | 64 | 66 | 69 | 72 | 75 | 78 |
| 259 | 61 | 64 | 66 | 69 | 72 | 75 | 79 |
| 260 | 61 | 64 | 67 | 70 | 73 | 76 | 79 |
| 261 | 62 | 64 | 67 | 70 | 73 | 76 | 79 |
| 262 | 62 | 64 | 67 | 70 | 73 | 76 | 79 |
| 263 | 62 | 65 | 67 | 70 | 73 | 76 | 80 |
| 264 | 62 | 65 | 68 | 71 | 74 | 77 | 80 |
| 265 | 62 | 65 | 68 | 71 | 74 | 77 | 80 |
| 266 | 63 | 65 | 68 | 71 | 74 | 77 | 81 |
| 267 | 63 | 65 | 68 | 71 | 74 | 77 | 81 |
| 268 | 63 | 66 | 68 | 71 | 75 | 78 | 81 |
| 269 | 63 | 66 | 69 | 72 | 75 | 78 | 81 |
| 270 | 63 | 66 | 69 | 72 | 75 | 78 | 82 |
| 271 | 64 | 66 | 69 | 72 | 75 | 79 | 82 |
| 272 | 64 | 66 | 69 | 72 | 75 | 79 | 82 |
| 273 | 64 | 67 | 70 | 73 | 76 | 79 | 82 |
| 274 | 64 | 67 | 70 | 73 | 76 | 79 | 83 |
| 275 | 64 | 67 | 70 | 73 | 76 | 80 | 83 |
| 276 | 64 | 67 | 70 | 73 | 76 | 80 | 83 |
| 277 | 65 | 67 | 70 | 73 | 77 | 80 | 83 |
| 278 | 65 | 68 | 71 | 74 | 77 | 80 | 84 |
| 279 | 65 | 68 | 71 | 74 | 77 | 81 | 84 |
| 280 | 65 | 68 | 71 | 74 | 77 | 81 | 84 |
| 281 | 65 | 68 | 71 | 74 | 78 | 81 | 85 |
| 282 | 66 | 68 | 71 | 75 | 78 | 81 | 85 |
| 283 | 66 | 69 | 72 | 75 | 78 | 82 | 85 |
| 284 | 66 | 69 | 72 | 75 | 78 | 82 | 85 |
| 285 | 66 | 69 | 72 | 75 | 79 | 82 | 86 |
| 286 | 66 | 69 | 72 | 76 | 79 | 82 | 86 |
| 287 | 67 | 69 | 73 | 76 | 79 | 83 | 86 |
| 288 | 67 | 70 | 73 | 76 | 79 | 83 | 86 |
| 289 | 67 | 70 | 73 | 76 | 80 | 83 | 87 |
| 290 | 67 | 70 | 73 | 76 | 80 | 83 | 87 |
| 291 | 67 | 70 | 73 | 77 | 80 | 84 | 87 |
| 292 | 67 | 70 | 74 | 77 | 80 | 84 | 87 |
| 293 | 68 | 71 | 74 | 77 | 80 | 84 | 88 |
| 294 | 68 | 71 | 74 | 77 | 81 | 84 | 88 |

Mean and variance equation for FL in males and females:

*E(Z*_i_) = 4.105732209910064 + [-344.2425559342237 GA_i_^-2^] + [0.0103936855030136 GA_i_^1^]

*Var(Z*_i_) = 0.0107011124191591 + [584.7103509618497 GA_i_^-4^] + [-4.456046516168862 GA_i_^-2^] + [-0.0004141491119216 GA_i_^1^] + [0.0502118462585746 GA_i_^-2^GA_i_^1^] + [4.83269392995e-06 GA_i_^2^]

Supplementary Table 10b. Estimated femur length (FL) in mm by gestational age (GA) for males and females, percentiles.

| GA (days) | 2.5^th^ | 5^th^ | 10^th^ | 25^th^ | Median | 75^th^ | 90^th^ | 95^th^ | 97.5^th^ |
| --- | --- | --- | --- | --- | --- | --- | --- | --- | --- |
| 84 | 5 | 5 | 5 | 6 | 6 | 7 | 7 | 8 | 8 |
| 85 | 5 | 6 | 6 | 6 | 7 | 7 | 8 | 8 | 8 |
| 86 | 6 | 6 | 6 | 7 | 7 | 8 | 8 | 8 | 9 |
| 87 | 6 | 6 | 7 | 7 | 7 | 8 | 8 | 9 | 9 |
| 88 | 6 | 7 | 7 | 7 | 8 | 8 | 9 | 9 | 9 |
| 89 | 7 | 7 | 7 | 8 | 8 | 9 | 9 | 10 | 10 |
| 90 | 7 | 7 | 8 | 8 | 9 | 9 | 10 | 10 | 10 |
| 91 | 8 | 8 | 8 | 9 | 9 | 10 | 10 | 10 | 11 |
| 92 | 8 | 8 | 8 | 9 | 9 | 10 | 11 | 11 | 11 |
| 93 | 8 | 9 | 9 | 9 | 10 | 10 | 11 | 11 | 12 |
| 94 | 9 | 9 | 9 | 10 | 10 | 11 | 11 | 12 | 12 |
| 95 | 9 | 9 | 10 | 10 | 11 | 11 | 12 | 12 | 13 |
| 96 | 10 | 10 | 10 | 11 | 11 | 12 | 12 | 13 | 13 |
| 97 | 10 | 10 | 11 | 11 | 12 | 12 | 13 | 13 | 14 |
| 98 | 10 | 11 | 11 | 12 | 12 | 13 | 13 | 14 | 14 |
| 99 | 11 | 11 | 11 | 12 | 13 | 13 | 14 | 14 | 14 |
| 100 | 11 | 12 | 12 | 12 | 13 | 14 | 14 | 15 | 15 |
| 101 | 12 | 12 | 12 | 13 | 13 | 14 | 15 | 15 | 15 |
| 102 | 12 | 12 | 13 | 13 | 14 | 15 | 15 | 16 | 16 |
| 103 | 13 | 13 | 13 | 14 | 14 | 15 | 16 | 16 | 16 |
| 104 | 13 | 13 | 14 | 14 | 15 | 16 | 16 | 17 | 17 |
| 105 | 14 | 14 | 14 | 15 | 15 | 16 | 17 | 17 | 17 |
| 106 | 14 | 14 | 15 | 15 | 16 | 17 | 17 | 18 | 18 |
| 107 | 14 | 15 | 15 | 16 | 16 | 17 | 18 | 18 | 18 |
| 108 | 15 | 15 | 16 | 16 | 17 | 17 | 18 | 19 | 19 |
| 109 | 15 | 16 | 16 | 17 | 17 | 18 | 19 | 19 | 19 |
| 110 | 16 | 16 | 16 | 17 | 18 | 18 | 19 | 19 | 20 |
| 111 | 16 | 17 | 17 | 18 | 18 | 19 | 20 | 20 | 20 |
| 112 | 17 | 17 | 17 | 18 | 19 | 19 | 20 | 20 | 21 |
| 113 | 17 | 18 | 18 | 18 | 19 | 20 | 21 | 21 | 21 |
| 114 | 18 | 18 | 18 | 19 | 20 | 20 | 21 | 21 | 22 |
| 115 | 18 | 18 | 19 | 19 | 20 | 21 | 22 | 22 | 22 |
| 116 | 19 | 19 | 19 | 20 | 21 | 21 | 22 | 22 | 23 |
| 117 | 19 | 19 | 20 | 20 | 21 | 22 | 23 | 23 | 23 |
| 118 | 19 | 20 | 20 | 21 | 22 | 22 | 23 | 23 | 24 |
| 119 | 20 | 20 | 21 | 21 | 22 | 23 | 23 | 24 | 24 |
| 120 | 20 | 21 | 21 | 22 | 22 | 23 | 24 | 24 | 25 |
| 121 | 21 | 21 | 22 | 22 | 23 | 24 | 24 | 25 | 25 |
| 122 | 21 | 22 | 22 | 23 | 23 | 24 | 25 | 25 | 26 |
| 123 | 22 | 22 | 22 | 23 | 24 | 25 | 25 | 26 | 26 |
| 124 | 22 | 23 | 23 | 24 | 24 | 25 | 26 | 26 | 27 |
| 125 | 23 | 23 | 23 | 24 | 25 | 26 | 26 | 27 | 27 |
| 126 | 23 | 23 | 24 | 24 | 25 | 26 | 27 | 27 | 28 |
| 127 | 24 | 24 | 24 | 25 | 26 | 27 | 27 | 28 | 28 |
| 128 | 24 | 24 | 25 | 25 | 26 | 27 | 28 | 28 | 29 |
| 129 | 24 | 25 | 25 | 26 | 27 | 28 | 28 | 29 | 29 |
| 130 | 25 | 25 | 26 | 26 | 27 | 28 | 29 | 29 | 30 |
| 131 | 25 | 26 | 26 | 27 | 28 | 28 | 29 | 30 | 30 |
| 132 | 26 | 26 | 26 | 27 | 28 | 29 | 30 | 30 | 31 |
| 133 | 26 | 26 | 27 | 28 | 28 | 29 | 30 | 31 | 31 |
| 134 | 27 | 27 | 27 | 28 | 29 | 30 | 31 | 31 | 32 |
| 135 | 27 | 27 | 28 | 29 | 29 | 30 | 31 | 32 | 32 |
| 136 | 27 | 28 | 28 | 29 | 30 | 31 | 32 | 32 | 33 |
| 137 | 28 | 28 | 29 | 29 | 30 | 31 | 32 | 33 | 33 |
| 138 | 28 | 29 | 29 | 30 | 31 | 32 | 32 | 33 | 33 |
| 139 | 29 | 29 | 29 | 30 | 31 | 32 | 33 | 33 | 34 |
| 140 | 29 | 29 | 30 | 31 | 32 | 33 | 33 | 34 | 34 |
| 141 | 29 | 30 | 30 | 31 | 32 | 33 | 34 | 34 | 35 |
| 142 | 30 | 30 | 31 | 32 | 32 | 33 | 34 | 35 | 35 |
| 143 | 30 | 31 | 31 | 32 | 33 | 34 | 35 | 35 | 36 |
| 144 | 31 | 31 | 32 | 32 | 33 | 34 | 35 | 36 | 36 |
| 145 | 31 | 31 | 32 | 33 | 34 | 35 | 36 | 36 | 37 |
| 146 | 31 | 32 | 32 | 33 | 34 | 35 | 36 | 37 | 37 |
| 147 | 32 | 32 | 33 | 34 | 35 | 36 | 37 | 37 | 38 |
| 148 | 32 | 33 | 33 | 34 | 35 | 36 | 37 | 38 | 38 |
| 149 | 33 | 33 | 34 | 34 | 35 | 36 | 37 | 38 | 38 |
| 150 | 33 | 33 | 34 | 35 | 36 | 37 | 38 | 38 | 39 |
| 151 | 33 | 34 | 34 | 35 | 36 | 37 | 38 | 39 | 39 |
| 152 | 34 | 34 | 35 | 36 | 37 | 38 | 39 | 39 | 40 |
| 153 | 34 | 35 | 35 | 36 | 37 | 38 | 39 | 40 | 40 |
| 154 | 34 | 35 | 35 | 36 | 37 | 39 | 40 | 40 | 41 |
| 155 | 35 | 35 | 36 | 37 | 38 | 39 | 40 | 41 | 41 |
| 156 | 35 | 36 | 36 | 37 | 38 | 39 | 40 | 41 | 42 |
| 157 | 36 | 36 | 37 | 38 | 39 | 40 | 41 | 41 | 42 |
| 158 | 36 | 36 | 37 | 38 | 39 | 40 | 41 | 42 | 42 |
| 159 | 36 | 37 | 37 | 38 | 39 | 41 | 42 | 42 | 43 |
| 160 | 37 | 37 | 38 | 39 | 40 | 41 | 42 | 43 | 43 |
| 161 | 37 | 38 | 38 | 39 | 40 | 41 | 42 | 43 | 44 |
| 162 | 37 | 38 | 38 | 39 | 41 | 42 | 43 | 43 | 44 |
| 163 | 38 | 38 | 39 | 40 | 41 | 42 | 43 | 44 | 44 |
| 164 | 38 | 39 | 39 | 40 | 41 | 43 | 44 | 44 | 45 |
| 165 | 38 | 39 | 40 | 41 | 42 | 43 | 44 | 45 | 45 |
| 166 | 39 | 39 | 40 | 41 | 42 | 43 | 44 | 45 | 46 |
| 167 | 39 | 40 | 40 | 41 | 42 | 44 | 45 | 46 | 46 |
| 168 | 39 | 40 | 41 | 42 | 43 | 44 | 45 | 46 | 47 |
| 169 | 40 | 40 | 41 | 42 | 43 | 44 | 46 | 46 | 47 |
| 170 | 40 | 41 | 41 | 42 | 44 | 45 | 46 | 47 | 47 |
| 171 | 40 | 41 | 42 | 43 | 44 | 45 | 46 | 47 | 48 |
| 172 | 41 | 41 | 42 | 43 | 44 | 46 | 47 | 47 | 48 |
| 173 | 41 | 42 | 42 | 43 | 45 | 46 | 47 | 48 | 48 |
| 174 | 41 | 42 | 43 | 44 | 45 | 46 | 47 | 48 | 49 |
| 175 | 42 | 42 | 43 | 44 | 45 | 47 | 48 | 49 | 49 |
| 176 | 42 | 43 | 43 | 44 | 46 | 47 | 48 | 49 | 50 |
| 177 | 42 | 43 | 44 | 45 | 46 | 47 | 49 | 49 | 50 |
| 178 | 43 | 43 | 44 | 45 | 46 | 48 | 49 | 50 | 50 |
| 179 | 43 | 44 | 44 | 45 | 47 | 48 | 49 | 50 | 51 |
| 180 | 43 | 44 | 45 | 46 | 47 | 48 | 50 | 50 | 51 |
| 181 | 44 | 44 | 45 | 46 | 47 | 49 | 50 | 51 | 52 |
| 182 | 44 | 45 | 45 | 46 | 48 | 49 | 50 | 51 | 52 |
| 183 | 44 | 45 | 46 | 47 | 48 | 49 | 51 | 52 | 52 |
| 184 | 45 | 45 | 46 | 47 | 48 | 50 | 51 | 52 | 53 |
| 185 | 45 | 46 | 46 | 47 | 49 | 50 | 51 | 52 | 53 |
| 186 | 45 | 46 | 47 | 48 | 49 | 51 | 52 | 53 | 53 |
| 187 | 46 | 46 | 47 | 48 | 49 | 51 | 52 | 53 | 54 |
| 188 | 46 | 46 | 47 | 48 | 50 | 51 | 53 | 53 | 54 |
| 189 | 46 | 47 | 47 | 49 | 50 | 52 | 53 | 54 | 54 |
| 190 | 46 | 47 | 48 | 49 | 50 | 52 | 53 | 54 | 55 |
| 191 | 47 | 47 | 48 | 49 | 51 | 52 | 54 | 54 | 55 |
| 192 | 47 | 48 | 48 | 50 | 51 | 53 | 54 | 55 | 55 |
| 193 | 47 | 48 | 49 | 50 | 51 | 53 | 54 | 55 | 56 |
| 194 | 48 | 48 | 49 | 50 | 52 | 53 | 55 | 55 | 56 |
| 195 | 48 | 49 | 49 | 51 | 52 | 54 | 55 | 56 | 56 |
| 196 | 48 | 49 | 50 | 51 | 52 | 54 | 55 | 56 | 57 |
| 197 | 49 | 49 | 50 | 51 | 53 | 54 | 56 | 56 | 57 |
| 198 | 49 | 49 | 50 | 51 | 53 | 54 | 56 | 57 | 57 |
| 199 | 49 | 50 | 50 | 52 | 53 | 55 | 56 | 57 | 58 |
| 200 | 49 | 50 | 51 | 52 | 54 | 55 | 57 | 57 | 58 |
| 201 | 50 | 50 | 51 | 52 | 54 | 55 | 57 | 58 | 58 |
| 202 | 50 | 51 | 51 | 53 | 54 | 56 | 57 | 58 | 59 |
| 203 | 50 | 51 | 52 | 53 | 54 | 56 | 57 | 58 | 59 |
| 204 | 50 | 51 | 52 | 53 | 55 | 56 | 58 | 59 | 59 |
| 205 | 51 | 51 | 52 | 54 | 55 | 57 | 58 | 59 | 60 |
| 206 | 51 | 52 | 52 | 54 | 55 | 57 | 58 | 59 | 60 |
| 207 | 51 | 52 | 53 | 54 | 56 | 57 | 59 | 60 | 60 |
| 208 | 52 | 52 | 53 | 54 | 56 | 58 | 59 | 60 | 61 |
| 209 | 52 | 53 | 53 | 55 | 56 | 58 | 59 | 60 | 61 |
| 210 | 52 | 53 | 54 | 55 | 57 | 58 | 60 | 61 | 61 |
| 211 | 52 | 53 | 54 | 55 | 57 | 58 | 60 | 61 | 62 |
| 212 | 53 | 53 | 54 | 56 | 57 | 59 | 60 | 61 | 62 |
| 213 | 53 | 54 | 54 | 56 | 57 | 59 | 61 | 61 | 62 |
| 214 | 53 | 54 | 55 | 56 | 58 | 59 | 61 | 62 | 63 |
| 215 | 53 | 54 | 55 | 56 | 58 | 60 | 61 | 62 | 63 |
| 216 | 54 | 54 | 55 | 57 | 58 | 60 | 61 | 62 | 63 |
| 217 | 54 | 55 | 55 | 57 | 59 | 60 | 62 | 63 | 64 |
| 218 | 54 | 55 | 56 | 57 | 59 | 60 | 62 | 63 | 64 |
| 219 | 54 | 55 | 56 | 57 | 59 | 61 | 62 | 63 | 64 |
| 220 | 55 | 55 | 56 | 58 | 59 | 61 | 63 | 64 | 64 |
| 221 | 55 | 56 | 57 | 58 | 60 | 61 | 63 | 64 | 65 |
| 222 | 55 | 56 | 57 | 58 | 60 | 62 | 63 | 64 | 65 |
| 223 | 55 | 56 | 57 | 59 | 60 | 62 | 64 | 64 | 65 |
| 224 | 56 | 56 | 57 | 59 | 60 | 62 | 64 | 65 | 66 |
| 225 | 56 | 57 | 58 | 59 | 61 | 62 | 64 | 65 | 66 |
| 226 | 56 | 57 | 58 | 59 | 61 | 63 | 64 | 65 | 66 |
| 227 | 56 | 57 | 58 | 60 | 61 | 63 | 65 | 66 | 67 |
| 228 | 57 | 57 | 58 | 60 | 62 | 63 | 65 | 66 | 67 |
| 229 | 57 | 58 | 59 | 60 | 62 | 64 | 65 | 66 | 67 |
| 230 | 57 | 58 | 59 | 60 | 62 | 64 | 65 | 66 | 67 |
| 231 | 57 | 58 | 59 | 61 | 62 | 64 | 66 | 67 | 68 |
| 232 | 58 | 58 | 59 | 61 | 63 | 64 | 66 | 67 | 68 |
| 233 | 58 | 59 | 60 | 61 | 63 | 65 | 66 | 67 | 68 |
| 234 | 58 | 59 | 60 | 61 | 63 | 65 | 67 | 68 | 69 |
| 235 | 58 | 59 | 60 | 62 | 63 | 65 | 67 | 68 | 69 |
| 236 | 59 | 59 | 60 | 62 | 64 | 65 | 67 | 68 | 69 |
| 237 | 59 | 60 | 61 | 62 | 64 | 66 | 67 | 68 | 69 |
| 238 | 59 | 60 | 61 | 62 | 64 | 66 | 68 | 69 | 70 |
| 239 | 59 | 60 | 61 | 63 | 64 | 66 | 68 | 69 | 70 |
| 240 | 60 | 60 | 61 | 63 | 65 | 67 | 68 | 69 | 70 |
| 241 | 60 | 61 | 62 | 63 | 65 | 67 | 68 | 70 | 70 |
| 242 | 60 | 61 | 62 | 63 | 65 | 67 | 69 | 70 | 71 |
| 243 | 60 | 61 | 62 | 64 | 65 | 67 | 69 | 70 | 71 |
| 244 | 61 | 61 | 62 | 64 | 66 | 68 | 69 | 70 | 71 |
| 245 | 61 | 62 | 62 | 64 | 66 | 68 | 70 | 71 | 72 |
| 246 | 61 | 62 | 63 | 64 | 66 | 68 | 70 | 71 | 72 |
| 247 | 61 | 62 | 63 | 65 | 66 | 68 | 70 | 71 | 72 |
| 248 | 61 | 62 | 63 | 65 | 67 | 69 | 70 | 71 | 72 |
| 249 | 62 | 62 | 63 | 65 | 67 | 69 | 71 | 72 | 73 |
| 250 | 62 | 63 | 64 | 65 | 67 | 69 | 71 | 72 | 73 |
| 251 | 62 | 63 | 64 | 66 | 67 | 69 | 71 | 72 | 73 |
| 252 | 62 | 63 | 64 | 66 | 68 | 70 | 71 | 72 | 73 |
| 253 | 63 | 63 | 64 | 66 | 68 | 70 | 72 | 73 | 74 |
| 254 | 63 | 64 | 65 | 66 | 68 | 70 | 72 | 73 | 74 |
| 255 | 63 | 64 | 65 | 66 | 68 | 70 | 72 | 73 | 74 |
| 256 | 63 | 64 | 65 | 67 | 69 | 71 | 72 | 74 | 74 |
| 257 | 63 | 64 | 65 | 67 | 69 | 71 | 73 | 74 | 75 |
| 258 | 64 | 64 | 65 | 67 | 69 | 71 | 73 | 74 | 75 |
| 259 | 64 | 65 | 66 | 67 | 69 | 71 | 73 | 74 | 75 |
| 260 | 64 | 65 | 66 | 68 | 70 | 72 | 73 | 75 | 76 |
| 261 | 64 | 65 | 66 | 68 | 70 | 72 | 74 | 75 | 76 |
| 262 | 64 | 65 | 66 | 68 | 70 | 72 | 74 | 75 | 76 |
| 263 | 65 | 66 | 67 | 68 | 70 | 72 | 74 | 75 | 76 |
| 264 | 65 | 66 | 67 | 69 | 71 | 73 | 74 | 76 | 77 |
| 265 | 65 | 66 | 67 | 69 | 71 | 73 | 75 | 76 | 77 |
| 266 | 65 | 66 | 67 | 69 | 71 | 73 | 75 | 76 | 77 |
| 267 | 66 | 66 | 67 | 69 | 71 | 73 | 75 | 76 | 77 |
| 268 | 66 | 67 | 68 | 69 | 71 | 73 | 75 | 77 | 78 |
| 269 | 66 | 67 | 68 | 70 | 72 | 74 | 76 | 77 | 78 |
| 270 | 66 | 67 | 68 | 70 | 72 | 74 | 76 | 77 | 78 |
| 271 | 66 | 67 | 68 | 70 | 72 | 74 | 76 | 77 | 78 |
| 272 | 67 | 67 | 69 | 70 | 72 | 74 | 76 | 78 | 79 |
| 273 | 67 | 68 | 69 | 71 | 73 | 75 | 77 | 78 | 79 |
| 274 | 67 | 68 | 69 | 71 | 73 | 75 | 77 | 78 | 79 |
| 275 | 67 | 68 | 69 | 71 | 73 | 75 | 77 | 78 | 79 |
| 276 | 67 | 68 | 69 | 71 | 73 | 75 | 77 | 79 | 80 |
| 277 | 68 | 69 | 70 | 71 | 73 | 76 | 78 | 79 | 80 |
| 278 | 68 | 69 | 70 | 72 | 74 | 76 | 78 | 79 | 80 |
| 279 | 68 | 69 | 70 | 72 | 74 | 76 | 78 | 79 | 80 |
| 280 | 68 | 69 | 70 | 72 | 74 | 76 | 78 | 80 | 81 |
| 281 | 68 | 69 | 70 | 72 | 74 | 77 | 79 | 80 | 81 |
| 282 | 69 | 70 | 71 | 72 | 75 | 77 | 79 | 80 | 81 |
| 283 | 69 | 70 | 71 | 73 | 75 | 77 | 79 | 80 | 81 |
| 284 | 69 | 70 | 71 | 73 | 75 | 77 | 79 | 81 | 82 |
| 285 | 69 | 70 | 71 | 73 | 75 | 77 | 80 | 81 | 82 |
| 286 | 69 | 70 | 71 | 73 | 76 | 78 | 80 | 81 | 82 |
| 287 | 70 | 71 | 72 | 74 | 76 | 78 | 80 | 81 | 82 |
| 288 | 70 | 71 | 72 | 74 | 76 | 78 | 80 | 82 | 83 |
| 289 | 70 | 71 | 72 | 74 | 76 | 78 | 80 | 82 | 83 |
| 290 | 70 | 71 | 72 | 74 | 76 | 79 | 81 | 82 | 83 |
| 291 | 70 | 71 | 72 | 74 | 77 | 79 | 81 | 82 | 83 |
| 292 | 71 | 72 | 73 | 75 | 77 | 79 | 81 | 82 | 84 |
| 293 | 71 | 72 | 73 | 75 | 77 | 79 | 81 | 83 | 84 |
| 294 | 71 | 72 | 73 | 75 | 77 | 80 | 82 | 83 | 84 |

Mean and variance equation for FL in males and females:

*E(Z*_i_) = 4.105732209910064 + [-344.2425559342237 GA_i_^-2^] + [0.0103936855030136 GA_i_^1^]

*Var(Z*_i_) = 0.0107011124191591 + [584.7103509618497 GA_i_^-4^] + [-4.456046516168862 GA_i_^-2^] + [-0.0004141491119216 GA_i_^1^] + [0.0502118462585746 GA_i_^-2^GA_i_^1^] + [4.83269392995e-06 GA_i_^2^]
