# Supplementary material for: Swedish intrauterine growth reference ranges of biometric measurements of fetal head, abdomen and femur
Source: Sci Rep. 2020 Dec 31;10:22441. doi: 10.1038/s41598-020-79797-8 (PMC7775468; doi:10.1038/s41598-020-79797-8)
Supplement: Supplementary file 11 — Supplementary Table 11. [file 41598_2020_79797_MOESM11_ESM.docx]

Supplementary Table 11a. Estimated biparietal diameter (BPD) in mm by gestational age (GA) for males and females, standard deviations (SD). The table only includes subjects with BMI 18.5 to 29.9 kg/m^2^.

| GA (weeks*) | -3 SD | -2 SD | -1 SD | Median | +1 SD | +2 SD | +3 SD |
| --- | --- | --- | --- | --- | --- | --- | --- |
| 12 | 19 | 19 | 20 | 20 | 21 | 21 | 22 |
| 13 | 22 | 22 | 23 | 23 | 24 | 25 | 25 |
| 14 | 25 | 25 | 26 | 27 | 27 | 28 | 29 |
| 15 | 28 | 29 | 29 | 30 | 31 | 32 | 33 |
| 16 | 31 | 32 | 33 | 34 | 34 | 35 | 36 |
| 17 | 34 | 35 | 36 | 37 | 38 | 39 | 40 |
| 18 | 37 | 38 | 39 | 41 | 42 | 43 | 45 |
| 19 | 40 | 41 | 43 | 44 | 46 | 47 | 49 |
| 20 | 43 | 44 | 46 | 48 | 49 | 51 | 53 |
| 21 | 46 | 48 | 49 | 51 | 53 | 55 | 57 |
| 22 | 49 | 51 | 52 | 54 | 56 | 58 | 60 |
| 23 | 52 | 54 | 56 | 58 | 60 | 62 | 64 |
| 24 | 55 | 57 | 59 | 61 | 63 | 66 | 68 |
| 25 | 57 | 59 | 62 | 64 | 66 | 69 | 72 |
| 26 | 60 | 62 | 65 | 67 | 70 | 72 | 75 |
| 27 | 62 | 65 | 67 | 70 | 73 | 75 | 78 |
| 28 | 65 | 67 | 70 | 73 | 75 | 78 | 81 |
| 29 | 67 | 70 | 73 | 75 | 78 | 81 | 84 |
| 30 | 70 | 72 | 75 | 78 | 81 | 84 | 87 |
| 31 | 72 | 74 | 77 | 80 | 83 | 86 | 90 |
| 32 | 74 | 77 | 79 | 82 | 86 | 89 | 92 |
| 33 | 76 | 79 | 82 | 85 | 88 | 91 | 95 |
| 34 | 77 | 80 | 83 | 87 | 90 | 93 | 97 |
| 35 | 79 | 82 | 85 | 88 | 92 | 95 | 99 |
| 36 | 81 | 84 | 87 | 90 | 93 | 97 | 101 |
| 37 | 82 | 85 | 88 | 92 | 95 | 99 | 102 |
| 38 | 83 | 86 | 90 | 93 | 96 | 100 | 104 |
| 39 | 84 | 87 | 91 | 94 | 98 | 102 | 105 |
| 40 | 85 | 88 | 92 | 95 | 99 | 103 | 107 |
| 41 | 86 | 89 | 93 | 96 | 100 | 104 | 108 |
| 42 | 86 | 90 | 93 | 97 | 101 | 105 | 109 |

*GA expressed as completed gestational weeks, e.g. 12 weeks corresponds to 12+0 weeks or 84 gestational days.

Supplementary Table 11b. Estimated biparietal diameter (BPD) in mm by gestational age (GA) for males and females, percentiles. The table only includes subjects with BMI 18.5 to 29.9 kg/m^2^.

| GA (weeks*) | 2.5th | 5th | 10th | 25th | Median | 75th | 90th | 95th | 97.5th |
| --- | --- | --- | --- | --- | --- | --- | --- | --- | --- |
| 12 | 19 | 19 | 20 | 20 | 20 | 21 | 21 | 21 | 21 |
| 13 | 22 | 23 | 23 | 23 | 23 | 24 | 24 | 24 | 24 |
| 14 | 25 | 26 | 26 | 26 | 27 | 27 | 28 | 28 | 28 |
| 15 | 29 | 29 | 29 | 30 | 30 | 31 | 31 | 31 | 32 |
| 16 | 32 | 32 | 32 | 33 | 34 | 34 | 35 | 35 | 35 |
| 17 | 35 | 35 | 36 | 36 | 37 | 38 | 38 | 39 | 39 |
| 18 | 38 | 39 | 39 | 40 | 41 | 41 | 42 | 43 | 43 |
| 19 | 41 | 42 | 42 | 43 | 44 | 45 | 46 | 46 | 47 |
| 20 | 45 | 45 | 46 | 46 | 48 | 49 | 50 | 50 | 51 |
| 21 | 48 | 48 | 49 | 50 | 51 | 52 | 53 | 54 | 55 |
| 22 | 51 | 51 | 52 | 53 | 54 | 56 | 57 | 58 | 58 |
| 23 | 54 | 54 | 55 | 56 | 58 | 59 | 60 | 61 | 62 |
| 24 | 57 | 57 | 58 | 59 | 61 | 62 | 64 | 65 | 65 |
| 25 | 60 | 60 | 61 | 62 | 64 | 66 | 67 | 68 | 69 |
| 26 | 62 | 63 | 64 | 65 | 67 | 69 | 70 | 71 | 72 |
| 27 | 65 | 66 | 67 | 68 | 70 | 72 | 73 | 74 | 75 |
| 28 | 68 | 68 | 69 | 71 | 73 | 75 | 76 | 77 | 78 |
| 29 | 70 | 71 | 72 | 73 | 75 | 77 | 79 | 80 | 81 |
| 30 | 72 | 73 | 74 | 76 | 78 | 80 | 82 | 83 | 84 |
| 31 | 75 | 75 | 77 | 78 | 80 | 82 | 84 | 85 | 86 |
| 32 | 77 | 78 | 79 | 80 | 82 | 85 | 87 | 88 | 89 |
| 33 | 79 | 80 | 81 | 83 | 85 | 87 | 89 | 90 | 91 |
| 34 | 80 | 81 | 83 | 84 | 87 | 89 | 91 | 92 | 93 |
| 35 | 82 | 83 | 84 | 86 | 88 | 91 | 93 | 94 | 95 |
| 36 | 84 | 85 | 86 | 88 | 90 | 92 | 94 | 96 | 97 |
| 37 | 85 | 86 | 87 | 89 | 92 | 94 | 96 | 97 | 98 |
| 38 | 86 | 87 | 89 | 91 | 93 | 95 | 97 | 99 | 100 |
| 39 | 87 | 89 | 90 | 92 | 94 | 97 | 99 | 100 | 101 |
| 40 | 88 | 89 | 91 | 93 | 95 | 98 | 100 | 101 | 103 |
| 41 | 89 | 90 | 92 | 94 | 96 | 99 | 101 | 103 | 104 |
| 42 | 90 | 91 | 92 | 95 | 97 | 100 | 102 | 104 | 105 |

*GA expressed as completed gestational weeks, e.g. 12 weeks corresponds to 12+0 weeks or 84 gestational days.

Mean and variance equation for BPD in males and females:

*E(Z*_i_) = -2.532597681336148 + [2.475025235294841 log(GA_i_)] + -[0.0510164715735025 GA_i_^1^]

*Var(Z*_i_) = 0.0832683981008089 + [0.0203875848515135 log(GA_i_)^2^] + -[0.0820118454729514 log(GA_i_)] + [0.003322667554359 GA_i_^1^] + [-0.0008354084071186 log(GA_i_)GA_i_^1^] + [0.0000355798375268 GA_i_^2^]
