# Supplementary material for: Swedish intrauterine growth reference ranges of biometric measurements of fetal head, abdomen and femur
Source: Sci Rep. 2020 Dec 31;10:22441. doi: 10.1038/s41598-020-79797-8 (PMC7775468; doi:10.1038/s41598-020-79797-8)
Supplement: Supplementary file 12 — Supplementary Table 12. [file 41598_2020_79797_MOESM12_ESM.docx]

Supplementary Table 12a. Estimated head circumference (HC) in mm by gestational age (GA) for males and females, standard deviations (SD). The table only includes subjects with BMI 18.5 to 29.9 kg/m^2^.

| GA (weeks*) | -3 SD | -2 SD | -1 SD | Median | +1 SD | +2 SD | +3 SD |
| --- | --- | --- | --- | --- | --- | --- | --- |
| 12 | 66 | 68 | 71 | 73 | 76 | 79 | 82 |
| 13 | 78 | 80 | 83 | 86 | 89 | 92 | 95 |
| 14 | 90 | 93 | 96 | 99 | 102 | 105 | 108 |
| 15 | 102 | 105 | 108 | 112 | 115 | 119 | 122 |
| 16 | 114 | 118 | 121 | 125 | 128 | 132 | 136 |
| 17 | 126 | 130 | 134 | 138 | 142 | 146 | 150 |
| 18 | 138 | 142 | 146 | 151 | 155 | 160 | 164 |
| 19 | 150 | 154 | 159 | 164 | 168 | 173 | 179 |
| 20 | 161 | 166 | 171 | 176 | 181 | 187 | 192 |
| 21 | 172 | 177 | 183 | 188 | 194 | 200 | 206 |
| 22 | 183 | 189 | 194 | 200 | 207 | 213 | 220 |
| 23 | 193 | 200 | 206 | 212 | 219 | 226 | 233 |
| 24 | 204 | 210 | 217 | 224 | 231 | 238 | 245 |
| 25 | 213 | 220 | 227 | 234 | 242 | 250 | 258 |
| 26 | 223 | 230 | 237 | 245 | 253 | 261 | 269 |
| 27 | 231 | 239 | 247 | 255 | 263 | 272 | 281 |
| 28 | 240 | 248 | 256 | 264 | 273 | 282 | 292 |
| 29 | 248 | 256 | 265 | 274 | 283 | 292 | 302 |
| 30 | 255 | 264 | 273 | 282 | 292 | 301 | 312 |
| 31 | 263 | 271 | 281 | 290 | 300 | 310 | 321 |
| 32 | 269 | 278 | 288 | 298 | 308 | 319 | 330 |
| 33 | 275 | 285 | 295 | 305 | 315 | 326 | 338 |
| 34 | 281 | 291 | 301 | 311 | 322 | 334 | 346 |
| 35 | 286 | 296 | 306 | 317 | 329 | 341 | 353 |
| 36 | 290 | 301 | 312 | 323 | 335 | 347 | 360 |
| 37 | 294 | 305 | 316 | 328 | 340 | 353 | 366 |
| 38 | 297 | 308 | 320 | 332 | 345 | 358 | 372 |
| 39 | 300 | 311 | 324 | 336 | 350 | 363 | 378 |
| 40 | 302 | 314 | 327 | 340 | 354 | 368 | 383 |
| 41 | 303 | 316 | 329 | 343 | 357 | 372 | 388 |
| 42 | 304 | 317 | 331 | 345 | 360 | 376 | 392 |

*GA expressed as completed gestational weeks, e.g. 12 weeks corresponds to 12+0 weeks or 84 gestational days.

Supplementary Table 12b. Estimated head circumference (HC) in mm by gestational age (GA) for males and females, percentiles. The table only includes subjects with BMI 18.5 to 29.9 kg/m^2^.

| GA (weeks*) | 2.5th | 5th | 10th | 25th | Median | 75th | 90th | 95th | 97.5th |
| --- | --- | --- | --- | --- | --- | --- | --- | --- | --- |
| 12 | 69 | 69 | 70 | 72 | 73 | 75 | 77 | 78 | 79 |
| 13 | 81 | 81 | 82 | 84 | 86 | 88 | 90 | 91 | 92 |
| 14 | 93 | 94 | 95 | 97 | 99 | 101 | 103 | 104 | 105 |
| 15 | 105 | 106 | 107 | 109 | 112 | 114 | 116 | 117 | 118 |
| 16 | 118 | 119 | 120 | 122 | 125 | 127 | 129 | 131 | 132 |
| 17 | 130 | 131 | 133 | 135 | 138 | 140 | 143 | 144 | 146 |
| 18 | 142 | 144 | 145 | 148 | 151 | 154 | 156 | 158 | 160 |
| 19 | 154 | 156 | 157 | 160 | 164 | 167 | 170 | 172 | 173 |
| 20 | 166 | 168 | 170 | 173 | 176 | 180 | 183 | 185 | 187 |
| 21 | 178 | 179 | 181 | 185 | 188 | 192 | 196 | 198 | 200 |
| 22 | 189 | 191 | 193 | 196 | 200 | 205 | 208 | 211 | 213 |
| 23 | 200 | 202 | 204 | 208 | 212 | 217 | 221 | 223 | 225 |
| 24 | 210 | 212 | 215 | 219 | 224 | 228 | 233 | 235 | 238 |
| 25 | 220 | 223 | 225 | 229 | 234 | 239 | 244 | 247 | 249 |
| 26 | 230 | 232 | 235 | 240 | 245 | 250 | 255 | 258 | 261 |
| 27 | 239 | 242 | 245 | 249 | 255 | 261 | 266 | 269 | 272 |
| 28 | 248 | 251 | 254 | 259 | 264 | 270 | 276 | 279 | 282 |
| 29 | 257 | 259 | 262 | 268 | 274 | 280 | 285 | 289 | 292 |
| 30 | 264 | 267 | 270 | 276 | 282 | 289 | 294 | 298 | 301 |
| 31 | 272 | 275 | 278 | 284 | 290 | 297 | 303 | 307 | 310 |
| 32 | 279 | 282 | 285 | 291 | 298 | 305 | 311 | 315 | 318 |
| 33 | 285 | 288 | 292 | 298 | 305 | 312 | 319 | 322 | 326 |
| 34 | 291 | 294 | 298 | 304 | 311 | 319 | 326 | 330 | 333 |
| 35 | 296 | 300 | 303 | 310 | 317 | 325 | 332 | 336 | 340 |
| 36 | 301 | 304 | 308 | 315 | 323 | 331 | 338 | 343 | 346 |
| 37 | 305 | 309 | 313 | 320 | 328 | 336 | 344 | 348 | 352 |
| 38 | 309 | 312 | 317 | 324 | 332 | 341 | 349 | 354 | 358 |
| 39 | 312 | 316 | 320 | 328 | 336 | 345 | 353 | 358 | 363 |
| 40 | 314 | 318 | 323 | 331 | 340 | 349 | 358 | 363 | 367 |
| 41 | 316 | 320 | 325 | 333 | 343 | 352 | 361 | 367 | 371 |
| 42 | 318 | 322 | 327 | 336 | 345 | 355 | 365 | 370 | 375 |

*GA expressed as completed gestational weeks, e.g. 12 weeks corresponds to 12+0 weeks or 84 gestational days.

Mean and variance equation for HC in males and females:

*E(Z*_i_) = 8.47832375855174 + [-14.36710990023983 GA_i_^-0.5^] + [-0.0002364650326715 GA_i_^2^]

*Var(Z*_i_) = 0.0212451871605619 + [0.2955332867096627 GA_i_^-1^] + [-0.1548407851124624 GA_i_^-0.5^] + [-9.92652317824e-06 GA_i_^2^] + [0.0000177649721301 GA_i_^-0.5^GA_i_^2^] + [1.68517689289e-09 GA_i_^4]^
