# Supplementary material for: Swedish intrauterine growth reference ranges of biometric measurements of fetal head, abdomen and femur
Source: Sci Rep. 2020 Dec 31;10:22441. doi: 10.1038/s41598-020-79797-8 (PMC7775468; doi:10.1038/s41598-020-79797-8)
Supplement: Supplementary file 13 — Supplementary Table 13. [file 41598_2020_79797_MOESM13_ESM.docx]

Supplementary Table 13a. Estimated mean abdominal diameter (MAD) in mm by gestational age (GA) for males and females, standard deviations (SD). The table only includes subjects with BMI 18.5 to 29.9 kg/m^2^.

| GA (weeks*) | -3 SD | -2 SD | -1 SD | Median | +1 SD | +2 SD | +3 SD |
| --- | --- | --- | --- | --- | --- | --- | --- |
| 12 | 15 | 16 | 17 | 18 | 19 | 20 | 21 |
| 13 | 19 | 20 | 21 | 22 | 23 | 24 | 25 |
| 14 | 22 | 23 | 24 | 25 | 26 | 28 | 29 |
| 15 | 26 | 27 | 28 | 29 | 30 | 32 | 33 |
| 16 | 29 | 30 | 32 | 33 | 34 | 36 | 37 |
| 17 | 32 | 34 | 35 | 37 | 38 | 40 | 42 |
| 18 | 36 | 37 | 39 | 41 | 42 | 44 | 46 |
| 19 | 39 | 41 | 43 | 44 | 46 | 48 | 50 |
| 20 | 42 | 44 | 46 | 48 | 50 | 52 | 55 |
| 21 | 46 | 48 | 50 | 52 | 54 | 57 | 59 |
| 22 | 49 | 51 | 53 | 56 | 58 | 61 | 63 |
| 23 | 52 | 55 | 57 | 59 | 62 | 65 | 68 |
| 24 | 56 | 58 | 61 | 63 | 66 | 69 | 72 |
| 25 | 59 | 61 | 64 | 67 | 70 | 73 | 76 |
| 26 | 62 | 65 | 67 | 70 | 73 | 77 | 80 |
| 27 | 65 | 68 | 71 | 74 | 77 | 81 | 84 |
| 28 | 68 | 71 | 74 | 77 | 81 | 84 | 88 |
| 29 | 71 | 74 | 77 | 81 | 85 | 88 | 92 |
| 30 | 74 | 77 | 81 | 84 | 88 | 92 | 96 |
| 31 | 77 | 80 | 84 | 88 | 92 | 96 | 100 |
| 32 | 79 | 83 | 87 | 91 | 95 | 100 | 104 |
| 33 | 82 | 86 | 90 | 94 | 99 | 103 | 108 |
| 34 | 85 | 89 | 93 | 97 | 102 | 107 | 112 |
| 35 | 87 | 91 | 96 | 101 | 106 | 111 | 116 |
| 36 | 90 | 94 | 99 | 104 | 109 | 114 | 120 |
| 37 | 92 | 97 | 102 | 107 | 112 | 118 | 124 |
| 38 | 94 | 99 | 104 | 110 | 116 | 122 | 128 |
| 39 | 97 | 102 | 107 | 113 | 119 | 125 | 132 |
| 40 | 99 | 104 | 110 | 116 | 122 | 129 | 136 |
| 41 | 101 | 107 | 113 | 119 | 125 | 132 | 139 |
| 42 | 103 | 109 | 115 | 122 | 128 | 136 | 143 |

*GA expressed as completed gestational weeks, e.g. 12 weeks corresponds to 12+0 weeks or 84 gestational days.

Supplementary Table 13b. Estimated mean abdominal diameter (MAD) in mm by gestational age (GA) for males and females, percentiles. The table only includes subjects with BMI 18.5 to 29.9 kg/m^2^.

| GA (weeks*) | 2.5th | 5th | 10th | 25th | Median | 75th | 90th | 95th | 97.5th |
| --- | --- | --- | --- | --- | --- | --- | --- | --- | --- |
| 12 | 16 | 16 | 17 | 17 | 18 | 19 | 19 | 20 | 20 |
| 13 | 20 | 20 | 20 | 21 | 22 | 22 | 23 | 23 | 24 |
| 14 | 23 | 24 | 24 | 25 | 25 | 26 | 27 | 27 | 28 |
| 15 | 27 | 27 | 28 | 28 | 29 | 30 | 31 | 31 | 32 |
| 16 | 30 | 31 | 31 | 32 | 33 | 34 | 35 | 35 | 36 |
| 17 | 34 | 34 | 35 | 36 | 37 | 38 | 39 | 39 | 40 |
| 18 | 37 | 38 | 38 | 39 | 41 | 42 | 43 | 43 | 44 |
| 19 | 41 | 41 | 42 | 43 | 44 | 46 | 47 | 48 | 48 |
| 20 | 44 | 45 | 46 | 47 | 48 | 50 | 51 | 52 | 52 |
| 21 | 48 | 49 | 49 | 51 | 52 | 53 | 55 | 56 | 56 |
| 22 | 51 | 52 | 53 | 54 | 56 | 57 | 59 | 60 | 61 |
| 23 | 55 | 55 | 56 | 58 | 59 | 61 | 63 | 64 | 65 |
| 24 | 58 | 59 | 60 | 61 | 63 | 65 | 67 | 68 | 69 |
| 25 | 61 | 62 | 63 | 65 | 67 | 69 | 71 | 72 | 73 |
| 26 | 65 | 66 | 67 | 68 | 70 | 72 | 74 | 76 | 77 |
| 27 | 68 | 69 | 70 | 72 | 74 | 76 | 78 | 79 | 80 |
| 28 | 71 | 72 | 73 | 75 | 77 | 80 | 82 | 83 | 84 |
| 29 | 74 | 75 | 76 | 79 | 81 | 83 | 86 | 87 | 88 |
| 30 | 77 | 78 | 80 | 82 | 84 | 87 | 89 | 91 | 92 |
| 31 | 80 | 81 | 83 | 85 | 88 | 90 | 93 | 94 | 96 |
| 32 | 83 | 84 | 86 | 88 | 91 | 94 | 96 | 98 | 100 |
| 33 | 86 | 87 | 89 | 91 | 94 | 97 | 100 | 102 | 103 |
| 34 | 89 | 90 | 92 | 94 | 97 | 101 | 104 | 105 | 107 |
| 35 | 92 | 93 | 95 | 97 | 101 | 104 | 107 | 109 | 111 |
| 36 | 94 | 96 | 97 | 100 | 104 | 107 | 111 | 112 | 114 |
| 37 | 97 | 98 | 100 | 103 | 107 | 111 | 114 | 116 | 118 |
| 38 | 100 | 101 | 103 | 106 | 110 | 114 | 117 | 119 | 121 |
| 39 | 102 | 104 | 106 | 109 | 113 | 117 | 121 | 123 | 125 |
| 40 | 105 | 106 | 108 | 112 | 116 | 120 | 124 | 126 | 128 |
| 41 | 107 | 109 | 111 | 115 | 119 | 123 | 127 | 130 | 132 |
| 42 | 109 | 111 | 113 | 117 | 122 | 126 | 130 | 133 | 135 |

*GA expressed as completed gestational weeks, e.g. 12 weeks corresponds to 12+0 weeks or 84 gestational days.

Mean and variance equation for MAD in males and females:

*E(Z*_i_) = 6.697289828686617 + [-44.36502903725841 GA_i_^-2^] + [-12.12435934780623 GA_i_^-0.5^]

*Var(Z*_i_) = 0.0439087781957087 + [642.1996779945787 GA_i_^-4^] + [9.168891019926379 GA_i_^-2]^ + [-0.4979074847976022 GA_i_^-0.5^] + [-28.97203210297014 GA_i_^-2^GA_i_^-0.5^] + [1.492977619173324 GA_i_^-1^]
