# Supplementary material for: Swedish intrauterine growth reference ranges of biometric measurements of fetal head, abdomen and femur
Source: Sci Rep. 2020 Dec 31;10:22441. doi: 10.1038/s41598-020-79797-8 (PMC7775468; doi:10.1038/s41598-020-79797-8)
Supplement: Supplementary file 14 — Supplementary Table 14. [file 41598_2020_79797_MOESM14_ESM.docx]

Supplementary Table 14a. Estimated abdominal circumference (AC) in mm by gestational age (GA) for males and females, standard deviations (SD). The table only includes subjects with BMI 18.5 to 29.9 kg/m^2^.

| GA (weeks*) | -3 SD | -2 SD | -1 SD | Median | +1 SD | +2 SD | +3 SD |
| --- | --- | --- | --- | --- | --- | --- | --- |
| 12 | 49 | 51 | 54 | 57 | 60 | 64 | 67 |
| 13 | 59 | 62 | 65 | 69 | 72 | 76 | 79 |
| 14 | 70 | 74 | 77 | 81 | 84 | 88 | 92 |
| 15 | 81 | 85 | 89 | 93 | 97 | 101 | 106 |
| 16 | 92 | 96 | 100 | 105 | 110 | 115 | 120 |
| 17 | 103 | 107 | 112 | 117 | 122 | 128 | 134 |
| 18 | 114 | 119 | 124 | 130 | 135 | 141 | 148 |
| 19 | 124 | 130 | 136 | 142 | 148 | 155 | 162 |
| 20 | 135 | 141 | 147 | 154 | 161 | 168 | 175 |
| 21 | 145 | 152 | 159 | 166 | 173 | 181 | 189 |
| 22 | 156 | 163 | 170 | 178 | 186 | 194 | 203 |
| 23 | 166 | 173 | 181 | 189 | 198 | 207 | 216 |
| 24 | 176 | 184 | 192 | 201 | 210 | 220 | 230 |
| 25 | 186 | 194 | 203 | 213 | 222 | 232 | 243 |
| 26 | 196 | 205 | 214 | 224 | 234 | 245 | 256 |
| 27 | 205 | 215 | 225 | 235 | 246 | 257 | 269 |
| 28 | 214 | 224 | 235 | 246 | 257 | 269 | 282 |
| 29 | 224 | 234 | 245 | 257 | 269 | 282 | 295 |
| 30 | 233 | 244 | 255 | 267 | 280 | 293 | 307 |
| 31 | 241 | 253 | 265 | 278 | 291 | 305 | 320 |
| 32 | 250 | 262 | 275 | 288 | 302 | 317 | 333 |
| 33 | 258 | 271 | 284 | 298 | 313 | 329 | 345 |
| 34 | 266 | 280 | 294 | 308 | 324 | 340 | 357 |
| 35 | 274 | 288 | 303 | 318 | 334 | 351 | 369 |
| 36 | 282 | 297 | 312 | 328 | 345 | 363 | 381 |
| 37 | 290 | 305 | 321 | 338 | 355 | 374 | 393 |
| 38 | 297 | 313 | 329 | 347 | 365 | 385 | 405 |
| 39 | 304 | 321 | 338 | 356 | 375 | 396 | 417 |
| 40 | 311 | 328 | 346 | 365 | 385 | 406 | 429 |
| 41 | 318 | 336 | 355 | 374 | 395 | 417 | 440 |
| 42 | 325 | 344 | 363 | 383 | 405 | 428 | 452 |

*GA expressed as completed gestational weeks, e.g. 12 weeks corresponds to 12+0 weeks or 84 gestational days.

Supplementary Table 14b. Estimated abdominal circumference (AC) in mm by gestational age (GA) for males and females, percentiles. The table only includes subjects with BMI 18.5 to 29.9 kg/m^2^.

| GA (weeks*) | 2.5th | 5th | 10th | 25th | Median | 75th | 90th | 95th | 97.5th |
| --- | --- | --- | --- | --- | --- | --- | --- | --- | --- |
| 12 | 51 | 52 | 53 | 55 | 57 | 59 | 61 | 62 | 63 |
| 13 | 63 | 63 | 65 | 67 | 69 | 71 | 73 | 74 | 76 |
| 14 | 74 | 75 | 76 | 78 | 81 | 83 | 86 | 87 | 88 |
| 15 | 85 | 86 | 88 | 90 | 93 | 96 | 98 | 100 | 101 |
| 16 | 96 | 98 | 99 | 102 | 105 | 108 | 111 | 113 | 114 |
| 17 | 108 | 109 | 111 | 114 | 117 | 121 | 124 | 126 | 128 |
| 18 | 119 | 121 | 122 | 126 | 130 | 133 | 137 | 139 | 141 |
| 19 | 130 | 132 | 134 | 138 | 142 | 146 | 150 | 152 | 154 |
| 20 | 141 | 143 | 145 | 149 | 154 | 158 | 163 | 165 | 168 |
| 21 | 152 | 154 | 157 | 161 | 166 | 171 | 175 | 178 | 181 |
| 22 | 163 | 165 | 168 | 173 | 178 | 183 | 188 | 191 | 194 |
| 23 | 174 | 176 | 179 | 184 | 189 | 195 | 200 | 204 | 207 |
| 24 | 184 | 187 | 190 | 195 | 201 | 207 | 213 | 216 | 219 |
| 25 | 195 | 197 | 201 | 206 | 213 | 219 | 225 | 229 | 232 |
| 26 | 205 | 208 | 211 | 217 | 224 | 231 | 237 | 241 | 244 |
| 27 | 215 | 218 | 222 | 228 | 235 | 242 | 249 | 253 | 257 |
| 28 | 225 | 228 | 232 | 238 | 246 | 254 | 261 | 265 | 269 |
| 29 | 235 | 238 | 242 | 249 | 257 | 265 | 272 | 277 | 281 |
| 30 | 244 | 248 | 252 | 259 | 267 | 276 | 284 | 289 | 293 |
| 31 | 253 | 257 | 262 | 269 | 278 | 287 | 295 | 300 | 305 |
| 32 | 262 | 266 | 271 | 279 | 288 | 298 | 306 | 312 | 316 |
| 33 | 271 | 276 | 280 | 289 | 298 | 308 | 317 | 323 | 328 |
| 34 | 280 | 285 | 290 | 298 | 308 | 319 | 328 | 334 | 339 |
| 35 | 289 | 293 | 299 | 308 | 318 | 329 | 339 | 345 | 351 |
| 36 | 297 | 302 | 307 | 317 | 328 | 339 | 350 | 356 | 362 |
| 37 | 305 | 310 | 316 | 326 | 338 | 349 | 360 | 367 | 373 |
| 38 | 314 | 319 | 325 | 335 | 347 | 359 | 371 | 378 | 384 |
| 39 | 321 | 327 | 333 | 344 | 356 | 369 | 381 | 388 | 395 |
| 40 | 329 | 335 | 341 | 352 | 365 | 379 | 391 | 399 | 406 |
| 41 | 337 | 343 | 349 | 361 | 374 | 388 | 401 | 409 | 416 |
| 42 | 344 | 350 | 357 | 369 | 383 | 398 | 411 | 419 | 427 |

*GA expressed as completed gestational weeks, e.g. 12 weeks corresponds to 12+0 weeks or 84 gestational days.

Mean and variance equation for AC in males and females:

*E(Z*_i_) = 7.790954230375379 + [-50.92411353805426 GA_i_^-2^] + [-11.75251895675697 GA_i_^-0.5^]

*Var(Z*_i_) = 0.032918912952702 + [421.9679782167028 GA_i_^-4^] + [6.104940190961744 GA_i_^-2^] + [-0.3580943393795534 GA_i_^-0.5^] + [-19.27811870041735 GA_i_^-2^GA_i_^-0.5^] + [1.054372318247067 GA_i_^-1^]
