# Supplementary material for: Swedish intrauterine growth reference ranges of biometric measurements of fetal head, abdomen and femur
Source: Sci Rep. 2020 Dec 31;10:22441. doi: 10.1038/s41598-020-79797-8 (PMC7775468; doi:10.1038/s41598-020-79797-8)
Supplement: Supplementary file 15 — Supplementary Table 15. [file 41598_2020_79797_MOESM15_ESM.docx]

Supplementary Table 15a. Estimated femur length (FL) in mm by gestational age (GA) for males and females, standard deviations (SD). The table only includes subjects with BMI 18.5 to 29.9 kg/m^2^.

| GA (weeks*) | -3 SD | -2 SD | -1 SD | Median | +1 SD | +2 SD | +3 SD |
| --- | --- | --- | --- | --- | --- | --- | --- |
| 12 | 5 | 5 | 6 | 6 | 7 | 8 | 9 |
| 13 | 7 | 8 | 8 | 9 | 10 | 11 | 12 |
| 14 | 10 | 10 | 11 | 12 | 13 | 14 | 15 |
| 15 | 13 | 14 | 14 | 15 | 16 | 17 | 18 |
| 16 | 16 | 17 | 18 | 19 | 20 | 21 | 22 |
| 17 | 19 | 20 | 21 | 22 | 23 | 24 | 26 |
| 18 | 22 | 23 | 24 | 25 | 27 | 28 | 29 |
| 19 | 25 | 26 | 27 | 28 | 30 | 31 | 33 |
| 20 | 28 | 29 | 30 | 32 | 33 | 35 | 36 |
| 21 | 30 | 32 | 33 | 35 | 36 | 38 | 39 |
| 22 | 33 | 34 | 36 | 37 | 39 | 41 | 43 |
| 23 | 35 | 37 | 39 | 40 | 42 | 44 | 46 |
| 24 | 38 | 39 | 41 | 43 | 45 | 47 | 49 |
| 25 | 40 | 42 | 44 | 45 | 47 | 49 | 52 |
| 26 | 42 | 44 | 46 | 48 | 50 | 52 | 54 |
| 27 | 44 | 46 | 48 | 50 | 52 | 55 | 57 |
| 28 | 46 | 48 | 50 | 52 | 55 | 57 | 59 |
| 29 | 48 | 50 | 52 | 54 | 57 | 59 | 62 |
| 30 | 50 | 52 | 54 | 57 | 59 | 61 | 64 |
| 31 | 52 | 54 | 56 | 59 | 61 | 64 | 66 |
| 32 | 53 | 56 | 58 | 60 | 63 | 66 | 68 |
| 33 | 55 | 57 | 60 | 62 | 65 | 68 | 71 |
| 34 | 57 | 59 | 62 | 64 | 67 | 70 | 73 |
| 35 | 58 | 61 | 63 | 66 | 69 | 72 | 75 |
| 36 | 60 | 62 | 65 | 68 | 70 | 73 | 76 |
| 37 | 61 | 64 | 67 | 69 | 72 | 75 | 78 |
| 38 | 63 | 65 | 68 | 71 | 74 | 77 | 80 |
| 39 | 64 | 67 | 70 | 73 | 76 | 79 | 82 |
| 40 | 65 | 68 | 71 | 74 | 77 | 81 | 84 |
| 41 | 67 | 70 | 73 | 76 | 79 | 82 | 86 |
| 42 | 68 | 71 | 74 | 77 | 80 | 84 | 88 |

*GA expressed as completed gestational weeks, e.g. 12 weeks corresponds to 12+0 weeks or 84 gestational days.

Supplementary Table 15b. Estimated femur length (FL) in mm by gestational age (GA) for males and females, percentiles. The table only includes subjects with BMI 18.5 to 29.9 kg/m^2^.

| GA (weeks*) | 2.5th | 5th | 10th | 25th | Median | 75th | 90th | 95th | 97.5th |
| --- | --- | --- | --- | --- | --- | --- | --- | --- | --- |
| 12 | 5 | 5 | 5 | 6 | 6 | 7 | 7 | 7 | 8 |
| 13 | 8 | 8 | 8 | 9 | 9 | 10 | 10 | 10 | 11 |
| 14 | 11 | 11 | 11 | 12 | 12 | 13 | 13 | 14 | 14 |
| 15 | 14 | 14 | 14 | 15 | 15 | 16 | 17 | 17 | 17 |
| 16 | 17 | 17 | 17 | 18 | 19 | 19 | 20 | 20 | 21 |
| 17 | 20 | 20 | 21 | 21 | 22 | 23 | 23 | 24 | 24 |
| 18 | 23 | 23 | 24 | 24 | 25 | 26 | 27 | 27 | 28 |
| 19 | 26 | 26 | 27 | 28 | 28 | 29 | 30 | 31 | 31 |
| 20 | 29 | 29 | 30 | 31 | 32 | 33 | 33 | 34 | 34 |
| 21 | 32 | 32 | 33 | 34 | 35 | 36 | 37 | 37 | 38 |
| 22 | 34 | 35 | 35 | 36 | 37 | 39 | 40 | 40 | 41 |
| 23 | 37 | 38 | 38 | 39 | 40 | 41 | 42 | 43 | 44 |
| 24 | 39 | 40 | 41 | 42 | 43 | 44 | 45 | 46 | 47 |
| 25 | 42 | 42 | 43 | 44 | 45 | 47 | 48 | 49 | 49 |
| 26 | 44 | 45 | 45 | 46 | 48 | 49 | 50 | 51 | 52 |
| 27 | 46 | 47 | 47 | 49 | 50 | 52 | 53 | 54 | 54 |
| 28 | 48 | 49 | 50 | 51 | 52 | 54 | 55 | 56 | 57 |
| 29 | 50 | 51 | 52 | 53 | 54 | 56 | 57 | 58 | 59 |
| 30 | 52 | 53 | 54 | 55 | 57 | 58 | 60 | 61 | 61 |
| 31 | 54 | 55 | 56 | 57 | 59 | 60 | 62 | 63 | 64 |
| 32 | 56 | 56 | 57 | 59 | 60 | 62 | 64 | 65 | 66 |
| 33 | 57 | 58 | 59 | 61 | 62 | 64 | 66 | 67 | 68 |
| 34 | 59 | 60 | 61 | 62 | 64 | 66 | 68 | 69 | 70 |
| 35 | 61 | 62 | 63 | 64 | 66 | 68 | 69 | 71 | 71 |
| 36 | 62 | 63 | 64 | 66 | 68 | 70 | 71 | 72 | 73 |
| 37 | 64 | 65 | 66 | 67 | 69 | 71 | 73 | 74 | 75 |
| 38 | 65 | 66 | 67 | 69 | 71 | 73 | 75 | 76 | 77 |
| 39 | 67 | 68 | 69 | 71 | 73 | 75 | 76 | 78 | 79 |
| 40 | 68 | 69 | 70 | 72 | 74 | 76 | 78 | 79 | 80 |
| 41 | 70 | 71 | 72 | 74 | 76 | 78 | 80 | 81 | 82 |
| 42 | 71 | 72 | 73 | 75 | 77 | 79 | 81 | 83 | 84 |

*GA expressed as completed gestational weeks, e.g. 12 weeks corresponds to 12+0 weeks or 84 gestational days.

Mean and variance equation for FL in males and females:

*E(Z*_i_) = 4.110025582531111 + [-344.9910344684234 GA_i_^-2^] + [0.0102829471292403 GA_i_^1^]

*Var(Z*_i_) = 0.0097924954307523 + [512.6040678632028 GA_i_^-4^] + [-3.87713970912606 GA_i_^-2^] + [-0.0003676349433194 GA_i_^1^] + [0.0432277067365003 GA_i_^-2^GA_i_^1^] + [4.17828182957e-06 GA_i_^2^]
